# Supplementary material for: Effect of enhanced peer PrEP referral with HIV self-testing delivery among young Kenyan women: A randomized controlled trial of peer networks
Source: PLoS Med. 2026 Mar 30;23(3):e1005023. doi: 10.1371/journal.pmed.1005023 (PMC13046272; doi:10.1371/journal.pmed.1005023)
Supplement: S1 Protocol — (PDF) [file pmed.1005023.s002.pdf]

Peer PrEP referral + HIV self-test delivery for PrEP initiation among young Kenyan women:

Pilot study & randomized implementation trial

Version 1.11
May 22, 2025

Funding:

United States National Institute of Mental Health (NIMH)

Key Changes:

Version 1.6 (January 2023): Refine trial procedures following the pilot feasibility study and qualitative assessment.

Version 1.7 (May 2023): [Prior to peer provider enrollment] Clarify additional peer provider (index peer) eligibility criteria:

- Peer providers must have used PrEP within the last year.
- Peer providers must not be enrolled in another HIV study.

Version 1.8 (June 2023): Eliminate the restriction of using only the Sure Check HIVST brand and enable us to use other WHO-approved brands of blood-based HIVST kits in case of supply chain challenges or short expiry.

Version 1.9 (July 2023): [Prior to peer client enrollment] Clarify peer client (referred peer) ineligibility criteria:

- Peer clients are not ineligible if they are already using PrEP when approached by their index peer for referral.

Version 1.10 (October 2023): Clarify additional peer provider (index peer) ineligibility criteria (being illiterate). Also, to increase peer client (referred peer) enrollment we:

- Increased the incentive for contacting study staff to provide contact information at the time of PrEP referral (from 50 to 150 KES).
- Obtained approval to collect peer client contact information from the peer provider during their follow-up visit.

Version 1.11 (May 2025): Align trial outcomes with final version of the Statistical Analysis Plan **[SAP finalized July 2024, prior to trial unblinding];** edits to align the finalized outcomes on ClinicalTrials.gov were also made at this time.

- Primary: PrEP initiation
- Secondary: Recent HIV testing; PrEP continuation; PrEP adherence
- Process: Number of peers referred; linkage to care; HIVST use
- Implementation: Acceptability; feasibility; appropriateness; fidelity; cost.

PROTOCOL TEAM

Fred Hutchinson Cancer Research Center, Seattle, USA

Katrina Ortblad, ScD, MPH (PI)
Rachel Malen, MPH (Project Manager)
Stephanie Roche, PhD (Co-Investigator)
Adriana Reedy, MS (Project Coordinator)
Carlos Culquichicon, MSPH, MD (Research Assistant)

University of Washington, Seattle, USA

Jared Baeten, MD, PhD (Co-investigator)
Kenneth Sherr, PhD, MPH (Co-investigator)
Ruanne Barnabas, DPhil, MBChB, MSc (Co-investigator)
Jane Simoni, PhD (Co-investigator)

Jomo Kenyatta University of Agriculture and Technology Nairobi, Kenya

Kenneth Ngure, PhD, MPH, MSc (Site Principal Investigator)

Kenya Medical Research Institute, Nairobi, Kenya

Nelly R. Mugo, MBChB, MMed, MPH (Co-investigator)

National AIDS & STI Control Programme, Nairobi, Kenya

Mary Mugambi, BA (Consultant)

Heidelberg University

Maureen McGowan, MS (Research Assistant)

Partners in Health and Research Development, Thika, Kenya

Njeri Wairimu, B.A. (Project Coordinator)
Catherine Kiptinness BPharm, MPH (Site Coordinator)

Nairobi County Health Management Services

Antony Kiplagat, MSc (Co-investigator)

TABLE OF CONTENTS

ABREVIATIONS **6**
PROTOCOL SUMMARY **7**
LAY SUMMARY **8**
ABSTRACT **8**
BACKGROUND **9**
Study Objectives 13
Research Questions 13
Overview- Study Design 14
Setting 14
Aim 1: Pilot study procedures 14
HIV Self-Testing Kit 17
Data Collection 18
Data Analysis & Outcomes 18
Participant retention and withdrawal 19
Limitations 19
Aim 2: Randomization Trial Procedures 20
Study Population 20
HIV Self-Testing Kit 30
Randomization 22
SAFETY **31**
PrEP 31
Pregnancy 31
Social harm considerations 31
HUMAN SUBJECTS CONSIDERATIONS **32**
Study oversight 32
Risks 32
Protection against risk 33
Benefits 33
Care for persons identified as HIV infected 33
Benefits to the community 33
Importance of the knowledge to be gained 34
Treatment for injury 34
Study records 34
Confidentiality 34

Dissemination Plan 34
TIMELINE 36
REFERENCES 37

ABBREVIATIONS

3TC	Lamivudine
AE	Adverse Event
AGYW	Adolescent girls and young women
ART	Antiretroviral Therapy
DAIDS	Division of AIDS (NIH)
DBS	Dried Blood Spots
EC	Ethics Committee
FDA	Food and Drug Administration (US)
FGD	Focus Group Discussion
FSW	Female sex worker
FTC	Emtricitabine
FTC-TP	Emtricitabine-triphosphate
HCW	Healthcare worker
HIVST	HIV Self-testing
IEC	Information, communication, education
IRB	Institutional Review Board
KEMRI	Kenya Medical Research Institute
MoH	Ministry of Health
MSM	Men who have sex with men
NGT	Nominal group technique
NIH	National Institutes of Health (US)
PHRD	Partners in Health & Research Development
PrEP	Pre-exposure prophylaxis
RA	Research assistant
SAE	Serious adverse event
TDF	Tenofovir
TFV-DP	Tenofovir diphosphate
UNAIDS	Joint United Nations Program on HIV/AIDS
WHO	World Health Organization
NASCOP	National AIDS and STIs Control Programme

PROTOCOL SUMMARY

Pre-exposure prophylaxis (PrEP) for HIV prevention is highly effective. In Kenya, a country with the 4th largest HIV epidemic worldwide, the government began delivering PrEP on a national scale in 2017. One of the target groups for HIV incidence reduction is young women (16-24 years), who account for 33% of the total of new HIV infections in Kenya yet comprise only 10% of the population. To date, PrEP initiation rates are low among young women. Barriers to PrEP initiation are multi-faceted and include institutional (e.g., stigma associated with use), and intra-personal (e.g., lack of PrEP knowledge or self-efficacy) barriers. Thus, innovative PrEP delivery models that can help overcome these barriers – and that are affordable to young women and health systems – are needed.

The opinion of peers often influences the behaviors and preferences of young women, globally and in Kenya, including opinions related to health and health care. Peer referrals or peer-delivered interventions have been commonly used among key populations with tight social connectivity to increase identification of new individuals living with HIV and facilitate linkage to treatment but have not yet been used to increase identification of individuals eligible for HIV prevention and facilitate linkage to interventions like PrEP. We hypothesize that a formalized peer PrEP referral + HIVST delivery model can amplify screening for HIV and PrEP initiation among young Kenyan women at HIV risk. Specifically, this study aims to:

Aim 1: Pilot and refine a peer PrEP referral + HIVST delivery model

<u>Approach</u>: Pilot the aim 1 delivery model among 16 young female PrEP users in Thika, Kenya, who will deliver formalized peer PrEP referral + HIVSTs to ~4 peers each (=64 peers total). One month later, we aim to measure model adoption (e.g., peer referral, HIVST use, and PrEP initiation) through quantitative measures. We also aim to understand model acceptability and model weak points for refinement through qualitative data collection using CFIR-informed focus group discussions (FGDs) with those delivering and receiving the intervention (4-5 FGDs, 3-6 participant/FGD).

<u>Hypothesis</u>: The proposed model will be adopted and accepted by PrEP users and peers, feasible, and easily refined to address identified weak points.

Aim 2: Conduct a hybrid effectiveness-implementation randomized trial to test the effect of the refined peer PrEP referral + HIVST delivery model on PrEP initiation among young Kenyan women

<u>Approach</u>: Conduct a 2-arm trial in Thika, Kenya where ~80 young female PrEP users are randomized to: (1) "formal peer PrEP referral + HIVST delivery", where they are encouraged to refer 4 peers using PrEP materials (developed in Aim 1) and distribute HIVST kits (2/peer, = 8 total), vs. (2) "informal peer PrEP referral", where they are encouraged to refer 4 peers to PrEP services by word-of-mouth, as is ongoing in Kenya. All outcomes will be measured among peers and PrEP users three months later. Outcomes will include adoption (e.g., PrEP referral, PrEP retention, HIVST use), fidelity, cost, and safety. The effectiveness outcome will be PrEP initiation (primary), confirmed using dried blood spots.

<u>Hypothesis</u>: The formal referral model will result in higher PrEP adoption (e.g., initiation and retention) among peers compared to the informal referral model and will have low cost and high fidelity.

This study will provide important information on how to increase PrEP initiation among young Kenyan women at high HIV risk and will inform a future proposal for a community-randomized trial that tests the peer PrEP referral + HIVST delivery model at scale.

LAY SUMMARY

Few young women at risk of HIV infection are initiating pre-exposure prophylaxis (PrEP) for HIV prevention in Kenya, thus we propose developing, refining, and testing a new model to increase PrEP initiation among young women at high HIV risk that has not yet been explored: peer PrEP referral + HIV self-testing (HIVST) delivery. Through a pilot based on qualitative formative research and stakeholder engagement, we will develop and refine an acceptable and feasible model where young (≥16 to 24 years) female PrEP users refer their peers to PrEP using materials we determine to be appropriate (e.g., pamphlets) and HIVSTs. We hypothesize that relative to informal word-of-mouth peer PrEP referral (ongoing in Kenya), formalized peer PrEP referral + HIVST delivery will increase PrEP adoption (i.e., initiation, retention, and adherence) among peers, and be low cost and have high fidelity in Kenya.

ABSTRACT

PrEP for HIV prevention is highly effective. However, in Kenya, where the government offers PrEP free of charge, few young women (16-24 years old) at high HIV risk are initiating PrEP. We propose piloting and testing an innovative model that might overcome barriers to PrEP initiation among young Kenyan women: peer PrEP referral + HIVST delivery. The specific aims of the proposed research are to: 1) pilot a delivery model based on formative research and refine the model using focus group discussions informed by implementation frameworks (Year 1 and 2) test the effect and implementation of the refined delivery model on PrEP initiation and other outcomes (including PrEP adoption, model fidelity, and costs) using a hybrid-randomized implementation trial (Years 2-3). This proposed research addresses one of the greatest challenges to PrEP scale-up today and will inform a National Institutes of Health (NIH) R01 proposal for a community-randomized trial and budget impact analysis.

BACKGROUND

Importance of the problem

More than two million persons become newly infected with HIV each year, the majority in Africa.[1] In Kenya, more than 1.5 million people are living with HIV[2] making it the country with the 4th largest epidemic. The past five years have witnessed major strides in the development of highly effective HIV prevention interventions, particularly using antiretroviral medications: antiretroviral therapy (ART) to decrease infectiousness and pre-exposure prophylaxis (PrEP) to prevent acquisition. Novel models to deliver these strategies successfully and efficiently, at scale, are needed to achieve maximum HIV prevention impact.

Young Kenyan women are at high risk of HIV

Kenyan adolescent girls and young women (AGYW) remain at particularly high risk of HIV infection. AGYW ages 16-24 years accounted for 33% (n= 23,312) of total new HIV infections in 2015, thereby being nearly twice as likely to acquire HIV compared to their male counterparts.[3] This discrepancy is associated in part with perceptions of HIV risk, lack of knowledge and/or access to effective HIV prevention interventions, and high prevalence of experienced sexual violence[4,5] – it is estimated that 33% of young women in Kenya have been raped before the age of 18 years.[6]

PrEP is an effective, recommended, and impactful strategy for HIV prevention

PrEP has been demonstrated to be efficacious and safe for reducing HIV risk among men who have sex with men (MSM),[7] heterosexual men and women,[8,9] and injection drug users[10] in diverse geographic settings. In 2012, the U.S. Food and Drug Administration (FDA) approved combination tenofovir disoproxil fumarate/emtricitabine (TDF/FTC) as the first medication with a label indication for HIV prevention in adults[11]. In 2015, the World Health Organization (WHO) issued guidance recommending TDF-containing PrEP as an additional prevention option for all persons at high risk for acquiring HIV [12] and in 2017, Kenya added PrEP to its national HIV prevention portfolio and began distributing free government-issued PrEP to all individuals at HIV risk at select public clinics.[13,14]

Adherence is essential for PrEP efficacy. PrEP clinical trials had a wide range of results for estimates of PrEP's efficacy for HIV protection – explained by the degree to which the trial populations were adherent to PrEP[15]. Secondary analyses from clinical trials and demonstration studies have shown that PrEP is highly efficacious and safe when taken as prescribed: at the individual level, HIV protection is on the order of 90-100% in both MSM and heterosexual populations when PrEP adherence was high, as measured by the presence and quantity of PrEP in blood samples.[8,16,17] PrEP adherence and HIV prevention effectiveness have been higher than in prior clinical trials, in many cases very high, in open-label demonstration projects among HIV sero-discordant couples, MSM, and young women at HIV risk,[18–20] which has been hypothesized to be a result of offering a strategy with demonstrated safety and effectiveness and without a placebo. In those PrEP demonstration studies, HIV incidence has been very low, and visits were generally quarterly and brief, suggesting that many who initiate PrEP in the context of known safety and efficacy may not need intensive follow-up to achieve high adherence. In some high income settings (e.g., San Francisco), delivery of PrEP at scale, layered onto high coverage of HIV testing and ART delivery, has resulted in substantial reductions in new HIV infections in the past five years.[21–23]

HIV self-testing (HIVST) is an effective and recommended strategy to increase HIV testing

In 2016, the WHO released a guideline which strongly recommended the implementation of HIVST as an HIV testing strategy. HIVST was recommended to support the so called "first 90" of the Joint United Nations Program on HIV/AIDS (UNAIDS) 90-90-90 targets (e.g., 90% of all people living with HIV will know their status by 2020). HIVST has found to be appropriate at increasing HIV testing uptake and

frequency among diverse populations (e.g., key populations, sero-discordant couples, men, and women) in a range of countries, including Kenya.[24] Additionally, HIVST may increase self-efficacy (e.g., knowing one's HIV status), knowledge of partner's HIV status (one's personal risk), and awareness of one's PrEP eligibility (when HIV-negative).

<u>Diverse models for PrEP referral + HIVST are needed</u>

In 2017, Kenya added PrEP to its national HIV prevention portfolio and began distributing free government-issued PrEP to all individuals at HIV risk at select public clinics.[13,14] While PrEP scale up in Kenya has been relatively easy to implement among key populations (e.g., men who have sex with men [MSM] and female sex workers [FSWs]) it has been difficult to scale up among AGYW at HIV risk. A study conducted in Kenya among young women (16-20 years) which screened HIV risk and offered PrEP, found that only 5% (9/168) eligible for PrEP initiated it.[25] Barriers to PrEP use among AGYW included lack of PrEP knowledge, mistrust about PrEP efficacy and safety, low HIV risk perception, and community-level barriers (e.g., stigma associated with PrEP use).[26]

<u>In Kenya, young women have tight social networks that often influence health behavior</u>

Peer-delivered HIV prevention interventions have been found to be effective and feasible among key populations with high social connectivity (e.g., MSM[27,28] and FSWs[29,30,31,32,33]). Similarly, young women have tight peer networks and are often influenced by one another in terms of healthcare behavior. In a HPTN-082 study conducted among AGYW in South Africa and Zimbabwe, 62% (254/409) of participants reported being encouraged to uptake PrEP by peers.[14] Similarly, in Kenya, informal peer PrEP referral (e.g., word-of-mouth referral) was a major facilitator to PrEP uptake among AGYW.

<u>Peer PrEP referral + HIVST delivery is a novel approach that could reach AGYW at risk of HIV</u>

This pilot study and subsequent hybrid-randomized implementation trial for peer PrEP referral + HIVST delivery will be the first model of its kind in sub-Saharan Africa. This approach is novel in its aim to build upon existing informal (word-of-mouth) referral systems and its aim to function within existing peer networks. This pilot study will be unique in its: 1) utilization of PrEP and HIVST- two innovations that have been recently introduced by the Kenyan Ministry of Health (MoH), 2) training of PrEP users as "PrEP champions", 3) implementation of HIVST to facilitate peer PrEP referral, and 4) implementation of a hybrid-effectiveness randomization trial. The perceived acceptability and feasibility of this study are supported by the formative stage of this research study (e.g., in-depth interviews and stakeholder meetings) developed through a K99 grant.

<u>Findings from formative research on peer PrEP referral + HIVST</u>

As formative research for this project, in the first half of 2021, we conducted in-depth interviews with AGYW using (n=15) and not using (n=15) PrEP to understand their perspectives and attitudes towards HIV, HIVST, PrEP, and COVID-19. We additionally asked about their acceptability towards a peer PrEP referral + HIVST delivery intervention. The median age of participants was 20 years (IQR: 20-22). Most participants were in a relationship but unmarried (n=22; 73%) and nearly half (n=14; 47%) had one or more children. 24 participants (80%) had tested for HIV within the last 3 months.

The main findings included that AGYW were often aware of HIVST although many had not used HIVST. While some AGYW reported interest in HIVST for reasons including learning one's HIV status privately and mitigating structural barriers associated with facility-based HIV testing (e.g., transportation costs, long waiting times); other AGYW described reluctance accepting HIVST due to perceived lack of knowledge and skills to perform the test accurately. Second, many AGYW were aware of PrEP although rampant misinformation was evident. AGYW described that they (or their communities) often mistook PrEP for post-exposure prophylaxis (PEP) or ARTs. Thereby stigmatizing PrEP use as it was associated

with persons engaging in risk behaviors or being HIV-positive. Third, we found that peer-delivered HIV prevention interventions may be of increased relevance following the COVID-19 pandemic as some AGYW reported changing partners during lockdowns while others described PrEP attrition during COVID-19 due to less frequent sexual activity and thereby lower perceived risk of HIV infection. Most importantly however, we found that most AGYW believed peer PrEP referral and HIVST delivery to be largely acceptable among Kenyan AGYW. Many participants expressed willingness to educate peers about PrEP use and side effects, to assist peers with HIVST and results interpretation, and to support peers with linkage to PrEP care and adherence. However, it should be noted that some participants mistrusted being approached by peers during a peer PrEP referral + HIVST model due to fear of involuntary HIV status or PrEP disclosure and its associated stigmatization. Example quotes highlighting findings above are presented in **Table 1**.

**Table 1. Example quotes from AGYW during formative in-depth interviews**	
**Perceptions about HIVST**	*"I think (HIVST) it's good since you become aware of your (HIV) status. It's not a must you go to the hospital if you're afraid. If you have sex with someone and you fear going to the hospital, you need to test yourself" (PrEP user, age 21)*
**Perceptions about PrEP**	*They (my peers) feel like they'll be stigmatized (for using PrEP), you know, mostly it is said that PrEP is majorly used by infected people. They haven't realized the difference between PrEP and HIV drugs" (PrEP user, age 20)*
**Peer PrEP referral + HIVST model**	*"I told them (peers) about PrEP…I tell them it doesn't have side effects, it is just like any other drug. When we meet, we just talk about it, I don't fear" (PrEP user, age 22)*

<u>Findings from stakeholder meeting peer referral to PrEP + HIVST</u>

On May 28, 2021 we convened a stakeholders' meeting with 34 participants from regulatory agencies and suppliers (e.g., Ministry of Health-, Pharmacy and Poisons Board, National AIDS & STI Control Programme, National AIDS Control Council), PrEP implementing partners (JHPIEGO, Clinton Health Access Initiative, Elton John AIDS Foundation), non-governmental organizations (e.g., LVCT Health, Bar Hostess Empowerment & Support Programme), health care providers (e.g., Kenyatta National Hospital, Ruiru Hospital), research institutions (e.g., Partners in Health & Research Development, Kenya Medical Research Institute, Jomo Kenyatta University of Agriculture and Technology), and young Kenyan women.

At this meeting, we first presented formative research findings on the acceptability and feasibility of the peer PrEP referral + HIVST delivery model among young Kenyan women to stakeholders. We then used the nominal group technique (NGT) to build consensus among stakeholders on the implementation strategies for the core components of this model to be including for pilot testing. The core components for which implementation strategies were developed included: 1) recruitment of PrEP users, 2) training of PrEP users for intervention delivery, 3) identification of peers at HIV risk, 4) delivery of HIVST and PrEP referral, 5) linkage to PrEP or HIV care support, and 6) PrEP adherence support (see **Fig. 1**). For the NGT, meeting participants first individually brainstormed implementation strategies for each core component that they then refined in small and large group discussions. Once the top implementation strategies for each component identified, individuals voted on the efficacy and feasibility of each strategy using 5-point Likert scale.

Fig. 1. Core components of the peer PrEP referral + HIVST delivery model for PrEP initiation among young Kenyan women

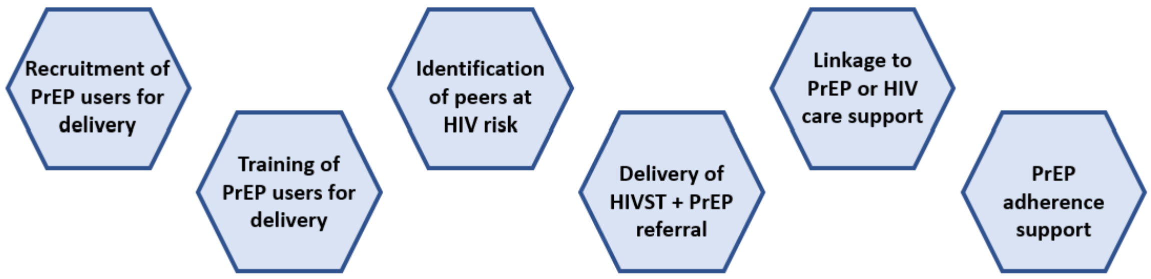

Overall, stakeholders were supportive of the peer PrEP referral + HIVST delivery model and felt that it would be acceptable and feasible among young Kenyan women. The highest scored strategies (by efficacy and feasibility ratings) were reviewed by stakeholders in the follow-up meeting (July $9^{th,}$ 2021) and incorporated into care pathway for pilot testing, summarized in **Fig. 2.**

Fig. 2. Collaboratively developed care pathway for peer PrEP referral + HIVST delivery for young Kenyan women

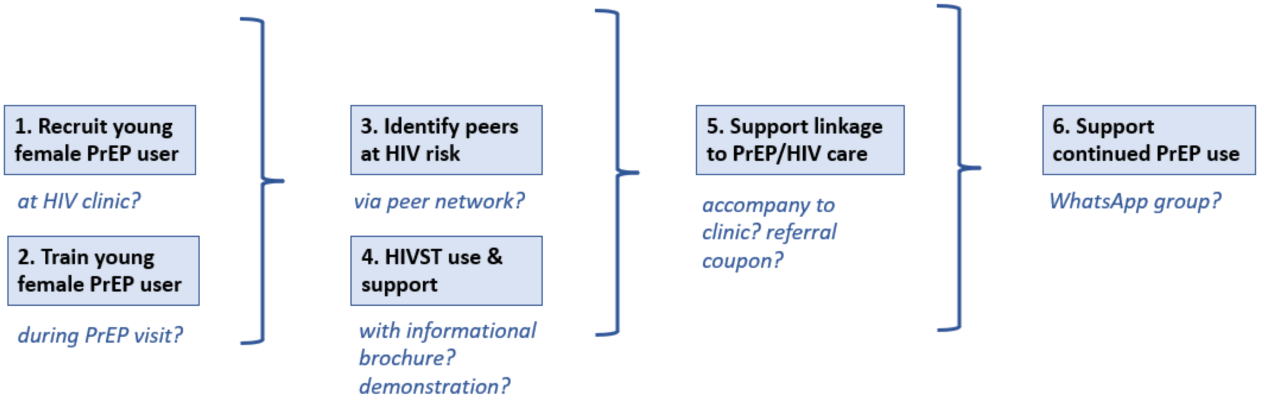

METHODS

Taking PrEP to scale will require simplifying and diversifying models for delivery to achieve options that are affordable, accessible, and meet the needs of AGYW and health systems. We have assembled a multidisciplinary team to test peer PrEP referral + HIVST delivery. **We hypothesize that a formalized peer referral model to PrEP will result in higher PrEP adoption (e.g., initiation and retention) among AGYW compared to on-going informal referral models.**

Study objectives

1. To test and refine a peer PrEP referral + HIVST delivery model (Aim 1 pilot)
2. To determine adoption and feasibility of a peer PrEP referral + HIVST delivery model (Aim 1 pilot)
3. To identify weak points for peer a PrEP referral + HIVST delivery model (Aim 1 pilot)
4. To test a model of PrEP initiation through an implementation randomized trial and to analyze study PrEP adoption, fidelity, cost, and safety (Aim 2 randomized trial)

Hypothesis. AGYW will be interested in a model of peer PrEP referral + HIVST. Understanding the potential weak points for peer PrEP referral will permit refinement of the intervention. Peer PrEP referral + HIVST will result in higher PrEP adoption (e.g., initiation and retention) compared to ongoing informal referral models.

Research questions

1. Will a peer PrEP referral + HIVST delivery model be adopted by and feasible among Kenyan AGYW?
2. What are the weak points in a peer PrEP referral + HIVST delivery model for PrEP initiation?
3. How can these weak points be mitigated for a future peer PrEP referral + HIVST delivery community-randomization trial?
4. How will a peer PrEP referral + HIVST delivery model compare in outcomes of PrEP adoption, fidelity, cost, and safety compared to ongoing informal peer PrEP referrals (the current standard of care)?

Study design

For Aim 1 (Objectives 1-3), we will conduct a one-arm intervention trial, or pilot study. There is no comparison arm in this pilot. The intervention we are testing was developed from extensive formative research, including analysis of data from in-depth qualitative interviews with young female PrEP users and non-users and a meeting with stakeholders.

For Aim 2 (Objective 4), we will subsequently conduct a two-arm hybrid type 3 randomized trial, in which young female PrEP users are randomized to: 1) “formal peer PrEP referral + HIVST delivery model” – where after training, participants are encouraged to refer 4 peers using PrEP educational materials and HIVST (2 HIVST kits/peer = 8 HIVST kits total) vs. 2) “informal peer PrEP referral”- where participants are encouraged to refer 4 peers to PrEP through word-of-mouth (**Fig. 3**).

Fig. 3: Randomized trial design (2-arms) to test peer PrEP referral + HIVST delivery.

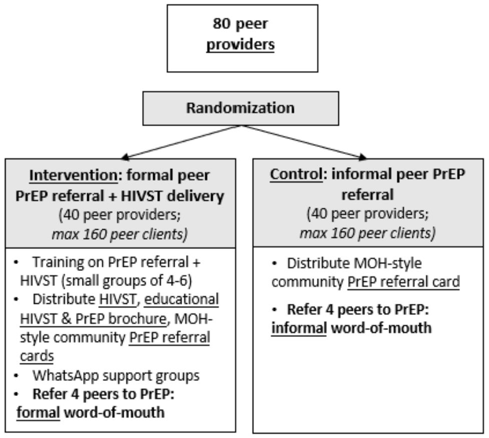

Setting

We will conduct the research for this study in Thika, Kenya. Thika is an urban center, ~40 km outside of Nairobi in Central Kenya, which has a large peri-urban and rural surrounding population. HIV prevalence in Thika is 6%.[3]

We selected Thika, Kenya to conduct this research because the research team implementing the project here has extensive experience with the provision of PrEP to diverse populations. They were involved in the Partners PrEP Study clinical trial,[8] then the open-label Partners Demonstration Project,[34] and are currently leading the Partners Scale-Up Project, MPYA study, POWER project, and pharmacy PrEP scale-up project. The site has technical expertise related to PrEP, community engagement with diverse populations (with high recruitment and retention >90%), and collaborative experience working with health providers outside of their own research clinics – precisely the components necessary for this work. The Thika research team also has extensive experience with the development of PrEP delivery models in Kenya – members of our study teams were deeply involved in the Kenya PrEP guideline process, including guidelines for clinical delivery.[35]

Aim 1: Pilot study procedures

We have chosen a single-arm trial for the pilot study (i.e., no comparison to an informal peer PrEP referral arm) because this study is focused on testing the adoption and feasibility of a formal peer PrEP referral + HIVST delivery intervention and refining care pathways for this delivery model. Ongoing informal PrEP referral can still be observed and can serve as a general comparison in terms of demographics, PrEP uptake, and adherence.

Participant eligibility & recruitment

We will recruit 16 AGYW using PrEP for the pilot study. PrEP users will be identified from prior Partners in Health and Research Development (PHRD) studies targeting AGYW and from public HIV clinics providing PrEP in Kiambu County. This will be a rolling recruitment and will end when all 16 participants have been identified. PrEP user participants will then be encouraged to refer 4 peers to PrEP, for a total of 64 peer study participants. The PrEP users will be trained on the importance of recruiting peers that are emancipated/mature minors only likely to benefit from using PrEP. This study sample size is deemed appropriate to measure the outcomes of interest and to refine the intervention care pathway prior to the

randomized trial. This sample size is also similar in size to other PrEP implementation pilots led by the PHRD (**Table 2**). We will use the same recruitment strategies for both emancipated minors 16-17 years and women ≥18 years.[36]

Table 2. Eligibility and recruitment strategies- pilot study

	Eligibility	Ineligibility	Recruitment Strategies
**PrEP users** (n=16)	• ≥16- to 24 years old[36]<br>• Female<br>• Adherent to PrEP (i.e., self-report taking PrEP all the time)<br>• Can identify 4 peers at HIV risk who would be interested in PrEP<br>• Able & willing to provide written informed consent	• <16 years old, >24 years old<br>• Male<br>• Not adherent to PrEP<br>• Cannot identify 4 peers who would be at HIV risk and/or interested in PrEP<br>• Is not able or willing to provide written informed consent	• Recruit participants from prior AGYW studies<br>• Recruit participants from HIV clinics where PrEP is available using PHRD recruitment strategies (e.g., workshops for HCWs)<br>• Strategies developed from the formative research and stakeholder meetings
**Peers** (n=64)	• Age ≥16 to 24 years*<br>• Female<br>• Referred by peer (i.e., a PrEP users) to initiate PrEP<br>• Able & willing to provide informed consent	• Age <16 or >24 years<br>• Male<br>• Not referred by peer (i.e., a PrEP users) to initiate PrEP<br>• Not able and willing to provide informed consent	• Peer referral from PrEP users

Pilot procedures

All participants will be enrolled in the pilot study and followed for 1 month, as outlined in **Fig. 4.**

Fig. 4. Timeline of pilot enrollment and follow-up

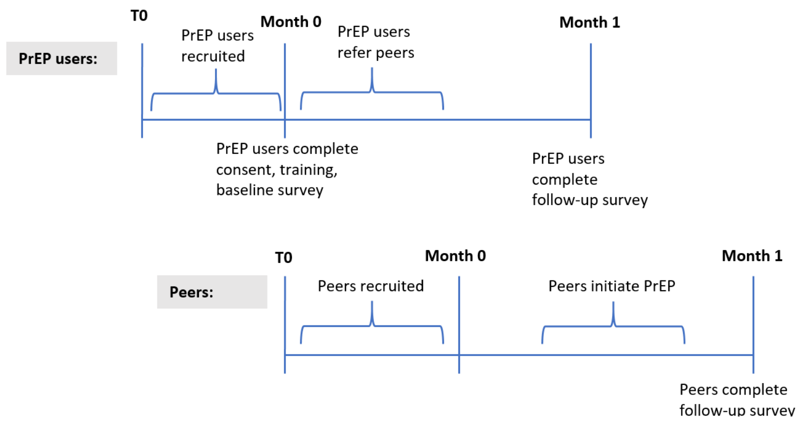

PrEP users. Once all PrEP users have been recruited and identified, they will complete a 1-day in-person group training (with appropriate COVID precautions in place) on the peer PrEP referral + delivery model (Month 0). This training will include education on HIV risk screening, HIV prevention, HIVST use (including results interpretation), PrEP adherence and safety, and the importance of confidentiality. With reference to the Rapid Assessment Screening Tool for PrEP (RAST), PrEP users will be taken through the eligibility criteria or risk factors that would warrant individual peers qualify to be initiated on PrEP. All PrEP users will be given educational resources on PrEP and HIVST (approved by the trial IRB) that they can share with their peers, including a list of locations in Thika where PrEP is provided for free (by Kenyan Ministry of Health [MoH]). Additionally, all PrEP users will receive 8 HIVST kits and be encouraged to use these (2 HIVST kits per peer) to refer 4 peers to PrEP. We will complete all enrollment procedures with PrEP users at the beginning of the group training, and all PrEP users will complete the quantitative baseline survey before training and complete a short post-training questionnaire to assess understanding of key components of HIVST and PrEP at the end of the group training. We will follow-up with all PrEP users in-person or remotely 1 month following this training to complete a structured questionnaire and collect self-reported outcomes, including the number of peers referred and PrEP initiation among peers (see Data analysis & outcomes section). During the COVID-19 pandemic, the Kenyan research team has successfully completed a number of remote questionnaires and in-depth interviews for other PrEP implementation and COVID-19 studies.

Peers. PrEP users will refer peers to the study using a recruitment script given to them at the training. The peers that are interested in HIVST, PrEP for HIV prevention, and study participation will call study staff (study team number specified in the recruitment script) before receiving the intervention from PrEP users so the study team has their contact information and can reach them 1 month later to complete a follow-up questionnaire. PrEP users will then deliver PrEP promotional materials and HIV self-tests to interested peers. The information of study staff and locations where peers can go to access free PrEP and HIV treatment services will be included in the promotional materials so peers can contact the study staff if they have any questions about PrEP, HIVST, or linkage to care. For peer follow up, research assistants will call peers 1 month following when they received the intervention from PrEP users (and gave their contact information to study staff) to complete a formal consent process and a structured quantitative questionnaire either remotely or in-person (at the research site or at their home).

All participants will receive transportation reimbursement and an additional 300 KES (~$2.70 USD) to compensate for their time and effort and for completion of a structured questionnaire at baseline (PrEP users) or follow-up (PrEP users and peers). PrEP users will also receive an additional small incentive for participation in training and peer recruitment activities. For participants that have to complete questionnaires remotely, we will send mobile money to reimburse participants.

Intervention core components

Throughout the duration of the study, participants will support the implementation of the peer PrEP referral + HIVST model through core components identified in the stakeholder meetings (**Fig. 1**).

1. Recruit young female PrEP users – through health facilities and institutions. Specifically, study counsellors and healthcare workers (HCWs) will identify young women who can serve as "PrEP champions" (i.e., display knowledge about PrEP, engage in PrEP adherence, and have the ability to recruit/train peers).

2. Train young female PrEP users – through a 1-day in-person group or individualized training using a targeted curriculum. It is vital that training is appropriate to the AGYW age group (e.g., information is comprehensible, engaging). Further, this approach may be combined with elements of peer education, an approach which had similar ratings in terms of effectiveness and feasibility. Peer education is deemed appropriate to educate AGYW in terms of HIVST use and interpretation as well as PrEP use, efficacy, and side effects.

3. Identify peers at HIV risk – through peer networks. Participants will identify peers through snowballing methods and will approach the topic of PrEP by demonstrating emotional support to peers (e.g., sharing personal experiences using PrEP).

4. HIVST use and support – through the in-person or online provision of information, education, and communication (IEC) materials. These materials may include posters, graphics, package inserts (HIVST kits) or pamphlets to aid researchers and healthcare workers teaching AGYW about HIVST and PrEP.

5. Support linkage to PrEP or HIV care – through information provided in the PrEP pamphlet shared with them by PrEP users, which includes the contact information of study staff that can help support linkage to care and the information for nearby health facilities that provide free PrEP services. Additionally, PrEP users will be trained on how to guide peers on linkage to nearby free PrEP services and may escort peers to these services if peers request this and they feel comfortable doing so. PrEP users who support their peers via escorted referrals will be asked to encourage peers to return for PrEP refills without escorted referrals to mitigate peer dependency.

6. Support continued PrEP use – through the implementation of peer support groups and drug-buddies. Drug-buddies refer to peers who are paired and can hold one another accountable to daily PrEP use. PrEP adherence will also be combined with a formalized PrEP reminder and tracking system (e.g., USHAURI phone application). Further, continued PrEP use will be supported through the implementation of capacity building activities among healthcare workers to provide youth friendly PrEP services.

The PrEP medication will be provided for free from the public health clinics via the Kenyan MoH and HIV self-tests for this pilot study will be provided for free via ChemBio Diagnostics Systems.

This pilot study will be conducted during the ongoing COVID-19 pandemic. As such, the pilot study will be conducted in accordance with the Kenyan COVID-19 prevention measures in place at the time of the study. Participants will be asked/screened about symptoms of COVID-19 (including temperature) prior to entering the PHRD study site for a follow-up appointment, all in-person data collection will be conducted with appropriate distance, ventilation, and participants will be asked to wear a face mask to reduce the risk of COVID-19 infection. As necessary, we will explore options to complete surveys over the phone.

Condoms will be available through the PHRD research site but are not included in the package of PrEP services provided in this study.

PrEP medication

Tenofovir disoproxil fumarate (or TDF, 9-[(R)-2-[[bis [[(isopropoxycarbonyl) oxy] methoxy] phosphinyl] methoxy] propyl] adenine fumarate), emtricitabine (or FTC, 5-fluoro-1-(2R,5S)-[2-(hydroxymethyl)-1,3-oxatholan-5-yl]cytosine), and lamivudine (or 3TC, 2',3'-dideoxy-3'-thiacytidine 4-Amino- 1-[(2R,5S)- 2-(hydroxymethyl)- 1,3-oxathiolan-5-yl]- 1,2-dihydropyrimidin- 2-one) are reverse transcriptase inhibitors that have been approved for the treatment of HIV infection in humans in Kenya and the United States. A fixed-dose, oral co-formulation of FTC/TDF (Truvada) has also been approved for HIV prevention in Kenya and the United States. The WHO recommends TDF-containing medications as PrEP, which includes TDF combined with FTC as well as potentially TDF alone and TDF combined with lamivudine (or 3TC, a medication closely related to FTC). Any TDF-containing medications that align with WHO and Kenyan national guidelines for PrEP will be used in this study. PrEP will be prescribed for once-daily oral use. Study medication will be provided by the Kenyan MoH and stored in accordance with the drug manufacturer's recommendations.

PrEP discontinuation

PrEP initiation and continuation will be in accordance with the Kenyan PrEP guidelines. Use of PrEP may be interrupted by the site investigators or Kenyan clinicians due to safety concerns for the participant or use of concomitant medications that could interfere with PrEP or present a safety concern. All treatment interruptions will be documented.

Referral to continued PrEP care

For participants that wish to continue PrEP use following the pilot study (1-month trial duration); participants will remain enrolled in PrEP at the healthcare facility at which they had enrolled/initiated in PrEP use. PrEP will then be prescribed and administered via this physician who will provide 3-month supplies of PrEP.

HIV Self-Testing Kit

ChemBio (who has committed to providing HIVST kits to the pilot study) manufactures the SURE CHECK

HIV 1/2 Assay, a blood-based HIVST that has 99.7% sensitivity, 99.9% specificity, presents results in 15 minutes, and is approved by the US FDA.[37] Results are easy to read (1 line: HIV-negative, 2 lines: HIV-positive, no lines: invalid result). HIVST serves as a preliminary test result and must be followed-up with confirmatory facility-based HIV testing to indicate a diagnosis.

Data Collection

Quantitative data will be collected through surveys at baseline (Month 0) and follow-up (Month 1) and focus group discussions (FGDs) will be completed near the end of the pilot (Month 3).

Quantitative surveys. Trained research assistants (RAs) stationed at healthcare facilities delivering PrEP will approach eligible individuals and invite them to participate in the study. They will review the informed consent form with these individuals and answer any questions they may have regarding participation. Once written informed consent is obtained, RAs will conduct a structured quantitative survey with the participant. **Table 3.** outlines the data collection activities which will be conducted among the participant (PrEP user) and the recruited peers at baseline (Month 0) and follow-up (Month 1). All quantitative data will be collected on a tablet using CommCare (Dimagi, Cambridge, USA), an electronic data collection platform.

Table 3. Data collection activities

Variables	PrEP user M0	PrEP user M1	Peer M1
Demographics	X		X
HIV risk	X		X
Depression[38,39]	X		X
Sexual health/HPV	X		X
Self-efficacy[40]	X		X
Peer support	X		X
HIVST distribute/use		X	X
PrEP initiation/use	X	X	X
PrEP/HIV stigma	X	X	X

FGDs. We will conduct FGDs with about 12 PrEP users who delivered and about 12 peers who received the peer PrEP referral + HIVST delivery model. We will conduct 4-5 FGDs in total (3-6 participant/FGD); of these 2-3 FGDs will be conducted among PrEP users (recruiters) and 2-3 FGDs will be conducted among peers (those recruited to the study). We hereby aim to explore their experiences with this new PrEP delivery model and understand the acceptability of a peer PrEP referral + HIVST model. FGDs will utilize the Consolidated Framework for Implementation Research (CFIR)[41] to understand model acceptability, measure model weak points (barriers) and to assess model facilitators in order to inform the subsequent randomization trial. Topics discussed in the FGDs are summarized in **Table 4**. All participants that participate in these focus group discussions will sign documents of informed consent. Additionally, all participants will be reimbursed for transportation and an additional 300 KES (~$2.70 USD) for participation in FGDs.

Table 4. FGD themes, informed by CFIR domains: constructs

Intervention: complexity	Clarity of instructions/logistics
Outer: peer pressure	Peer influence on HIVST use, PrEP initiation
Individual: self-efficacy	Confidence to initiate PrEP, use HIVST
Individual: knowledge	Understanding of PrEP benefits, HIV risk
Process; Executing	Experiences delivering model/materials
Process: Reflecting	Attitudes towards the model, identify weak points

All qualitative FGDs will be conducted in the participant's preferred language (English or Kiswahili) by trained qualitative research assistants using pre-piloted, semi-structured interview guides. Each FGD will be audio recorded, transcribed, and translated into English by study team members who will also routinely complete debriefing reports[42] to accelerate real time learning. Dr. Ngure, an experienced behavioral scientist who has led our qualitative research in Thika for the past decade, will supervise these discussions.

Data analysis & outcomes

Quantitative surveys. **Table 5** shows the study outcomes that will be obtained from the quantitative surveys. For Aim 1 (Objective 1), we will calculate the number and proportion of participants who initiated PrEP at healthcare facilities, peers referred to PrEP, and peers that used HIVST. These outcomes will

be measured along outcomes of “acceptability,” “appropriateness,” and “feasibility” (Objective 2).

**Table 5. Outcomes for the peer PrEP referral + HIVST delivery pilot and measured at Month 1.** * primary			**Metric of success:** Allow seamless progression to trial
**Outcome:**	**Details:**	**Reported:**	
PrEP initiation*	Prop. of referred peers that initiated PrEP*	Peers and PrEP users	40% of those followed up (n≥19)
PrEP referral	No. of peers referred to PrEP	PrEP users	≥3 peers per PrEP user (n≥48)
HIVST use	Prop. of referred peers that used HIVST	Peers	75% of those followed up (n≥36)

FGDs. Transcripts will be reviewed separately by two or more qualitative researchers, who will ensure completeness. These researchers will immerse themselves in the data through repeated readings of the transcripts and create a preliminary codebook of inductive[43,44] and deductive[45–47] codes to capture the experiences of AGYW regarding a peer PrEP referral + HIVST model including barriers and facilitators (Objective 3). A sample of transcripts will be double coded independently by two or more researchers, with coding discrepancies identified and resolved via consensus. During this process, the codebook will be refined, with existing codes combined, separated, or eliminated and new codes added as needed to capture emerging themes.[48,49] Thereafter, remaining transcripts will be coded in Dedoose (Los Angeles, California, USA) or Nvivo 12 (QSR International, Melbourne, Australia). We have extensive experience analyzing qualitative data to inform intervention development.[50–72]

Participant retention and withdrawal

The Thika research site will develop retention methods tailored to and most efficient for the local study settings. Retention activities may include explanation of the study schedule (i.e., return for follow-up after one month) and procedural requirements during the informed consent process. Further, peers referred to the study, will be contacted and directed to the PHRD study site for follow-up or will be visited (e.g., at home) by PHRD researchers. However, should the aimed sample size not be attained (e.g., <75% referral rate), then alternative follow-up strategies may be implemented including 1) giving peers mobile money conditional on texting researchers their contact information or 2) having PrEP users report outcomes for their peers.

Participants may voluntarily withdraw from the study for any reason at any time. The site investigators may also withdraw participants from the study in order to protect their safety and/or if they are unwilling or unable to comply with required study procedures. Reasons for withdrawal will be recorded.

Limitations

This pilot study has some potential limitations that are important to note. First, some of the participants may be recruited for this study from prior or ongoing PHRD studies targeting AGYW. These participants may not accurately represent PrEP and HIVST knowledge among AGYW who have not been enrolled in prior PrEP and HIVST interventions. We aim to mitigate this challenge by recruiting other participants from public HIV clinics who have no prior PrEP and/or HIVST education.

Additionally, the pilot study will encourage participants to recruit 4 peers to the study. The limitation of 4 peers may inherently exclude some AGYW who maintain at high risk of HIV and would be interested in PrEP initiation through a peer PrEP referral+ HIVST model. Further, those recruited to the pilot study will be provided 2 HIVST kits to test themselves (independently or with the assistance from the PrEP user) or to test a primary sexual partner. Some AGYW may benefit from testing multiple sex partners (e.g., in sex worker relationships). Although a focus on partner testing is outside the scope of this study- future research should be considered in this area.

Finally, in the pilot study, peers recruited to the study are supplied HIVST kits free of cost (from ChemBio).

In settings outside of a research or intervention setting, AGYW may have to purchase HIVST kits at cost which may influence the appropriateness and feasibility of this model. The randomization trial will further research this factor through a cost analysis.

Aim 2: Randomization trial procedures

Study population

Based on confusion with naming conventions from the pilot study, we have revised our description of PrEP users and peers to the following: "peer providers" and "peer clients." From here on out, peer providers (formerly "PrEP users") will be young women using PrEP who we will training on the peer PrEP referral + HIVST delivery in in the intervention arm or who will continue with informal models of peer PrEP referral in the standard-of-care control arm. Peer clients (formerly "peers") will then be the young women that receive the peer PrEP referral + HIVST intervention delivered by the peer providers. For reporting purposes, we will also refer to peer providers as "index peers" and peer clients as "referred peers."

We will recruit 80 young female peer providers from public healthcare clinics delivering PrEP in Kiambu County and neighboring Counties using established PHRD site techniques. These participants will further refer 4 peer clients each to PrEP for a maximum 320 potential peer clients.

Eligibility criteria.

Peer providers. Eligible peer providers will be: i) female, ii) ≥16 to 24 years old (including emancipated minors ≥16 to 17 years[36]), iii) must have refilled or initiated PrEP (i.e., been dispensed PrEP), iv) are able to identify 4 peers at risk of HIV infection who may be interested in PrEP, v) not currently enrolled in another HIV study, and vi) are able and willing to be randomized to the intervention, complete research activities, and provide written consent (**Table 6**). Due to the nature of the training intervention which includes slides, quizzes, and other activities that involve reading and writing, peer providers will be considered ineligible if they do not have basic literacy skills. Due to some challenges recruiting young female peer providers in the Aim 1 pilot, we made some modifications to the inclusion criteria in this section, including reducing the duration of PrEP use and dropping the requirement for PrEP disclosure.

Peer clients. Eligible peer clients will be: i) female, ii) ≥16 to 24 years old (including emancipated minors ≥16 to 17 years[36]), iii) referred by a peer provider to initiate PrEP, and iv) are able and willing to provide informed consent.

Recruitment strategies. *Peer providers.* Peer providers will be recruited through a multipronged approach that will include recruitment through other ongoing PrEP research projects, and from nearby public health facilities implementing PrEP programs, such as, workshops for healthcare workers, use of IEC materials, and working with community health workers to identify potential participants. Peer providers will be compensated 300 KES (~$2.70 USD) and be reimbursed for travel at their baseline appointment and again at their 3-month follow-up appointment. No travel reimbursement at follow-up will be provided if follow-up questionnaires are conducted via phone. All peer providers will be encouraged to disclose PrEP use to peers, although this is not required. Study counselors will conduct follow-up calls with peer providers to check in and see how the referral process is going and answer any questions or concerns regarding the intervention. Recruitment of peer providers will be on a rolling basis, so it is possible that peer providers and peer clients may not be mutually exclusive (as future peer providers could be previous peer clients).
Peer clients. Peer providers will recruit peer clients. Peer clients will contact study counselors (via phone), to provide RAs with contact information to schedule a follow-up visit (Month 3) at the PHRD study site (transport refunded) or via phone interview. Clients won't be engaged in study activities at Month 3 until they have provided informed consent. To help prompt peer providers to remind peer clients to call

research staff at the point of recruitment (a challenge during the pilot study), we will compensate peer providers and their peer clients a small amount (150 KES each, ~$1.40 USD each) each time a peer client pings the study phone line to enroll in the study. For any peer clients who do not ping the study phone, study staff will attempt to collect contact information for the peer clients from the peer providers during a follow-up visit/call. The peer client can still decline enrollment when contacted by study staff. Peer clients will be compensated 300 KES (~$2.70 USD) and reimbursed for travel for attending the 3-month follow-up appointment. No travel reimbursement will be provided if the questionnaires are conducted over the phone. To facilitate the delivery of this compensation, we will use a secure mobile money platform (M-Pesa) that we have used successfully for other research projects. Research staff will call these individuals twice (one week apart) and stop trying to contact these potential participants if these phone calls do not go through. Additionally, they will do their best to confirm the identity of the potential peer client before describing any details of the research study (and their potential participation in this study).

Consenting procedures. *Peer providers.* All peer providers that meet eligibility requirements will provide written informed consent at the point of recruitment and prior to randomization with a trained research assistant. This signed consent form will be stored as confirmation of informed consent and a copy will be made available to the participant.

Peer clients. All peer clients that have contacted the study team directly, expressed interest in the study, and have met eligibility requirements will complete informed consent at the Month 3 follow-up visit, before completing any questionnaires assessing their experience with the peer PrEP referral + HIVST intervention or informal peer PrEP referral process. Those unable or unwilling to come to the PHRD site to sign a consent form will have the option to provide verbal consent over the phone. If verbal consent was provided, the trained research assistant will note the date that verbal consent was obtained on a copy of the consent script. The signed consent form and scripts will be stored as confirmation of informed consent. A subset of peer clients who sign the written consent form will also be asked to provide a DBS sample collected from a finger prick or venous blood. No peer client providing verbal consent will be asked to provide a DBS sample.

Table 6. Eligibility and recruitment strategies- randomized trial			
	**Eligibility**	**Ineligibility**	**Recruitment Strategies**
**Peer providers** *(formerly "PrEP users")* (n=80)	• ≥16- 24 years old[36]<br>• Female<br>• Must have refilled or initiated PrEP (i.e., been dispensed PrEP)<br>• Not currently enrolled in an HIV study<br>• Can identify 4 peers at HIV risk who could benefit from PrEP<br>• Able & willing to be randomized to the intervention, participate in research activities, and/or provide written informed consent	• Not between 16-24 years old<br>• Male<br>• Have not used PrEP<br>• Cannot identify 4 peers who would benefit from PrEP<br>• Is not able or willing to be randomized to the intervention, participate in research activities, and/or provide written informed consent<br>• Currently enrolled in an HIV study<br>• Illiterate	• Recruit participants from HIV clinics where PrEP is available using PHRD recruitment strategies (e.g., workshops for healthcare workers)<br>• Strategies developed and refined during the pilot study
**Peer clients** (formerly "peers") (n=320)	• Age ≥16 to 24 years<br>• Female<br>• Referred by peer provider (i.e., a "PrEP user") to initiate PrEP<br>• Able & willing to provide informed consent	• Age <16 or >24 years<br>• Male<br>• Not referred by peer provider (i.e., a PrEP users) to initiate PrEP<br>• Not able and willing to provide informed consent	• Peer providers will recruit peer clients. At point the of recruitment, peer clients will call research staff to provide contact info for follow-up<br>• At follow-up, peer providers share the contact information of peer clients so they can be reached for follow-up.<br>• If peer client calls the study staff, both the peer client and provider will be compensated 150 KES via mobile money.

Randomization

Shortly after trial enrollment, peer providers will open in the presence of a study RA an opaque sealed envelope sequentially distributed to them that has their randomization arm specified inside (e.g., formal peer PrEP referral + HIVST arm; informal peer PrEP referral arm).

Study procedures & intervention

Depending on the arm peer providers are randomized to, the following procedures will apply (**Table 7 and Fig. 5**):

Table 7. Interventions/materials received by peer providers and peer clients in the intervention and control arms				
**Intervention/material**	**Intervention arm**		**Control arm**	
	Peer provider	***Peer client***	***Peer provider***	***Peer client***
Formal 1-day training on PrEP & HVIST	X			
Formalized conversation about PrEP, HIVST & referral	X	X		
PrEP educational brochure	X	X		
HIVST kits for distribution/self-testing	X	X		
WhatsApp group for questions	X			
PrEP referral card	X	X	X	X
Informal conversation about PrEP & referral			X	X

[Intervention arm]: Formal peer PrEP referral + HIVST delivery (n=40 peer providers). Peer providers randomized to this intervention arm will receive a training on PrEP referral and HIVST delivery. We will train peer providers in groups of around 4-8 peer providers. The training materials will be based on Kenya's national PrEP guidelines and delivered in a 1-day session led by experienced research staff. The training will cover HIV prevention methods, how to use and interpret HIVST kits, how to initiate conversation with peers about PrEP, practice scenarios, pre-training and post-training quizzes, and other related activities. Based on feedback from the Objective 2 FGDs, we modified the pilot training materials

to also include more training on HIVST interpretation, common PrEP misconceptions, issues surrounding PrEP stigma, reminders for consistent PrEP use, and the importance of delivering the intervention package to peers privately.

Following the training, peer providers will be asked to identify 4 peer clients they perceive as at HIV risk and think might be interested in daily oral PrEP and deliver the intervention to these individuals. This intervention arm will include a formalized conversation about PrEP with peers, delivery of HIVST kits, sharing a PrEP & HIVST educational brochure, delivery of a PrEP referral card, and access to a (monitored) WhatsApp group to ask questions and for support **(Table 7 and Fig. 5)**:

- *Formalized PrEP conversation.* Peer provider in this intervention arm will be trained on techniques they can use to start conversations with their peer clients about PrEP and provide correct information about PrEP effectiveness, use, and safety (and answer any questions related to these). Additionally, they will deliver a study-developed PrEP and HIVST informational brochure to peer clients that covers the talking points about PrEP use and safety and can be used by as a reference in the future.
- *HIVST kit delivery.* The intervention will include the distribution of 2 HIVST kits by peer providers to each peer client. With delivery of the HIVST kits, peer providers will be trained to share information on how to use the HIVST kits with peer clients and direct them to the information and pictorial brochure that comes with the HIVST kits for more information as needed. Referred peer clients can use the HIVST kits to test themselves for HIV, test for HIV with a peer, or test a main sexual partner (if they feel comfortable) which may increase feelings of self-efficacy and knowledge of PrEP eligibility or need – potentially facilitating PrEP initiation.
- *PrEP educational brochure:* This informational brochure will reinforce key points of the training session provided to peer providers. It will be delivered by peer providers to peer clients during the formalized PrEP conversation.
- *PrEP referral card.* The intervention will also include the delivery of a PrEP referral card by peer provider to peer clients with training on how to help peers link to PrEP services if needed. On this card will be listed information on the location of nearby public clinics and in neighboring counties with free PrEP services and that peer client can ask for when they arrive for assistance with PrEP services. This referral card will also contain the study phone number to support peer clients that wish to go to another facility for PrEP support.
- *WhatsApp Group.* The study team will answer questions, provide additional information, and promote discussion between peer providers through a monitored WhatsApp group (participation is optional). This forum has been used in other studies at PHRD and we plan to implement several measures to make these groups as confidential as possible. First, individual WhatsApp groups will be created for each peer provider training session. That way, peer providers that participate in this option will only be conversing with individuals they met during their training session. Second, during the training session, peer providers will be instructed to not disclose any identifying information about peer clients in the WhatsApp group. Third, a study team member will be assigned to monitor the group 24/7. If necessary, study staff will delete any messages that contain personal information about participants immediately. The study staff monitoring the group will also periodically delete older messages, so they are not stored long-term. Finally, the WhatsApp groups will only be available during the 3 month intervention time period and then all remaining messages from that group will be permanently deleted.

Peer providers randomized to this arm will be encouraged to disclose PrEP use to peers, although this is not required.

[Control arm]: Informal peer PrEP referral (n=40 peer providers). Peer providers randomized to this

control arm will be encouraged to refer 4 peers that they perceive to be at HIV risk and would be interested in daily oral PrEP through free public PrEP services. This control arm will include informal conversations about PrEP with peers (as per the standard of care) and delivery of a PrEP referral card **(Table 7 and Fig. 5)**:

- *Informal PrEP conversations.* Peer providers in this arm will not receive any formalized training on PrEP or HIVST and will not receive any HIVST kits for distribution to peers. They will be encouraged to talk with their peers about PrEP in the way that feels most comfortable to them and in way they would typically have conversations about new products (e.g., lipstick) or things that interest them.
- *PrEP referral card.* This control arm will also include the delivery of a PrEP referral card by peer providers to peer clients. This will be the same referral card as that delivered to peer clients in the intervention arm, with information of nearby public clinics with free PrEP services. This referral card will also contain the study phone number so peer clients remember to ping the study counselor when these informal conversations occur and to support peer clients that wish to go to another facility for PrEP support.

Like in the intervention arm, peer providers randomized to this arm will be encouraged to disclose PrEP use to peers, although this is not required. No WhatsApp group will be available in this arm.

Fig. 5. Study procedures for intervention, and standard of care groups.

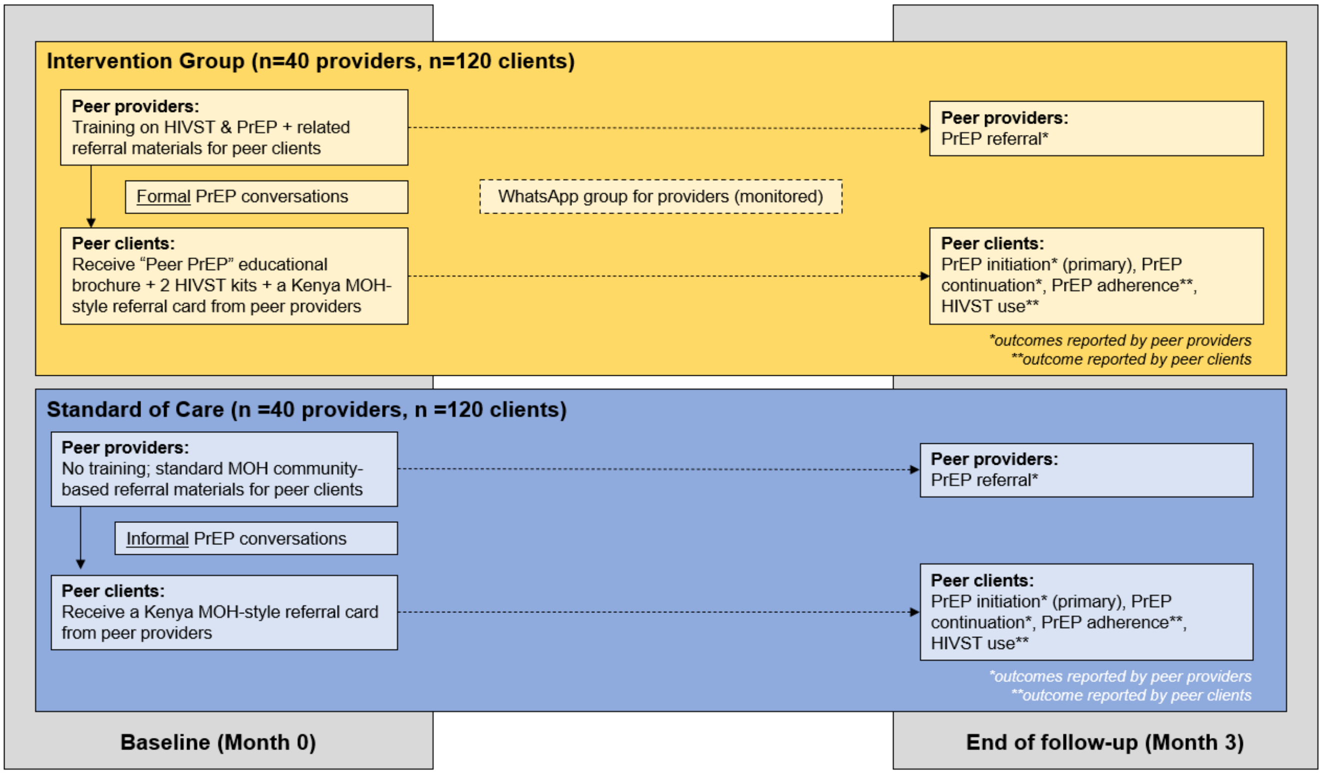

Data Collection

Fig. 6. Timeline for the Aim 3 randomized trial.

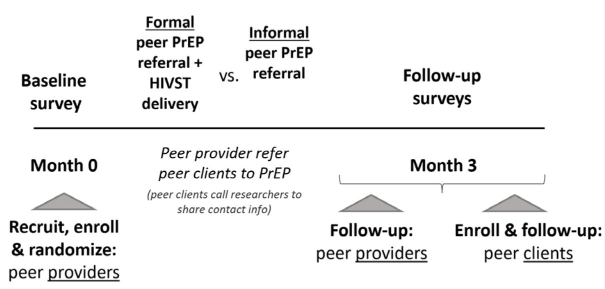

Overview. **Fig. 6** highlights a timeline of study visits and data collection activities for this trial. There are two data collection time points in this study: Month 0 (peer provider baseline questionnaire) and at Month 3 (peer provider follow-up questionnaire and peer client follow-up questionnaire). After providing informed consent, all peer providers enrolled in the trial will complete a baseline (Month 0) and follow-up (Month 3) survey. Additionally, all peer clients who voluntarily provide their contact information directly to the study team and request to be enrolled in the study will be contacted to schedule an interview at Month 3. The peer client will be screened for eligibility and consented prior to completing a follow-up survey at Month 3. This timeline will provide recruited peer clients with the opportunity to enroll in PrEP (approx. 1 month following the baseline survey) and return to healthcare facilities for one PrEP refill (approx. 3 months following the baseline survey). Sociodemographic information will be collected at baseline among peer providers and follow-up among peer clients (i.e., their first research visit). At all follow-up visits, we will measure both self-reported outcomes related to adoption of PrEP services, as well as process-level outcomes (e.g., number of HIVST kits received in the intervention arm, PrEP referral cards received in both arms), and implementation outcomes (e.g., intervention acceptability, appropriateness, fidelity, satisfaction).

Setting. Experienced quantitative researchers will collect electronic data using CommCare (Dimagi, Cambridge, USA), an electronic data collection tool, in questionnaires at baseline (Month 0) and follow-up (Month 3). While we will encourage all enrolled peer clients to come to the PHRD research site to complete the questionnaires in person, we will additionally allow for the option of questionnaire participation/completion over the phone to allow for easier engagement in follow-up activities (in particular, peer clients that might have been recruited outside of Kiambu County). Peer providers will need to come in-person to the clinic during baseline to receive the intervention they are randomized to. All participants will receive 300 KES (~$2.70 USD) for their time and effort completing each study visit/questionnaire. For participants completing questionnaires remotely via the phone, we will send this reimbursement via a secured mobile money platform (i.e., M-Pesa), as we have done routinely for other projects. For participants that come to the PHRD research site to complete these visits/questionnaires in person, we will additionally provide reimbursement for transportation.

DBS samples. At follow-up (Month 3) DBS samples will be collected from peer clients to validate PrEP initiation. At a subset of in-person visits, a DBS collected from a finger prick or venous blood will be prepared at the site and stored at -20 degrees Celsius. Prior to storage, the DBS will be left to air-dry for at least 2 hours and thereafter placed in low gas-permeability Ziploc bags with a desiccant pack to reduce humidity thus maintaining integrity of the sample. The DBS samples will be labeled with the de-identified participant ID and will be shipped outside Kenya for temporary storage at Fred Hutchinson Cancer Center (Washington State, USA) and forwarded to Molecular Testing Labs (Washington State, USA) for TFV and FTC level testing. The shipped samples will be stored until the testing is completed and results confirmed. These outcomes are expanded on in the following section. It is possible that DBS samples will not be sent for testing if we do not achieve the target sample size of 23 peer clients per study arm.

Data Analysis

All outcomes will be measured at study follow-up (Month 3) (**Table. 8**).

**Table 8. Outcomes for the peer PrEP referral + HIVST delivery trial, measured at 3 months.** * = primary					**Metrics of success:** for R01 progression
**Outcome:**	**Details:**	**Outcome type:**	**Reported:**	**Measures:**	
PrEP initiation*	Prop. of clients initiating PrEP	Adoption (clients)	Provider	No. of new clients using PrEP / No. of referred clients	>20% increase
PrEP referral	No. of clients referred to PrEP	Adoption process (providers)	Provider	No. of clients referred to PrEP / No. of referred clients	>50 more peers
PrEP continuation[1]	Prop. of clients refilling PrEP (any) since referral	Adoption secondary (clients)	Provider	No. of clients with >1 PrEP clinic visits / No. of referred clients	>20% increase
PrEP continuation[1]	Prop. of providers refilling PrEP (any) in the past 3 months	Adoption secondary (providers)	Provider	No. of providers with 1+ PrEP clinic visits in past three months / No. of providers	>20% increase
PrEP adherence	Median of Wilson et al's 0-100 point adherence score	Adoption secondary (clients)	Client	Median score	>20% increase
HIVST use	Prop. of clients in the formal arm that used a HIVST.	Adoption process (clients)	Client	No. of clients self-reporting use of HIVST / No. of referred clients	≥90% (n=144 – formal arm)
Client linkage to care	Prop. of clients that went to a clinic to access HIV services	Adoption process (users)	Client	No. of clients reporting visiting a clinic / No. of referred clients	>20% increase
Recent HIV testing	Prop. of clients who completed any HIV testing since referral	Adoption secondary (clients)	Provider	No. clients reporting testing for HIV since referral/ No. of referred clients	>20% increase

[1]Retention: >1 PrEP clinic visit (guidelines recommend first-time PrEP users return to a clinic after one month)

<u>Primary adoption outcome</u>. Our primary outcome is reported PrEP initiation among all referred peer clients, as reported by peer providers (Objective 4). We had originally planned to measure this as a self-reported outcome among peer clients but had a challenge reaching peer clients that were referred in the pilot study; thus, have decided to link measurement of our primary outcome to peer providers delivering the intervention, as is typical in many studies assessing the impact of secondary distribution delivery models. To validate this, we will collect a DBS sample from a subset of willing peer clients that return in person at Month 3 (approximately 23/arm) to measure concentrations of tenofovir diphosphate (TFV-DP) and emtricitabine triphosphate (FTC-TP) using a validated liquid chromatography tandem mass spectrometry[73,17]. Any concentrations of TFV-DP in a DBS sample will confirm PrEP initiation and/or adherence.[16] In sensitivity analyses, we will measure our primary outcome using self-report data from peer clients (not providers) on PrEP initiation.

<u>Secondary adoption outcomes</u>. We will also measure secondary and process outcomes of peer clients as reported by peer providers, including number of peers referred, recent HIV testing, and PrEP continuation (e.g., peer clients that returned to the clinic and refilled PrEP drugs one month following initiation). As in the primary analysis, we will measure these outcomes using reported data from peer clients (not providers) in sensitivity analyses. We will also measure some secondary and process outcomes of peer clients as reported by themselves, including PrEP adherence (measured using a standard scale), HIVST use, and linkage to care. Finally, we will also measure a secondary outcome of PrEP continuation among peer providers, as reported by themselves.

<u>Implementation outcomes</u>. Additionally, in all follow-up surveys among peer providers and clients we will measure a number of implementation outcomes including participants' perceptions of the intervention's

acceptability, feasibility, and appropriateness. To measure these outcomes, we will use validated scales and established theories/frameworks adapted to the Kenyan setting and to young women (e.g., the Theoretical Framework of Acceptability). Additionally, at follow-up, we will have peer clients complete a checklist of materials received from peer providers to measure "fidelity" of the intervention in both intervention arms.

Costs are another important implementation outcome we plan on measuring during this trial. To measure costs, we will micro-cost the formal delivery model and compare the incremental cost of this model with the existing informal referral strategy (Objective 4). Activity-based micro-costing will be conducted for costs incurred (e.g., HIVST kits) and costs averted (e.g., personnel). We will use time and motion studies to measure staff time spent training peer providers. Estimates of cost using the activity-based approach will be compared with the top-down approach (i.e., the budget divided by the number of peer providers and peer clients) [74] [75] [76] [77] (**Table 9**).

Safety. We will measure "safety" of the intervention by screening for PrEP side effects (i.e., nausea, vomiting, headache) among peer clients (and as reported by both peer provider and clients) and any reports of social harm (e.g., physical, verbal, emotional violence) related to our delivery model (Objective 4).

**Table 9. Description of costing inputs**
**Costs:** Start-up: microplanning, training, mobilization; Recurrent: personnel, capital, supplies, overhead
**Data:** Project budget; time & motion; staff interviews; published literature; central price list & salaries
**Unit cost:** Cost/PrEP users trained, peer referred, peer initiated on PrEP, peer retained on PrEP.

The findings from this trial (described above) will be used to inform a NIH R01 proposal for a peer PrEP referral + HIVST community-randomized trial and budget impact analysis.

Statistical analysis

We will measure effect size estimates using mixed effects multilevel regression models (primary predictor: study arm; standard errors adjusted for clustering by peer provider). All analyses comparing randomization arms will be intention-to-treat, complete case analyses conducted at the unit of the individual (either peer provider or peer client). The primary comparison will be formalized peer PrEP referral + HIVST delivery versus informal peer PrEP referral (the standard of care). For analyses conducted at the level of the peer client, we will adjust standard errors for clustering at the peer provider level. We will use mixed-effect logistic regression models to measure risk ratios. All statistical test with 2-sided p-values <0.05 will be considered statistically significant. We will complete all analyses in R and STATA.

Sample size

If we assume that peer providers refer 75% of the targeted peer clients, this leaves us with three clients per peer provider, or 240 peer clients in total. We performed power calculations for our primary outcome, the proportion of referred peer clients who initiated PrEP at three months, in Stata 16.1 using methods for cluster-randomized trials, where each cluster is a peer group of one peer provider and three peer clients **(Figure 7). Table 10** shows the power we have to attain to detect various increases in the proportion of peer clients who initiated PrEP in the formal peer PrEP referral arm + HIVST (intervention group) vs. the informal peer PrEP referral arm (standard of care group). For our power calculations, we assumed 40 clusters (peer providers) per arm, 3 sampling units (peer clients) per cluster, and an intra-cluster correlation coefficient (ICC) of 0.05, which is standard for cluster-randomized controlled trials.

Fig. 7. Power calculation to detect increase in the percentage (%) of referred peer clients initiating PrEP in the intervention group.

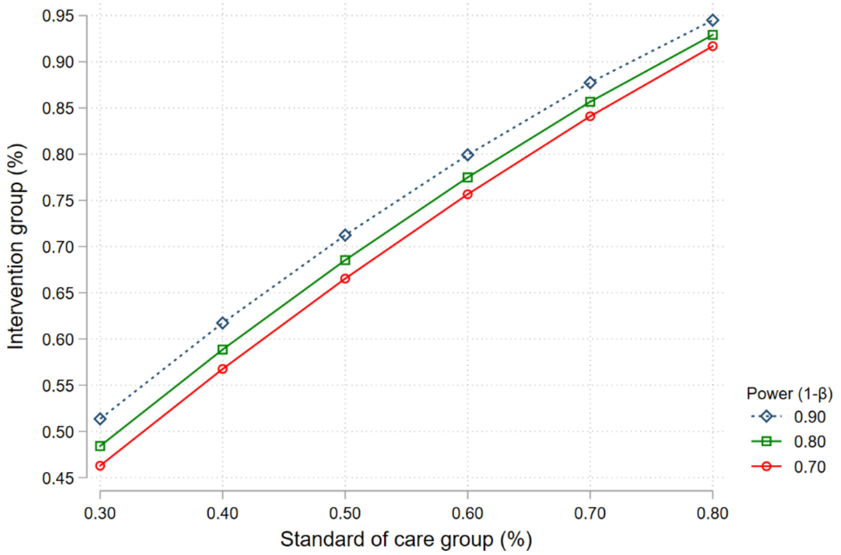

Table 10. Increase in the % of referred peer clients initiating PrEP in the intervention group. ICC: 0.05; clusters (providers) per arm: 40; units (clients) per cluster: 3 (75% of suggested clients referred)

Power (%)	Standard of care (%, p1)	Intervention (%, p2)	Increase (%, p2-p1)
70	30	46.3	16.3
	40	56.8	16.8
	50	66.5	16.5
	60	75.7	15.7
	70	84.1	14.1
	80	91.7	11.7
80	30	48.4	18.4
	40	58.9	18.9
	50	68.5	18.5
	60	77.5	17.5
	70	85.7	15.7
	80	92.9	12.9
90	30	51.4	21.4
	40	61.7	21.7
	50	71.2	21.2
	60	79.9	19.9
	70	87.8	17.8
	80	94.5	14.5

Participant retention & withdrawal

If we do not achieve our aim 1 pilot metrics of success (including our ability to follow-up with peers), we will revise the peer PrEP referral + HIVST delivery model with input from stakeholders before conducting the aim 2 trial. If necessary, we will try one of the alternative peer follow-up strategies outlined in aim 1, and in the worst-case scenario, we will have peer providers report peer client outcomes (an approach that has been used in other high-impact studies using peer-based health interventions[78]). In the aim 2 trial, we will confirm self-reported PrEP initiation and adherence among a subset of peer clients with drug levels in DBS.

Participants may voluntarily withdraw from the study for any reason at any time. The site investigators may also withdraw participants from the study in order to protect their safety and/or if they are unwilling or unable to comply with required study procedures. Reasons for withdrawal will be recorded.

PrEP discontinuation

PrEP initiation and continuation will be in accordance with Kenyan PrEP guidelines. Use of PrEP may be interrupted by the site investigators or Kenyan clinicians due to safety concerns for the participant or use of concomitant medications that could interfere with PrEP or present a safety concern. All treatment interruptions will be documented.

Referral to continued PrEP care

For participants that wish to continue PrEP care following the randomization trial, participants will remain enrolled in PrEP at the healthcare facility at which they had enrolled in PrEP. PrEP will then be prescribed and administered via this physician who will provide a 3-month supply of PrEP.

PrEP drugs

Tenofovir disoproxil fumarate (or TDF, 9-[(R)-2-[[bis [[(isopropoxycarbonyl) oxy] methoxy] phosphinyl] methoxy] propyl] adenine fumarate), emtricitabine (or FTC, 5-fluoro-1-(2R,5S)-[2-(hydroxymethyl)-1,3-oxatholan-5-yl]cytosine), and lamivudine (or 3TC, 2',3'-dideoxy-3'-thiacytidine 4-Amino- 1-[(2R,5S)- 2-(hydroxymethyl)- 1,3-oxathiolan-5-yl]- 1,2-dihydropyrimidin- 2-one) are reverse transcriptase inhibitors that have been approved for the treatment of HIV infection in humans in Kenya and the United States. A fixed-dose, oral co-formulation of FTC/TDF has also been approved for HIV prevention in Kenya and the United States. The WHO recommends TDF-containing medications as PrEP, which includes TDF combined with FTC as well as potentially TDF alone and TDF combined with lamivudine (or 3TC, a medication closely related to FTC). Any TDF-containing medications that align with WHO and Kenya national guidelines for PrEP will be used in this study. PrEP will be prescribed for once-daily oral use. Study medication will be provided by the Kenyan MoH and stored in accordance with the drug manufacturer's recommendations.

HIVST kits

ChemBio manufactures the SURE CHECK HIV 1/2 Assay, a blood-based HIVST that has 99.7% sensitivity, 99.9% specificity, presents results in 15 minutes, and is approved by the US FDA [37]. Results are easy to interpret (1 line to indicate HIV-negative, 2 lines to indicate HIV-positive, and no lines to indicate an invalid result). HIVST kits serve as a preliminary test result and must be followed-up with confirmatory facility-based HIV testing to indicate a diagnosis. Additionally, we will use other WHO-approved blood-based HIVST kits to mitigate supply chain or any other challenges that may arise.

Limitations

This randomization trial has some potential limitations that are important to note. Foremost, the trial will encourage participants to recruit 4 peer clients to the study. The limitation of 4 peer clients may inherently exclude some AGYW who have a high risk of HIV infection and would be interested in PrEP initiation through a peer PrEP referral+ HIVST model. Further, peer clients recruited to the study and randomized to the "formal peer PrEP referral + HIVST" arm, will be provided 2 HIVST kits to test themselves (independently or with assistance from the participant) or to test a primary sexual partner. Some AGYW may benefit from testing multiple sex partners (e.g., in sex worker relationships). Although a focus on partner testing is outside the scope of this study, future research should be considered in this area.

Additionally, the randomization of participants to either the "formal peer PrEP referral+ HIVST" arm or the "informal peer PrEP referral" arm may lead to feelings of frustration among participants who had hoped to be randomized to the formal intervention delivery arm which may lead to resentment towards the research team. Feelings of frustration are attempted to be mitigated by ongoing peer support (word-of-mouth) to enroll in PrEP in the informal delivery arm.

Finally, in the randomized trial, peers recruited to the study will be supplied HIVST kits free of cost. In settings outside of a research or intervention setting, AGYW will have to purchase HIVST kits at cost which may influence the feasibility of this model. The randomization trial aims to address this challenge by conducting a cost analysis will which be used to inform the design and development of a future peer PrEP referral + HIVST community randomization trial.

SAFETY

PrEP

Multinational studies demonstrated that PrEP (including FTC/TDF) was safe for use in women from Kenya and Uganda. [20] There were no statistically significant differences in the frequency of deaths, serious adverse events, adverse events overall, or key laboratory adverse events (specifically, creatinine elevation and phosphorus decrease) for those receiving PrEP compared to those receiving placebo in the Partners PrEP Study.
For the purposes of this study, only serious adverse events (SAEs) will be documented. SAEs felt to be related to PrEP will result in temporary hold of PrEP. In the case of temporary holds, the hold will continue until the event is stabilized or resolved. If the event resolves, PrEP may be reinitiated at the discretion of the Data Monitoring Committee (see "Study oversight" below), resuming safety monitoring. The severity of clinical symptoms will be scored using the Division of AIDS- NIH (DAIDS) Table (July 2017 Version) for Grading the Severity of Adult and Pediatric Adverse Events (AEs). Reporting on adverse events to relevant IRBs will be according to relevant regulations.

Pregnancy

Animal and human data, including from the Partners PrEP Study and Partners Demonstration Project, suggest safety of FTC/TDF when used by HIV infected women during pregnancy and breastfeeding. Other studies are exploring detailed safety of PrEP use in pregnancy. For this study, pregnant women interested in PrEP will be referred to antenatal care clinics for PrEP initiation and refills, as caring for a woman who is pregnant and on PrEP is outside of the scope of this study.

Social harm considerations

We have extensively considered the risk of social harms related to both PrEP use and peer referral to PrEP as well as peer HIVST delivery including risks of depression/anxiety and disclosure and stigma. Our extensive experience with longitudinal follow-up of heterosexual HIV sero-discordant couples and women at risk mitigates some of this risk, and we found very little risk of social harms or anxiety related to HIV self-testing from our ongoing Pharmacy PrEP study. Analyses of social harm related to peer PrEP referral + HIVST will be by relationship status and employment status/type, given the potential for differential employment and relationship risks. In the event of a clinical need (e.g., side effects, symptoms of a sexually transmitted infection), participants will be referred to nearby HIV clinics for care.

HUMAN SUBJECTS CONSIDERATIONS

The protocol, informed consent forms (for pilot participation, for focus group discussions, and for the randomization trial), and patient education and recruitment materials will be reviewed and approved by the institutional review boards at the Fred Hutchinson Cancer Center and the Scientific Ethics Review Unit (SERU) at the Kenya Medical Research Institute (KEMRI). All participants will provide informed consent before participation in the pilot study and randomization trial. Participants will be informed of the purpose of the study, the procedures to be followed and the risks and benefits of their participation. The consents forms will be translated into Kiswahili. Specifically, the participants will be informed that this novel study will answer critical questions on acceptability, feasibility, and facilitators/barriers to a model of peer PrEP referral + HIVST among AGYW in Kenya.

Study oversight

This study will be subject to oversight by an independent Data Safety Monitoring Committee that will periodically review data from the study, including study execution, adherence, HIV incidence, PrEP side effects, SAEs, and social harms by study arm. Adverse events (AEs) include any forms of verbal (e.g., yelling, fighting), physical (e.g., hitting), emotional (e.g., depression) or economic (e.g., job loss) abuse. While serious adverse events (SAEs) include hospitalization (for any condition, including those outside the study) or death. During data collection and study follow-up, participants will be asked to report if they’ve had any of these events and whether they are or are not study related. We have had a Data Monitoring Committee for many of our other ongoing studies. The independent data monitoring committee will provide recommendations to the study team as part of periodic reviews. Reports from all reviews will be provided for submission to overseeing IRBs and Ethics Committees (ECs).

Risks

Participants may become embarrassed, worried, or anxious when completing their HIV risk assessment and/or receiving HIV counseling at healthcare facilities for PrEP enrollment. They also may become worried or anxious while waiting for their HIV test results following HIV self-testing. Participants who learn that they have HIV may experience anxiety or depression related to their test results. Further, participants may become embarrassed or worried when they approach peers about PrEP, encourage peers to HIVST/assist peers to HIVST, or when they disclose their HIV status and/or PrEP use to peers (should they choose to do so). At all study sites, individual counseling will be provided by researchers who have been trained in specific issues related to HIV risk, HIV prevention, HIV acquisition, and HIV treatment.

Although study sites will make every effort to protect participant privacy and confidentiality, it is possible that participants' involvement in the study could become known to others, particularly as this project will utilize a peer-delivered model. There is a possibility that social harms may result (i.e., because participants could become known as participating in studies involving HIV prevention). For example, participants could be treated unfairly, stigmatized, or discriminated against, or could have problems being accepted by their families and/or communities. Talking about HIV or PrEP and finding out HIV test results could cause problems between a participant and their sexual partner(s), including the potential for intimate partner violence (e.g., any physical, verbal, emotional, or economic harm). Understanding the risk/benefit balance for confidential delivery of PrEP services in community settings is an explicit goal of this project. Moreover, we have extensive experience with the strategies to minimize the potential for social harms in populations participating in HIV prevention studies.

Risks and side effects related to PrEP include gastrointestinal intolerance and rarely more serious side effects; these are detailed on the package insert and this project is not testing PrEP itself but its delivery. The medical risks of HIV testing using blood collection are small.

Protection against risk

The study team has extensive experience with counseling about HIV risk, PrEP, HIVST, and strategies for HIV prevention in general. Study procedures will include quantitative surveys (e.g., PrEP initiation, peer PrEP referral) as well as qualitative interviews to understand acceptability of the model. PrEP will be provided at specified healthcare facilities and will follow Kenyan clinical guidelines. Counseling about antiretroviral-based HIV prevention will include messaging describing the benefits of all strategies, based on evolving available data and national policies / national roll-out of antiretrovirals (including earlier treatment and PrEP) for HIV prevention.

For data collection, standardized questionnaires will be used that will include questions on sensitive topics, including sexual behavior, depression, alcohol use, and stigma. We have extensive experience with these questionnaires from our prior studies and the expertise and counseling resources required to attend to study participants (e.g., those with depression). We have published on very low rates of social harm and intimate partner violence in our prospective studies of HIV sero-discordant couples, which likely reflects the counseling available to couples; for women at risk, we have extensive experience with management of potential social harms, through our prevention studies.

To minimize risks to minors, we will only enroll young women ≥16 to 17 years into the study if they are emancipated minors and thus able to legally provide consent for participation in research. Kenyan law acknowledges women ages 14 to 17 who have become pregnant as emancipated minors. We have successfully enrolled emancipated minors into a number of prior studies, including PrEP clinical trials. In addition, Kenya national policy guidelines explicitly permit PrEP use in this age group.

The risks from the anticipated activities will be no greater than in our previous studies; in fact, given the proven prevention benefits of PrEP and its national roll-out in Kenya, risks are anticipated to be less than in some of our prior studies. We feel the risks associated with the study are small. The benefits are consistent with clinical care benefits and cultural expectations, and they follow the established standard with IRB approval in our other studies. We therefore believe the balance of benefit and risk is appropriate.

Benefits

All participants will have the benefit of using novel HIVST kits, which are being scaled-up by the Kenyan Ministry of Health. Further, participants will have the added benefit of pioneering a combined peer PrEP referral and HIVST model. HIV prevention practices, according to national guidelines, will be provided to all participants enrolled in this study. This will include risk reduction counseling, addressing sexually transmitted infections, and access to condoms. In addition, participants and others may also benefit in the future from information learned from this study. There may be no other direct benefits to participants in this study.

Care for persons identified as HIV infected

This study may identify persons who are infected with HIV, either as part of the study screening process (e.g., when peers utilize HIVST) or during follow-up of enrolled participants. Study staff will provide participants with their HIV test results in the context of post-test counseling. Persons identified as HIV infected during the study screening process, and who do not meet eligibility criteria will be referred to nearby clinics where they can receive free HIV care and treatment services. For participants who are HIV infected and who also become pregnant during follow-up, every effort will be made to facilitate access to programs for preventing mother-to-child HIV transmission for appropriate antiretroviral treatment to reduce the probability of HIV transmission from mother to child.

Benefits to the community

An important goal of this study is to achieve the study objectives in a way that provides benefits to the

community that endure beyond the proposed study lifetime regardless of the specific outcome of the study. Some of these community benefits include development of optimized approaches to HIV prevention care and community awareness of comprehensive HIV prevention.

Importance of the knowledge to be gained

Knowledge gained from the studies proposed in this application will include information about optimal delivery of PrEP for HIV prevention among a vulnerable group (e.g., AGYW), which may have substantial impact on the global burden of HIV.

Treatment for injury

Participants will be asked to inform the clinic staff if they feel they have been injured because of taking part in the study. Injuries may also be identified during laboratory testing, medical histories, and physical examinations at the specified healthcare facilities where participants will have access to PrEP. Treatment for adverse events related to study participation will be provided by the treatment clinic. If treatment is required that is beyond the capacity of the clinic, the clinic staff will refer the participant to appropriate services or organizations that can provide care for the injury. Participants will be financially responsible for treatment for injuries.

Study records

Implementation investigators will maintain, and store in a secure manner, complete, accurate, and current study records throughout the study. Study records include administrative documentation and regulatory documentation as well as documentation related to each participant enrolled in the study, including informed consent forms, data forms, notations of all contacts with the participant, and all other source documents.

Confidentiality

Every effort will be made to protect participant privacy and confidentiality to the extent possible. Personal identifying information will be retained at the local study sites and not forwarded to Fred Hutch. The sites will use their standard operating procedures for confidentiality protection that reflects the input of study staff and community representatives to identify potential confidentiality issues and strategies to address them.

All study-related information will be stored securely at the study sites. All participant information will be stored in areas with limited access. Data collection, administrative forms, and other reports will be identified only by a coded number to maintain participant confidentiality. All records that contain names or other personal identifiers, such as locator forms and informed consent forms, will be stored separately from study records identified by code number. All local databases will be secured with password-protected access systems. Forms, lists, logbooks, appointment books, and any other listings that link participant ID numbers to other identifying information will be stored in a separate, locked file in an area with limited access. During FGDs, we will ask participants not to refer to each other by name. During the peer providers trainings, we will instruct peer providers to not enter any identifying information about peer clients in the WhatsApp group messages. Any personal identifiers will he removed from the qualitative data transcripts and immediately deleted from WhatsApp messages by the study team. Recordings will be destroyed at the end of the study. WhatsApp group messages will not be stored long term (they will be periodically deleted), and the WhatsApp groups will be shut down at the end of the intervention period for each group of peer providers in the intervention arm.

Dissemination Plan

The study team is committed to the public dissemination of results from the pilot study and the

randomization trial to participants, local stakeholders in Kenya, the global scientific community, and U.S., Kenyan, and global policymakers. Dissemination of study results will follow principles of good participatory practice. Results will be published in conference abstracts and peer-reviewed journals. Study results will be disseminated through presentations to local stakeholders and policymakers in Kenya, including the Ministry of Health.

TIMELINE

The findings from the pilot study and randomization trial will provide important information on how to increase PrEP initiation and continuation among young women at HIV risk in Kenya, which can inform Kenya MOH guidelines around PrEP delivery. Additionally, these findings will inform the development of an R01 proposal for a community-randomized trial and budget impact analysis of peer PrEP referral + HIVST delivery in Kenya, should aims 1 & 2 find the delivery model promising among young women **(Table 11).**

**Table 11. Timeline and outcomes**		**R00 phase:**		
		**Y1**	**Y2**	**Y3**
**Aim 1: Pilot**	Protocol prep. & IRB submission	**X**		
	Pilot & refine Aim 1 delivery model	**X**		
	Conduct & analyze FGDs	**X**		
	Manuscript(s) preparation	**X**		
**Aim 2: Randomization Trial**	Protocol prep. & IRB submission	**X**		
	Enrollment, data collect, analysis	**X**	**X**	**X**
	Collect & analyze costing data		**X**	**X**
	Manuscript(s) preparation			**X**
**Planned R01 submission (Community Randomization Trial)**				**X**

REFERENCES

1. *Miles to Go: Closing Gaps, Breaking Barriers, Righting Injustices*. http://www.unaids.org/sites/default/files/media_asset/miles-to-go_en.pdf (2018).
2. Cherutich, P. *et al.* Lack of knowledge of HIV status a major barrier to HIV prevention, care and treatment efforts in Kenya: results from a nationally representative study. *PloS One* **7**, e36797 (2012).
3. *Kenya AIDS Response Progress Report 2016*. https://nacc.or.ke/wp-content/uploads/2016/11/Kenya-AIDS-Progress-Report_web.pdf (2016).
4. Harper, Gary W., Riplinger, Andrew J., Neubauer, Leah C., Murphy, Alexandra G., Velcoff, Jessica, Bangi, Audrey K. Ecological factors influencing HIV sexual risk and resilience among young people in rural Kenya: implications for prevention. *Health Educ. Res.* **29**, 131–146 (2014).
5. Ziraba A, Orindi B, Muuo S, Floyd S, Birdthistle IJ, Mumah J, Osindo J, Njoroge P, Kabiru CW. Understanding HIV risks among adolescent girls and young women in informal settlements of Nairobi, Kenya: Lessons for DREAMS. *PLoS ONE* **13**, (2018).
6. *Kenya AIDS Strategic Framework*. http://www.undp.org/content/dam/kenya/docs/Democratic%20Governance/KENYA%20AIDS%20STRATEGIC%20FRAMEWORK.pdf (2018).
7. Grant, R. M. *et al.* Preexposure chemoprophylaxis for HIV prevention in men who have sex with men. *N. Engl. J. Med.* **363**, 2587–2599 (2010).
8. Baeten, J. M. *et al.* Antiretroviral Prophylaxis for HIV Prevention in Heterosexual Men and Women. *N. Engl. J. Med.* **367**, 399–410 (2012).
9. Thigpen, M. C. *et al.* Antiretroviral preexposure prophylaxis for heterosexual HIV transmission in Botswana. *N. Engl. J. Med.* **367**, 423–434 (2012).
10. Choopanya, K. *et al.* Antiretroviral prophylaxis for HIV infection in injecting drug users in Bangkok, Thailand (the Bangkok Tenofovir Study): a randomised, double-blind, placebo-controlled phase 3 trial. *Lancet Lond. Engl.* **381**, 2083–2090 (2013).
11. FDA approves first drug for reducing the risk of sexually acquired HIV infection News. *U.S. Food and Drug Administration* https://aidsinfo.nih.gov/news/1254/fda-approves-first-drug-for-reducing-the-risk-of-sexually-acquired-hiv-infection (2012).
12. *Guideline on when to start antiretroviral therapy and on pre-exposure prophylaxis for HIV*. 78 http://apps.who.int/iris/bitstream/handle/10665/186275/9789241509565_eng.pdf;jsessionid=E57E17E2CF940BC43AA925A899C860FF?sequence=1 (2015).
13. Thuo N, Ngure K, Ogello V, Kamolloh K. “So That I Don’t Get Infected Even If I Have Sex With Someone Who Is Positive:” Factors Influencing PrEP Uptake Among Young Women in Kenya. in (2018).
14. Celum C, Delany-Moretlwe S, Hosek S, Dye B, Bekker L, Mgodi N, Mabuza W, Mvuyane G, Mukaka S, Donnell D, Pathak S, Noble H, Lennon D, Fogel J, Anderson P. Risk behavior, perception, and reasons for PrEP among young African women in HPTN 082. in (2018).
15. Baeten, J. M., Haberer, J. E., Liu, A. Y. & Sista, N. Preexposure prophylaxis for HIV prevention: where have we been and where are we going? *J. Acquir. Immune Defic. Syndr. 1999* **63 Suppl 2**, S122-129 (2013).
16. Grant, R. M. *et al.* Uptake of pre-exposure prophylaxis, sexual practices, and HIV incidence in men and transgender women who have sex with men: a cohort study. *Lancet Infect. Dis.* **14**, 820–829 (2014).
17. Anderson, P. L. *et al.* Emtricitabine-tenofovir concentrations and pre-exposure prophylaxis efficacy in men who have sex with men. *Sci. Transl. Med.* **4**, 151ra125 (2012).

18. McCormack, S. *et al.* Pre-exposure prophylaxis to prevent the acquisition of HIV-1 infection (PROUD): effectiveness results from the pilot phase of a pragmatic open-label randomised trial. *Lancet Lond. Engl.* **387**, 53–60 (2016).

19. Liu, A. Y. *et al.* Preexposure Prophylaxis for HIV Infection Integrated With Municipal- and Community-Based Sexual Health Services. *JAMA Intern. Med.* **176**, 75–84 (2016).

20. Baeten, J. M. *et al.* Integrated Delivery of Antiretroviral Treatment and Pre-exposure Prophylaxis to HIV-1-Serodiscordant Couples: A Prospective Implementation Study in Kenya and Uganda. *PLoS Med.* **13**, e1002099 (2016).

21. Sullivan, P. The impact of pre-exposure prophylaxis with TDF/FTC on HIV diagnoses, 2012-2016, United States. (2018).

22. Buchbinder, S. *et al.* Getting to zero new HIV diagnoses in San Francisco: What will it take? (2018).

23. Grulich, A. *et al.* Rapid reduction in HIV diagnosis after targeted PrEP implementation in NSW, Australia. (2018).

24. WHO. Guidelines on HIV Self-Testing and Partner Notification. (2016).

25. Oluoch L, Roxby A, Wald A, Selke S, Margaret A, Mugo N, Cohan B, Ngure K, Gakuo S, Kiptinness C. Low uptake of pre-exposure prophylaxis among Kenyan adolesent girls at risk of HIV. in (2019).

26. Mugo, Nelly R., Ngure, Kenneth., Kiragu,Michael., Irungu, Elizabeth., Kilonzo, Nduku. PrEP for Africa: What we have learnt and what is needed to move to program implementation. *Curr. Opin. HIV AIDS* **11**, 80–86 (2016).

27. Shangani S, Escudero D, Kirwa K, Harrison A, Marshall B, Operario D. Effectiveness of peer-led interventions to increase HIV testing among men who have sex with men: a systematic review and meta-analysis. *AIDS Care* 1003–1013 (2017).

28. Safren SA, O'Cleirigh C, Skeer MR, Driskell J, Goshe BM, Covahey C, Mayer KH. Demonstration and evaluation of a peer-delivered, individually-tailored, HIV prevention intervention for HIV-infected MSM in their primary care setting. *AIDS Behav.* **15**, 949–958 (2011).

29. Ortblad K, Kibuuka Musoke D, Ngabirano T, Nakitende A, Magoola J, Kayiira P, Taasi G, Barresi LG, Haberer JE, McConnell MA, Oldenburg CE, Bärnighausen T. Direct provision versus facility collection of HIV self-tests among female sex workers in Uganda: A cluster-randomized controlled health systems trial. *PLoS Med* **14**, (2017).

30. Chanda MM, Ortblad KF, Mwale M, Chongo S, Kanchele C, Kamungoma N, Fullem A, Dunn C, Barresi LG, Harling G, Bärnighausen T, Oldenburg CE. HIV self-testing among female sex workers in Zambia: A cluster randomized controlled trial. *PLoS Med* **14**,.

31. George A, Blankenship KM. Peer Outreach Work as Economic Activity: Implications for HIV Prevention Interventions among Female Sex Workers. *PLoS ONE* **10**, (2015).

32. Shahmanesh M, Patel V, Mabey D, Cowan F. Effectiveness of interventions for the prevention of HIV and other sexually transmitted infections in female sex workers in resource poor setting: a systematic review. *Trop. Med. Int. Health TM IH* **13**, 659–679 (2008).

33. Bhattacharjee P, Prakash R, Pillai P, Isac S, Haranahalli M, Blanchard A, Shahmanesh M, Blanchard J, Moses S. Understanding the role of peer group membership in reducing HIV-related risk and vulnerability among female sex workers in Karnataka, India. *AIDS Care* 25 Suppl 1:S46-54. (2013).

34. Heffron, R. *et al.* Pre-exposure prophylaxis for HIV-negative persons with partners living with HIV: uptake, use, and effectiveness in an open-label demonstration project in East Africa. *Gates Open Res.* **1**, 3 (2017).

35. *Guidelines on Use of Antiretroviral Drugs for Treating and Preventing HIV Infections in Kenya - 2016 Edition*. https://static1.squarespace.com/static/57111dada3360ca8fd78159e/t/57a39ef06a4963d0020fcc63/1470

340850147/PrEP+Pages+Guidelines-on-Use-of-Antiretroviral-Drugs-for-Treating-and-Preventing-HIV-Infections-in-Kenya.pdf (2016).

36. Government of Kenya- Ministry of Health. *Guidelines for Conducting Adolescent HIV Sexual and Reproductive Health Research in Kenya*. https://icop.or.ke/wp-content/uploads/2016/10/Adolescents-Guidance-on-HIV-SRH-Research.pdf (2015).
37. U.S. Food and Drug Administration. First rapid home-use HIV kit approved. in (2012).
38. Kroenke, K. & Spitzer, R. L. The PHQ-9: A New Depression Diagnostic and Severity Measure. *Psychiatr. Ann.* **32**, 509–515 (2002).
39. Manea, L., Gilbody, S. & McMillan, D. Optimal cut-off score for diagnosing depression with the Patient Health Questionnaire (PHQ-9): a meta-analysis. *CMAJ Can. Med. Assoc. J.* **184**, E191–E196 (2012).
40. Schwarzer, R. & Jerusalem, M. *Measures in health psychology: A user's porfolio.* (NFER-NELSON, 1995).
41. Damschroder, L. J. *et al.* Fostering implementation of health services research findings into practice: a consolidated framework for advancing implementation science. *Implement. Sci.* **4**, 50 (2009).
42. Simoni, J. M. *et al.* Debrief Reports to Expedite the Impact of Qualitative Research: Do They Accurately Capture Data from In-depth Interviews? *AIDS Behav.* (2019) doi:10.1007/s10461-018-02387-3.
43. Thomas, D. A General Inductive Approach for Analyzing Qualitative Evaluation Data. *Am. J. Eval.* **27**, 237–46 (2006).
44. Bernard, H., Wutich, A. & Ryan, G. *Analyzing qualitative data: Systematic approaches*. (SAGE publications, 2016).
45. Hsieh, H.-F. & Shannon, S. E. Three approaches to qualitative content analysis. *Qual. Health Res.* **15**, 1277–1288 (2005).
46. Stirling, J. Thematic networks: An analytic tool for qualitative research. *Qual. Res.* **1**, 385–405 (2001).
47. Gale, N. K., Heath, G., Cameron, E., Rashid, S. & Redwood, S. Using the framework method for the analysis of qualitative data in multi-disciplinary health research. *BMC Med. Res. Methodol.* **13**, 117 (2013).
48. Barbour, R. S. Checklists for improving rigour in qualitative research: a case of the tail wagging the dog? *BMJ* **322**, 1115–1117 (2001).
49. Mays, N. & Pope, C. Rigour and qualitative research. *BMJ* **311**, 109–112 (1995).
50. Patel, R. C. *et al.* 'Since both of us are using antiretrovirals, we have been supportive to each other': facilitators and barriers of pre-exposure prophylaxis use in heterosexual HIV serodiscordant couples in Kisumu, Kenya. *J. Int. AIDS Soc.* **19**, 21134 (2016).
51. Ware, N. C. *et al.* What's love got to do with it? Explaining adherence to oral antiretroviral pre-exposure prophylaxis for HIV-serodiscordant couples. *J. Acquir. Immune Defic. Syndr. 1999* **59**, 463–468 (2012).
52. Ngure, K. *et al.* The role of male partners in women's participation in research during pregnancy: a case study from the partners demonstration project. *Reprod. Health* **14**, 160 (2017).
53. Pintye, J. *et al.* 'I Did Not Want to Give Birth to a Child Who has HIV': Experiences Using PrEP During Pregnancy Among HIV-Uninfected Kenyan Women in HIV-Serodiscordant Couples. *J. Acquir. Immune Defic. Syndr. 1999* **76**, 259–265 (2017).
54. Ngure, K. *et al.* Feasibility and acceptability of HIV self-testing among pre-exposure prophylaxis users in Kenya. *J. Int. AIDS Soc.* **20**, 21234 (2017).
55. Ngure, K. *et al.* Delivering safer conception services to HIV serodiscordant couples in Kenya: perspectives from healthcare providers and HIV serodiscordant couples. *J. Int. AIDS Soc.* **20**, 21309 (2017).
56. Ngure, K. *et al.* 'I never thought that it would happen … ' Experiences of HIV seroconverters among HIV-

discordant partnerships in a prospective HIV prevention study in Kenya. *AIDS Care* **28**, 1586–1589 (2016).

57. Ngure, K. *et al.* My intention was a child but I was very afraid: fertility intentions and HIV risk perceptions among HIV-serodiscordant couples experiencing pregnancy in Kenya. *AIDS Care* **26**, 1283–1287 (2014).
58. Ngure, K. *et al.* A qualitative study of barriers to consistent condom use among HIV-1 serodiscordant couples in Kenya. *AIDS Care* **24**, 509–516 (2012).
59. Ngure, K. *et al.* I Knew I Would Be Safer. Experiences of Kenyan HIV Serodiscordant Couples Soon After Pre-Exposure Prophylaxis (PrEP) Initiation. *AIDS Patient Care STDs* **30**, 78–83 (2016).
60. Pintye, J. *et al.* Fertility Decision-Making Among Kenyan HIV-Serodiscordant Couples Who Recently Conceived: Implications for Safer Conception Planning. *AIDS Patient Care STDs* **29**, 510–516 (2015).
61. Patel, R. C. *et al.* What motivates serodiscordant couples to prevent HIV transmission within their relationships: findings from a PrEP implementation study in Kenya. *Cult. Health Sex.* **20**, 625–639 (2018).
62. Patel, R. C. *et al.* Facilitators and Barriers of Antiretroviral Therapy Initiation among HIV Discordant Couples in Kenya: Qualitative Insights from a Pre-Exposure Prophylaxis Implementation Study. *PloS One* **11**, e0168057 (2016).
63. Patel, R. *et al.* HIV-positive men's experiences with integrated family planning and HIV services in western Kenya: integration fosters male involvement. *AIDS Patient Care STDs* **28**, 418–424 (2014).
64. Izugbara, C. O. *et al.* 'It takes more than a fellowship program': reflections on capacity strengthening for health systems research in sub-Saharan Africa. *BMC Health Serv. Res.* **17**, 696 (2017).
65. Curran, K. *et al.* 'If I am given antiretrovirals I will think I am nearing the grave': Kenyan HIV serodiscordant couples' attitudes regarding early initiation of antiretroviral therapy. *AIDS Lond. Engl.* **28**, 227–233 (2014).
66. Musoke, P. *et al.* Men's hopes, fears and challenges in engagement in perinatal health and the prevention of mother-to-child transmission of HIV in rural Kenya. *Cult. Health Sex.* 1–14 (2018) doi:10.1080/13691058.2018.1426785.
67. Kwena, Z. A. *et al.* Jaboya ('Sex for Fish'): A Qualitative Analysis of Contextual Risk Factors for Extramarital Partnerships in the Fishing Communities in Western Kenya. *Arch. Sex. Behav.* **46**, 1877–1890 (2017).
68. Rogers, A. J. *et al.* Implementation of repeat HIV testing during pregnancy in Kenya: a qualitative study. *BMC Pregnancy Childbirth* **16**, 151 (2016).
69. Hilliard, S. *et al.* Perceived Impact of a Land and Property Rights Program on Violence Against Women in Rural Kenya: A Qualitative Investigation. *Violence Women* (2016) doi:10.1177/1077801216632613.
70. Onono, M. *et al.* 'You Know You Are Sick, Why Do You Carry A Pregnancy Again?' Applying the Socio-Ecological Model to Understand Barriers to PMTCT Service Utilization in Western Kenya. *J. AIDS Clin. Res.* **6**, (2015).
71. Camlin, C. S., Kwena, Z. A., Dworkin, S. L., Cohen, C. R. & Bukusi, E. A. 'She mixes her business': HIV transmission and acquisition risks among female migrants in western Kenya. *Soc. Sci. Med. 1982* **102**, 146–156 (2014).
72. Walcott, M. M., Hatcher, A. M., Kwena, Z. & Turan, J. M. Facilitating HIV status disclosure for pregnant women and partners in rural Kenya: a qualitative study. *BMC Public Health* **13**, 1115 (2013).
73. Grant RM, Anderson PL, McMahan V, Liu A, Amico KR, Mehrotra M, Hosek S, Mosquera C, Casapia M, Montoya O, Buchbinder S, Veloso VG, Mayer K, Chariyalertsak S, Bekker L-G, Kallas EG, Schechter M, Guanira J, Bushman L, Burns DN, Rooney JF, Glidden DV, iPrEx study team. Uptake of pre-exposure prophylaxis, sexual practices, and HIV incidence in men and transgender women who have sex with men: a cohort study. *Lancet Infect. Dis.* **14**, 820–829 (2014).
74. Sharma M, Smith JA, Farquhar C, Ying R, Cherutich P, Golden M, Wamuti B, Bukusi D, Spiegel H,

Barnabas RV. Assisted partner notification services are cost-effective for decreasing HIV burden in western Kenya. *AIDS Lond. Engl.* **32**, 233–241 (2018).

75. Sharma M, Farquhar C, Ying R, Krakowiak D, Kinuthia J, Osoti A, Asila V, Gone M, Mark J, Barnabas RV. Modeling the Cost-Effectiveness of Home-Based HIV Testing and Education (HOPE) for Pregnant Women and Their Male Partners in Nyanza Province, Kenya. *J. Acquir. Immune Defic. Syndr. 1999* S174-180 (2016).
76. Ying R, Sharma M, Heffron R, Celum CL, Baeten JM, Katabira E, Bulya N, Barnabas RV. Cost-effectiveness of pre-exposure prophylaxis targeted to high-risk serodiscordant couples as a bridge to sustained ART use in Kampala, Uganda. *J. Int. AIDS Soc.* **18**, (2015).
77. Smith JA, Sharma M, Levin C, Baeten JM, van Rooyen H, Celum C, Hallett TB, Barnabas RV. Cost-effectiveness of community-based strategies to strengthen the continuum of HIV care in rural South Africa: a health economic modelling analysis. *Lancet* **2**, e159-168 (2015).
78. Curran GM, Bauer M, Mittman B, Pyne JM, Stetler C. Effectiveness-implementation hybrid designs: combining elements of clinical effectiveness and implementation research to enhance public health impact. *Med. Care* **50**, 217–226 (2012).
