# Supplementary material for: Effect of enhanced peer PrEP referral with HIV self-testing delivery among young Kenyan women: A randomized controlled trial of peer networks
Source: PLoS Med. 2026 Mar 30;23(3):e1005023. doi: 10.1371/journal.pmed.1005023 (PMC13046272; doi:10.1371/journal.pmed.1005023)
Supplement: S1 Statistical Analysis Plan — (PDF) [file pmed.1005023.s004.pdf]

Peer PrEP referral + HIV self-test delivery for PrEP initiation among young Kenyan women:

Pilot study & randomized-controlled implementation trials

STATISTICAL ANALYSIS PLAN

Confidential

Version 1.6, July 15, 2024.

Prepared by:

Katrina Ortblad[1], ScD, MPH, Assistant Professor,
Fei Gao[2], PhD, Assistant Professor
Carlos Culquichicon[3], MSPH, Research Assistant

[1]Public Health Sciences Division, Fred Hutchinson Cancer Center, Seattle, USA
[2]Vaccines and Infectious Diseases Division, Fred Hutchinson Cancer Center, Seattle, USA
[3]Department of Epidemiology, University of Washington, Seattle, USA

Table of contents

1. Introduction 3

2. Study rationale 3

3. Study overview and objectives 3
   3.1. Study overview 3
   3.2. Study design 5
   3.3. Eligibility criteria 6
   3.4. Study procedures 7

4. Study endpoints 7
   4.1. Primary & secondary PrEP outcomes 7
   4.2. Process outcomes 8

5. Sample size justification 9

5.1. Sample size calculation 9

6. Randomization and masking 10
   6.1. Randomization 11

7. Data collection 11
   7.1. Database 11
   7.2. Participant identifiers 11

8. Statistical considerations 11
   8.1. Analysis sets 11

9. Interim monitoring 12

10. Data analysis 12
   10.1. Baseline characteristics 12
   10.2. Effect size estimation 12
   10.3. Missing values and "don't know" responses 12
   10.4 Sensitivity analyses 13
   10.5 Adverse events 13

11. References 14

1. Introduction

This document (Statistical Analysis Plan, SAP) describes the planned analysis and reporting for the study titled, “Peer PrEP referral + HIV self-test delivery for PrEP initiation among young Kenyan women: randomized implementation trial,” also known as the “Peer PrEP cRCT”. This trial is a joint collaboration between the Fred Hutchinson Cancer Center (Fred Hutch) and the Jomo Kenyatta University of Agriculture and Technology (JKUAT; R00 MH121166, PI: Ortblad). It includes specifications for the statistical analyses and tables to be prepared for final reporting for the randomized trial.

The planned analyses described in this SAP will be included in future manuscripts. Note, however, that exploratory analyses not necessarily identified in this SAP may be performed to support the analysis. All post-hoc or unplanned analyses which have not been delineated in this SAP will be clearly documented as such in the final study reporting, manuscripts, or any other document or submission.

2. Study rationale

Few young women at risk of HIV infection are initiating daily, oral pre-exposure prophylaxis (PrEP) for HIV prevention in Kenya, thus we propose developing, refining, and testing a new model to increase PrEP initiation among young women at high HIV risk that has not yet been explored: peer PrEP referral + HIV self-testing (HIVST) delivery (1).

Through a pilot based on qualitative formative research and stakeholder engagement, we developed and refined an acceptable and feasible model where young (≥16 to 24 years) female PrEP users refer their peers to PrEP using materials we determine to be appropriate (e.g., pamphlets) and HIVST kits. In phase 2 of this study, we plan to conduct a 2-arm hybrid effectiveness-implementation randomized trial to test the effect of the refined peer PrEP referral + HIVST delivery model on PrEP initiation among young Kenyan women compared to informal peer PrEP referral, where young women are encouraged to refer four peers to PrEP services by word-of-mouth, as is ongoing in Kenya. We hypothesize that relative to informal, standard word-of-mouth peer PrEP referral (our control group), formalized/enhanced peer PrEP referral + HIVST delivery will increase PrEP adoption (i.e., initiation, retention, and adherence) among peers, be low cost, and have high fidelity among young Kenyan women.

3. Study overview and objectives

3.1. Study overview

Table 1. Overview of the Peer PrEP cRCT components

**Protocol title:**	Peer PrEP referral + HIV self-test delivery for PrEP initiation among young Kenyan women: randomized implementation trial
**Short title:**	Peer PrEP Study
**Design:**	This study is a hybrid effectiveness-implementation randomized trial
**Population:**	We will enroll two different population types in this study:<br>1. <u>Index peers</u>: (Also called *PrEP users* or *peer providers* in our formative research and pilot study). Adolescent girls and young women ≥16-24 years[1] old using PrEP for at least one month in the past 12 months.

	2. <u>Referred peers</u>: (Also called *peers* or *PrEP clients* in our formative research and pilot study). Female peers ≥16-24 years old of the index peer whom the index peer thinks has behaviors associated with HIV risk and may be interested in and benefit from daily oral PrEP.<br><br>[1]16- and 17-year-olds must be emancipated minors; all participants must be able and willing to provide informed consent.
**Randomization:**	1:1 enhanced group: standard group
**Study groups:**	<u>Intervention: Enhanced peer referral</u> *(previously formal peer referral)*<br><br>• **Index peers** randomized to the enhanced group will receive the package of implementation strategies below and be encouraged to refer four peers to PrEP:<br>o A one-day training on HIVST & PrEP and how to have conversations about and refer peers to PrEP service<br>o PrEP informational brochures (n=4, *1 per peer*)<br>o HIVST kits (n=8, *2 per peer*)<br>o PrEP referral cards (MOH-style), with information on nearby facilities delivering free PrEP services (n=4, *1 per peer)*<br><br>• **Referred peers** referred to PrEP services by index peers in this group should receive the described package of implementation strategies to support PrEP referral.<br><br><u>Control: Standard peer referral</u> *(previously informal peer referral):*<br><br>• **Index peers** randomized to the standard group will be encouraged to refer four peers to PrEP services using existing informal word-of-mouth referral, common among AGYW. They will receive:<br>o PrEP referral cards (MOH-style), with information on nearby facilities delivering free PrEP services (n=4, *1 per peer)*<br><br>• **Referred peers** referred to PrEP services by index peers in this group will be referred to PrEP services informally, as is ongoing in Kenya.
**Sample size:**	400 enrolled index and referred peers in total*<br>• 80 index peers<br>• Up to 320 referred peers<br><br>**The enrollment numbers for referred peers may be lower due to challenges in getting provide their contact information at the time of peer referral. Primary outcomes among referred peers will be reported by index peers in index peer follow-up surveys.*
**Follow-up:**	3 months from either the time of intervention delivery (index peers) or time when study staff received their contact information (referred peers)
**Study sites:**	Three counties in Central Kenya: Kiambu, Nairobi, and Murang'a (in the area surrounding the PHRD research clinic in Thika)
**Clinical outcomes:**	• <u>Primary</u>: PrEP initiation among referred peers, as reported by index peers at 3 months<br><br>• <u>Secondary</u>: recent HIV testing (since referral) and PrEP continuation (i.e., any refilling) among referred peers, as reported by index peers at 3 months. PrEP adherence (self-reported) among referred peers, as reported by referred peers at 3 months. PrEP continuation (since referral) among index peers, as reported by index peers at 3 months.
**Process outcomes**	• Number of peers referred to PrEP, reported by index peers at 3 months.

	• Linkage to care (i.e., visited a healthcare clinics), as reported by referred peers at 3 months<br>• HIVST use, reported by referred peers in the enhanced group at 3 months.
**Implementation outcomes:**	• Acceptability, feasibility, and appropriateness measured using validated scales (reported by index peers and referred peers at 3 months). Fidelity measured by the intervention materials delivered.

3.2. Study design

Two-arm cluster-randomized controlled trial (cRCT) to test effect of a model of peer PrEP referral enhanced with training and HIVST delivery compared to informal word-of-mouth peer PrEP referral (i.e., the existing standard) on PrEP initiation among Kenyan AGYW:

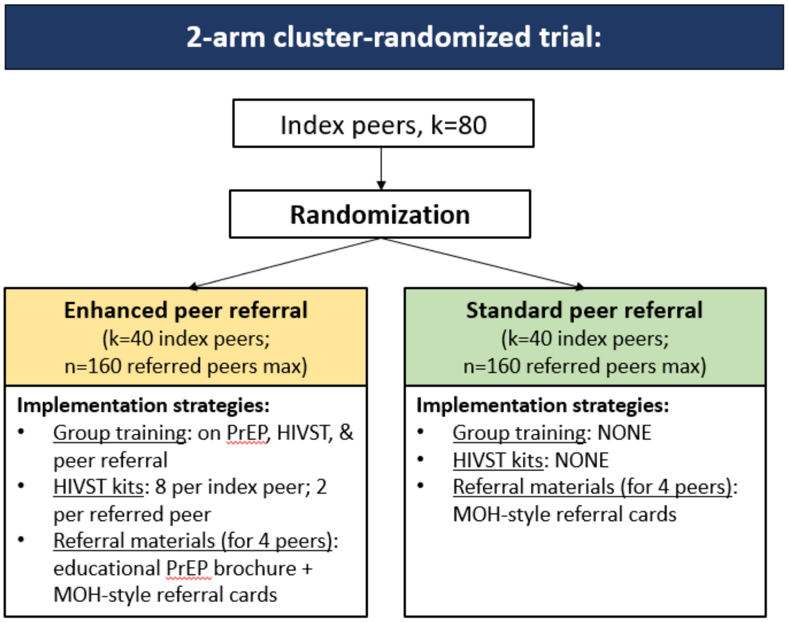

Figure 1. Study groups for the Peer PrEP cRCT.

3.3. Eligibility criteria

The eligibility criteria for trial participants and recruitment strategies are described below in **Table 2.**

Table 2. Eligibility criteria for index peers and referred peers in the Peer PrEP cRCT

	Eligibility	Ineligibility	Recruitment strategies
**Index peers**<br>(n=80)	• ≥16- 24 years old*<br>• Female<br>• Must have refilled or initiated PrEP (i.e., been dispensed PrEP).<br>• Not currently enrolled in a HIV study<br>• Can identify 4 peers at HIV risk who could benefit from PrEP.<br>• Able & willing to be randomized to the intervention, participate in research activities, and/or provide written informed consent	• Not between 16-24 years old<br>• Male<br>• Have not used PrEP.<br>• Cannot identify 4 peers who would benefit from PrEP.<br>• Is not able or willing to be randomized to the intervention, participate in research activities, and/or provide written informed consent.<br>• Currently enrolled in a HIV study.<br>• Illiterate	• Recruit participants from HIV clinics where PrEP is available using PHRD recruitment strategies (e.g., workshops for healthcare workers).<br>• Strategies developed and refined during the pilot study.
**Referred peers**<br>(n=320)	• Age ≥16 to 24 years<br>• Female<br>• Referred by index peer to initiate PrEP.<br>• Able & willing to provide informed consent.	• Age <16 or >24 years<br>• Male<br>• Not referred by index peer to initiate PrEP<br>• Not able or willing to provide informed consent.	• Index peers will recruit referred peers. At point the of recruitment, referred peers will call research staff to provide contact info for follow-up.<br>• (IRB modification[1]): At follow-up, index peers share the contact information of peers referred so they can be reached for follow-up.<br>• If referred peer calls the study staff, both the referred peer and index peer will be compensated 150 KES[1] via mobile money.

* To minimize risks to minors, we will only enroll young women ≥16 to 17 years into the study if they are emancipated minors and thus able to legally provide consent for participation in research. Kenyan law acknowledges women ages 14 to 17 who have become pregnant as emancipated minors[36]

[1]This IRB modification was implemented after few referred peers contacted the research team to share their contract information. It was approved 6-months in to cRCT implementation in November 2023.

3.4. Study procedures

The overview of study procedures—including interventions and data collection activities—by study group is detailed in **Figure 2.**

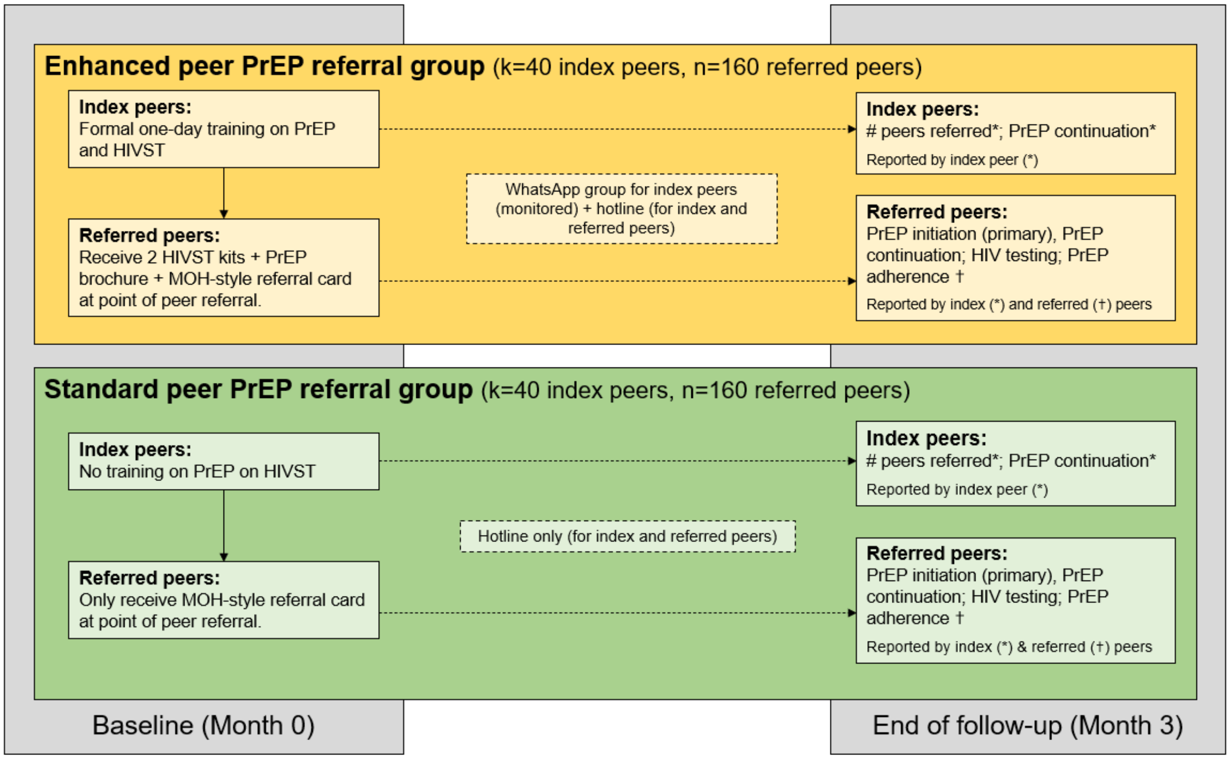

Figure 2. Overview of study procedures.

4. Study endpoints

We have registered all Peer PrEP cRCT outcomes on ClinicalTrials.gov (ID: NCT04982250) (2).

4.1. Primary & secondary PrEP outcomes

Our primary trial outcome will be PrEP initiation among referred peers, as reported by Index peers at the 3-month follow-up visit, **Table 3**. We will categorize clients as having initiated PrEP if they went to a healthcare facility (or other location) and were dispensed PrEP pills—consistent with how PrEP initiation is being measured in other PrEP implementation studies. We will also measure several secondary outcomes, including recent HIV testing, PrEP continuation, and PrEP adherence.

We are measuring all outcomes at 3 months to give referred peers time to both link to care, initiate PrEP services, and complete their first PrEP refill one month later if they are interested in continuing services. Additionally, this period is close enough to when the intervention was delivered that we anticipate little recall bias. Because we had challenges following up with referred peers in the pilot study leading up to this cRCT, we will be measuring all primary outcomes and most secondary outcomes reported by index peers, who are easier to follow-

up with and will report outcomes on behalf of the peers they recruited, as is typically done in interventions that utilize secondary distribution models. However, we will do our best to follow-up with referred peers and will measure our PrEP adherence outcomes reported from this population (we will also measure our primary and other secondary outcomes reported among this population and report them in sensitivity analyses).

Retention window (3 months): While most participants return for follow-up close to the scheduled 3-month visit date, operationally the scheduling window for this visit opens 2 weeks prior this scheduled visit date and closes 2 weeks after the scheduled date, unless a longer window was specifically requested by the participant. For our primary analysis, we will use follow-up visits assigned as 3-month visits by study staff.

Table 3. Detailed description of Peer PrEP cRCT primary and secondary outcomes

Outcome	Definition	Reported by	Timing
**PrEP initiation***	% of referred peers that went to a clinic and were **dispensed PrEP pills**, among those referred	Index peers*	Month 3
**Recent HIV testing**	% of referred peers who **tested for HIV** (any form of testing, including HIVST) since peer PrEP referral, among those referred.	Index peers	Month 3
**PrEP continuation**	% of referred peers that returned to a clinic to **refill PrEP**, among those referred.[1]	Index peers	Month 3
	% of index peers that returned to a clinic to **refill PrEP**, in the past 3 months (i.e., since peer referral)	Index peers	Month 3
**PrEP adherence**	Median of Wilson et al's 0-100 point adherence score,[2] with higher scores indicating higher adherence[1,3]	Referred peers	Month 3

* Primary outcome

[1]In prior versions of the SAP (v1.5), this was conditional among clients who initiated PrEP, but we changed to make it unconditional to align with the intent-to-treat analysis plan (described below) and best RCT practices on 07/11/2024—prior to study unblinding—per the recommendation of our DSMB.

[2]Wilson, I. B., et al. (2009). "Improving the self-report of HIV antiretroviral medication adherence: is the glass half full or half empty?" *Curr HIV/AIDS Rep* **6**(4): 177-186.

[3]In prior versions of this SAP (v1.4), we had suggested an adherence threshold based on the Wilson et al score, but on 3/22/2024—prior to study unblinding—we decided to turn this into a continuous outcome after observing low continuation and thus low adherence in the past month among referred peers in review of the aggregate data across study arms in our DSMB.

4.2. Process outcomes

We will also measure several process outcomes and compare these between the different study groups to help us better understand our primary and secondary outcomes. These process outcomes include: number of peers referred, HIVST use (enhanced group only), and

linkage to care among other relevant outcomes. The details for how we will measure these outcomes are described in **Table 4**; we will measure these outcomes among both index and referred, where appropriate.

Table 4. Detailed description of Peer PrEP cRCT process outcomes

Process outcomes	Definition	Reported by	Timing
**Peers referred**	Number **clients referred** to PrEP services by index peers	Index peers	Month 3
**HIVST use**	% of referred peers in the enhanced group that used an HIVST (once; twice; or for partner testing)	Referred peers	Month 3
**Client linkage to care**	% of referred peers that **went to a clinic** to access HIV prevention/treatment services, among those referred	Referred peers	Month 3

Note: All outcomes binary.

4.3. Implementation outcomes

We will measure acceptability, appropriateness, and feasibility of the PrEP referral + HIVST delivery model among index peers and referred peers at Month 3. To measure these outcomes, we will use questions based on validated theories and frameworks, including the Acceptability of Intervention Measure, Intervention Appropriateness Measure, and Feasibility of Intervention Measure (5, 6). See **Table 5** for more details on measurement of these outcomes.

Table 5. Detailed description of Peer PrEP cRCT implementation outcomes

Implementation outcome	Definition	Timing
**Acceptability**	% of referred peers & index peers who report our intervention model is **acceptable using validated scales**.	Months 3
**Appropriateness**	% of referred peers & index peers who report our intervention model is **appropriate using validated scales**.	Months 3
**Feasibility**	% of referred peers & index peers who report our intervention model is **feasible using validated scales**.	Months 3
**Fidelity**	% of referred peers reporting receiving the **different intervention materials** (e.g., educational brochure, 2 HIVST kits, and Kenya Ministry of Health referral card)	Month 3

5. Sample size justification

5.1. Sample size calculation

If we assume that index peers refer 75% of suggested referred peers, this leaves us with three clients per peer provider, or 240 referred peers in total. We performed power calculations for our primary outcome, the proportion of referred peers who initiated PrEP at three months, in Stata 16.1 using methods for cluster-randomized trials (cRCTs), where each cluster is a peer group of one index peer and three referred peers.

Table 6 and **Figure 3** shows the power we attain to detect various increases in the proportion of referred peers who initiated PrEP in the enhanced group versus the standard. For our power calculations, we assumed 40 clusters (index peers) per group, 3 sampling units (referred peers) per cluster, and an intra-cluster correlation coefficient (ICC) of 0.05, which is standard for cRCTs, we might have 80% power to detect a difference of 17% PrEP initiators between the enhanced group (80%) and standard group (63%).

Figure 3. Power calculation to detect increase in the percentage of referred peers initiating PrEP in the enhanced group compared to the standard.

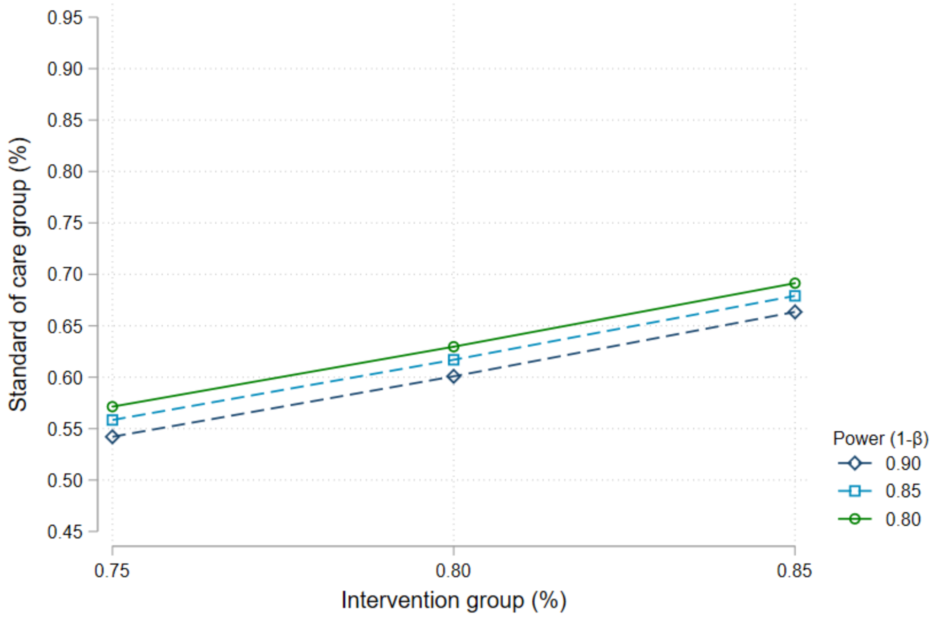

Table 6. Increase in the % of referred peers initiating PrEP in the enhanced group compared to standard under different assumptions.

Power	Standard: % PrEP initiation	Enhanced: % PrEP initiation	% increase
**80%**	57.1%	75.0%	17.7%
	63.0%	80.0%	17.0%
	69.1%	85.0%	15.7%
**85%**	55.8%	75.0%	19.2%
	61.7%	80.0%	18.3%
	67.9%	85.0%	17.10%
**90%**	54.2%	75.0%	20.8%
	60.1%	80.0%	19.9%
	66.4%	85.0%	18.7%

Assumptions: ICC: 0.05; clusters (providers) per arm: 40; units (clients) per cluster: 3 (75% of suggested clients referred)

6. Randomization and masking

6.1. Randomization

<u>*Randomization list.*</u> A Fred Hutch statistician will prepare a list of randomization assignments for index peers with block sizes of 4. This list will be stored in a password-protected electronic file on a Fred Hutch server.

<u>*Randomization implementation*</u>. Index peers will be randomized at enrollment, which will occur at Month 0. Then, referred peers will be chosen by index peers. At the time of randomization, index peers will open on opaque randomization envelope, given to them by a study research assistant, that has their study arm assignment inside.

7. Data collection

7.1. Database

All quantitative data will be collected electronically in face-to-face and remote interviews with trained Kenyan research assistants. We will use CommCare (Dimagi, Cambridge, USA), an electronic data collection platform, to collect the quantitative data and will upload this data to CommCare's secure server daily. A team of data experts in both Partners in Health and Research Development (PHRD) and Seattle (Fred Hutch) will monitor the data as it is coming in, and Seattle team will generate weekly data quality reports that will be shared with the PHRD team for review and feedback.

7.2. Participant identifiers

Participant identification numbers have the following format: 53-XX-YYY-Z, **Table 7.**

Table 7. Overview of participant identifiers in the Peer PrEP cRCT

Code	Definition
**53**	PHRD site code
**XX**	Specifies participant group:<br>• **03** = Index peer<br>• **04** = Referred peer
**YYY**	Sequential digits, specific to participant groups:
**Z**	Check digit, a random number: **1-9**

8. Statistical considerations

8.1. Analysis sets

<u>*Data sets*</u>. Data sets for analysis will be produced by Dr. Katrina Ortblad and Carlos Culquichicon. They will be .dta or .csv files containing a single header line whose variable names match those coded in CommCare. All missing values will be coded using "999". Codes for categorical variables (e.g., 0 for "No" and 1 for "Yes") will be used instead of character strings whenever possible.

Data codebook. A detailed codebook will be prepared, containing for each variable the form from which the variable derived, the text of the question, and all possible values for that variable with their coding. All codes and character strings representing categorical factors will be defined in the codebook.

9. Interim monitoring

The study will be monitored by a Data Scientific and Monitoring Board (DSMB) approximately every six months. The project coordinators (RM, AR, NW) and statistician (CC) will generate both an open and closed report (statistician only) that will be shared with the DSMB prior to the meeting. The DSMB will give recommendations based on the report and accompanying presentation, and all recommendations and meeting minutes will be reported to the UW and Kenyan IRBs.

10. Data analysis

10.1. Baseline characteristics

Baseline characteristics will be described by study group and population (e.g., index peers and referred peers). These will include demographics (e.g., gender, age, educational level), sexual behaviors (e.g., condom use), and behaviors associated with HIV risk, among other relevant characteristics.

10.2. Effect size estimation

Our main analysis will evaluate the difference in proportion of PrEP initiation between referred peers in the enhanced and standard groups, as reported by index peers at Month 3. Secondarily, among referred peers at Month 3, we will evaluate the difference in proportions for recent HIV testing (since referral) and PrEP continuation (i.e., PrEP refilling) and median difference for self-reported PrEP adherence (using 100-point validated scale).

Our analysis is prespecified as intention to treat (ITT), using complete-case information and standard errors clustered at the level of the peer provider. We will estimate risk differences (RD) with 95% confidence intervals (CI) for binary outcomes using mixed-effect models with Gaussian distributions, identity links, study group fixed effects, and index peer random effects to adjust for clustering. For continuous outcomes, we will estimate median differences (MDs) using linear quantile mixed methods with the same specifications as those described above.

All the analyses will consider an alpha level of 0.05 as significant and will be run in R Studio (RStudio: Integrated Development Environment for R. Posit Software, MA 2025).

10.3. Missing values and “don’t know” responses

Any missing observation due to data entry delays or CommCare skip logic conditions will be treated as failure. Similarly, any peer client’s outcomes reported by Index peers with “don’t know” responses, will be treated as failure.

10.4 Sensitivity analyses

- **Outcomes measured by referred peers (actual):** Measure effect estimate for peer client PrEP outcomes (e.g., initiation, recent HIV testing, continuation) as reported by referred peers, among those we were able to reach for follow-up.
- **Outcomes among all potential referred peers (n=320):** Measure effect estimates for referred peer PrEP outcomes (e.g., initiation, recent HIV testing, continuation) as reported by index peers, but among all potential referred peers index peers were recommended to recruit (n=320 clients, 4 per index peer)
- **Continuation among referred peers that initiated PrEP:** Measure effect estimates for peer client PrEP continuation as reported by index peers, among referred peers that initiated PrEP.
- **Treat "don't know" responses as successes.** Measure effect estimates for referred peer PrEP outcomes (e.g., initiation, recent HIV testing, continuation) reported by index peers, but assume all "don't know" responses are successes versus failures.
- **Multiple imputation of "don't know" responses** via Markov Chain Monte Carlo under the assumption of missingness at random.
- **GEE approach:** Measure effect size estimates for referred peer PrEP outcomes (e.g., initiation, recent HIV testing, continuation) as reported by peer provider using the generalized estimating equations.

10.5 Adverse events

The total number of adverse events will be reported by randomization group and severity. The proportion of participants with the following events will be compared using chi-square or Fisher's exact test:

- Physical violence, assault, or abuse
- Non-physical harassment or assault
- Unintentional or unauthorized disclosure of HIV status
- Suicidal thoughts or ideation
- Death

Serious adverse events (including any instance of violence, suicidality, or death) will be reported to the DSMB within 24 hours, including a report of the circumstances surrounding the adverse event. The randomization group of the participant will be communicated by the study statistician (CC) to the DSMB.

All serious and non-serious adverse events will be included in interim and final monitoring reports.

11. References

1. Ngure K, Ortblad KF, Mogere P, Bardon AR, Thomas KT, Mangale D, et al. Six-month PrEP dispensing with HIV self-testing to improve the efficiency of delivery in Kenya: a randomized non-inferiority implementation trial. Lancet HIV. 2022;In-press.

2. Ortblad KF. Peer PrEP Referral + HIV Self-test Delivery for PrEP Initiation in Kenya (PeerPrEP): ClinicalTrials.gov; 2023 [Available from: https://clinicaltrials.gov/ct2/show/NCT04982250?term=NCT04982250&draw=2&rank=1.

3. Zimet GD, Powell Ss Fau - Farley GK, Farley Gk Fau - Werkman S, Werkman S Fau - Berkoff KA, Berkoff KA. Psychometric characteristics of the Multidimensional Scale of Perceived Social Support. Journal of Personality Assessment. 1990;55(3):610-7.

4. Zimet G. Multidimensional Scale of Perceived Social Support (MSPSS). University of Miami; 2023.

5. Weiner BJ, Lewis CC, Stanick C, Powell BJ, Dorsey CN, Clary AS, et al. Psychometric assessment of three newly developed implementation outcome measures. Implementation Science. 2017;12(1):108.

6. Weiner BJ. Acceptability of Intervention Measure (AIM), Intervention Appropriateness Measure (IAM), & Feasibility of Intervention Measure. University of North Carolina in Chapel Hill (UNC); 2017.
