# Supplementary material for: Effect of enhanced peer PrEP referral with HIV self-testing delivery among young Kenyan women: A randomized controlled trial of peer networks
Source: PLoS Med. 2026 Mar 30;23(3):e1005023. doi: 10.1371/journal.pmed.1005023 (PMC13046272; doi:10.1371/journal.pmed.1005023)
Supplement: S1 Table — (DOCX) [file pmed.1005023.s005.docx]

| **S1 Table. Costing inputs that informed our unit cost estimates in the Ministry of Health scenario** | | |
| --- | --- | --- |
|  | **Enhanced peer referral** | **Standard peer referral** |
| **Cost Category** |  |  |
| Start-up costs^1^ | $550.06 | $183.35 |
| Staff training^2^ | $61.54 | $20.51 |
| Index peer interactions^3^ | $276.67 | $45.46 |
| Recurrent study supplies^4^ | $2,257.92 | $36.29 |
| *HIVST kits** | $1,152.00 | -- |
| Overhead^5^ | $13.68 | $7.36 |
| **Subtotal Cost** | **$3,159.87** | **$292.98** |
|  |  |  |
| **Among peers referred** | **n=137** | **n=104** |
| Cost per peer referred | $23 | $3 |
|  |  |  |
| **Among peers initiated** | **n=41** | **n=41** |
| Cost per peer initiated on PrEP | $77 | $7 |
| **Abbreviations:** HIVST (HIV self-testing); pre-exposure prophylaxis (PrEP).  ^1^ Example start-up costs: index peer training curriculum + materials development annualized over 5 years; creating staff training materials annualized over 5 years; communicating with and getting approval from nearby health facilities + transportation.  ^2^ Example staff training costs: laptop; projector; lunch; staff time to conduct training.  ^3^ Example index peer interaction costs: identifying index peers; scheduling and conducting index peer training sessions; monitoring WhatsApp group; supplying referral cards; maintaining patient logs.  ^4^ Example recurrent study supply costs: HIVST kits; referral cards + branded envelopes; brochures; other materials for enhanced referral package  ^5^ Example overhead costs: facilities and administrative costs (space, utilities)  **Excluded from subtotal costs because included in cost of recurrent study supplies* | | |
