# Supplementary material for: Effect of enhanced peer PrEP referral with HIV self-testing delivery among young Kenyan women: A randomized controlled trial of peer networks
Source: PLoS Med. 2026 Mar 30;23(3):e1005023. doi: 10.1371/journal.pmed.1005023 (PMC13046272; doi:10.1371/journal.pmed.1005023)
Supplement: S2 Table — (DOCX) [file pmed.1005023.s006.docx]

| **S2 Table. Sensitivity analyses for effect size estimates among referred peers reported by index peers (n) or referred peers self-reported (n*)** | | | | |
| --- | --- | --- | --- | --- |
|  | **Enhanced peer referral** | **Standard peer referral** | **Risk difference**  **[95% CI]** | **p-value** |
| **S1. Estimates among all potential referred peers^1^** | **n^1^=160** | **n^1^=168** |  |  |
| Any recent HIV testing | 125 (54%) | 56 (25%) | 30% [22%, 39%] | <0.001 |
| PrEP initiation | 41 (18%) | 41 (18%) | 3% [-4%, 10%] | 0.44 |
| PrEP continuation | 17 (7%) | 11 (5%) | 3% [-1%, 8%] | 0.17 |
|  |  |  |  |  |
| **S2. Continuation among those initiated PrEP^2^** | **n^2^=41** | **n^2^=41** |  |  |
| PrEP continuation | 17 (42%) | 11 (27%) | 10% [-16%, 36%] | 0.45 |
|  |  |  |  |  |
| **S3. Missing/‘don't know’ data = successes^3^** | **n=137** | **n=104** |  |  |
| Any recent HIV testing | 134 (98%) | 71 (68%) | 31% [17%, 44%] | <0.001 |
| PrEP initiation | 52 (38%) | 57 (55%) | -13% [-33%, 6%] | 0.18 |
| PrEP continuation | 126 (93%) | 83 (79%) | 11% [-1%, 24%] | 0.07 |
|  |  |  |  |  |
| **S4. Missing/‘don't know’ data: MCMC under MCAR^4^** | **n=137** | **n=104** |  |  |
| Any recent HIV testing | N/A | N/A | 35% [32%, 38%] | <0.001 |
| PrEP initiation | N/A | N/A | -11% [-14%, -8%] | 0.05 |
| PrEP continuation | N/A | N/A | 7% [-0.1%, 14%] | 0.30 |
|  |  |  |  |  |
| **S5. GEE** | **n=137** | **n=104** |  |  |
| Any recent HIV testing | 125 (91%) | 56 (54%) | 39% [23%, 55%] | <0.001 |
| PrEP initiation | 41 (30%) | 41 (41%) | -7% [-25%, 12%] | 0.49 |
| **S6. Removing referred peers using PrEP at referral^5^** | **n*=49** | **n*=22** |  |  |
| Any recent HIV testing | 48 (98%) | 12 (55%) | 43% [28%, 59%] | <0.001 |
| PrEP initiation | 8 (16%) | 8 (36%) | -26% [-52%, -1%] | 0.05 |
| PrEP continuation | 3 (6%) | 2 (9%) | -4% [-19%, 10%] | 0.57 |
| **Abbreviations:** confidence interval (CI); generalized estimating equations (GEE); markov chain monte carlo (MCMC); missingness completely at random (MCAR); pre-exposure prophylaxis (PrEP).  ^1^Outcomes reported among all potential referred peers if each index recruited the maximum four.  ^2^Outcomes reporting only among the subset of referred peers in each arm that initiated PrEP.  ^3^Categorized all missing outcomes as successes versus failures (i.e., how we pre-specified dealing with missing data in the primary and secondary analyses).  ^4^Bootstrapped estimates (percentile CIs from bootstrapped distribution).  ^5^Reported among referred peers reached for follow-up; not a pre-specified (i.e., a post hoc) sensitivity analysis. | | | | |
