# Supplementary material for: Effect of enhanced peer PrEP referral with HIV self-testing delivery among young Kenyan women: A randomized controlled trial of peer networks
Source: PLoS Med. 2026 Mar 30;23(3):e1005023. doi: 10.1371/journal.pmed.1005023 (PMC13046272; doi:10.1371/journal.pmed.1005023)
Supplement: S3 Table — (DOCX) [file pmed.1005023.s007.docx]

| **S3 Table. Features of index peers associated with PrEP initiation among referred peers, as reported by index peers (n)** | | | | |
| --- | --- | --- | --- | --- |
| **Index peer features** | **Initiated PrEP n=82** | **Did not initiate PrEP n=159** | **Risk difference^1^ [95% CI]** | **p-value** |
| Age; med (IQR) | 22 (20, 23) | 22 (21, 23) | 4% [-1%, 9%] | 0.09 |
| Married | 35 (43%) | 44 (28%) | 15% [-5%, 35%] | 0.15 |
| Any casual sexual partners | 19 (23%) | 38 (24%) | -2% [-25%, 21%] | 0.85 |
| Years of school; med (IQR) | 12 (12, 14) | 12 (10, 13) | -1% [-5%, 3%] | 0.53 |
| Currently in school | 11 (13%) | 39 (25%) | -16% [-39%, 7%] | 0.17 |
| Ever been pregnant | 56 (68%) | 113 (71%) | 2% [-19%, 23%] | 0.84 |
| STI diagnosis or treatment, *past 6 months* | 13 (16%) | 23 (14%) | 8% [-19%, 34%] | 0.57 |
| Engaged in transactional sex, *past 6 months* | 8 (10%) | 35 (22%) | -17% [-41%, 7%] | 0.17 |
| PrEP use: at the point of peer referral | 66 (80%) | 94 (59%) | 22% [3%, 42%] | 0.03 |

**Abbreviations:** interquartile range (IQR); risk difference (RD); pre-exposure prophylaxis (PrEP); sexually transmitted infections (STI).

^1^Risk differences calculated in bivariable regression models that controlled for study group and adjusted for clustering at the index peer level.
