# Supplementary material for: Effect of enhanced peer PrEP referral with HIV self-testing delivery among young Kenyan women: A randomized controlled trial of peer networks
Source: PLoS Med. 2026 Mar 30;23(3):e1005023. doi: 10.1371/journal.pmed.1005023 (PMC13046272; doi:10.1371/journal.pmed.1005023)
Supplement: Attachment — Submitted filename: Peer PrEP cRCT_RR_2024.10.17.docx [file pmed.1005023.s010.docx]

**RESPONSE TO REVIEWERS**

16th October 2025

ACADEMIC EDITOR:

- Consider a more descriptive study design for title. Although I understand the social network clusters are randomized, it does seem like this is somewhat more nuanced design with individual randomization of index peers and then assessing outcomes amongst their network.

**We updated the study title to clarify that the clusters randomized are peer networks. Additionally, we have updated our description of the study design in the Author Summary, Abstract, Introduction, and Methods to also clarify this.**

**(Title, Page 1, Lines 1-2):**

**“The effect of enhanced peer PrEP referral with HIV self-testing delivery among young Kenyan women: A randomized controlled trial of peer networks”**

**(Author Summary, Page 2, Lines 35-38):**

**“We conducted a two-arm randomized controlled trial of peer networks in which AGYW PrEP users (i.e., index peers) were encouraged to refer four peers to PrEP (i.e., referred peers) and randomized to either standard or enhanced peer referral inclusive of HIVST delivery.”**

**(Abstract, Page 4, Lines 58-60):**

**“Methods and Findings: We conducted a randomized controlled trial of peer networks (NCT04982250) in Central Kenya where AGYW (≥16-24 years) who used PrEP in the past 12 months (“index peers”) were randomized 1:1 to either: …”**

**(Introduction, Page 7, Lines 117-119):**

**“To test the intervention’s effectiveness on PrEP initiation compared to standard informal peer PrEP referral and generate evidence on its implementation that could inform scale-up, we conducted a hybrid randomized controlled trial of AGYW peer networks.”**

**(Methods, Page 7, Lines 122-125):**

**“The "Peer PrEP cRCT" was a hybrid type I effectiveness-implementation parallel cluster-level randomized superiority trial of AGYW peer networks (ClinicalTrials.gov: NCT04982250, registered: July 29, 2021) [18, 19]. Details of the trial protocol and statistical analysis plan (SAP) are described elsewhere (S1 Protocol and S2 SAP).”**

**(Methods, Pages 8-9, Lines 156-160):**

**“In this trial, index peers were randomized and outcomes assessed among referred peers—representing clusters of small AGYW peer networks. Index peers were randomized 1:1 to either: 1) *enhanced peer referral*, which included an in-person group training on PrEP referral strategies and HIV self-testing (HIVST) delivery (n=8 kits, 2 kits/peer), or 2) *standard peer referral,* which included informal word-of-mouth PrEP referral strategies.”**

- Some of the discussion of innovation of intervention may be better suited for discussion (after it is clear what the intervention are).

**We have revised the Introduction, moving discussion points on the intervention innovations to the Discussion section.**

**(Introduction, Pages 6-7, Lines 88-119):**

**“Adolescent girls and young women (AGYW, ≥16 to 24 years) in many African settings have high risk of HIV acquisition [1, 2] and low engagement in highly effective oral HIV pre-exposure prophylaxis (PrEP) services [3-5]. In Kenya, roughly a third of total new HIV infections are among AGYW who account for only ~10% of the population [6], and few AGYW (~5-15%) at public family planning clinics initiate PrEP when offered [5, 7]. Barriers to PrEP uptake among AGYW in Kenya and similar settings are multi-faceted and include individual-level barriers—such as lack of PrEP knowledge, uncertainty about PrEP efficacy and safety, and low HIV risk perception—as well as community-level barriers—such as stigma associated with PrEP use [5, 8, 9]. To address these barriers and reach more AGYW with PrEP, innovative PrEP referral and delivery strategies catered to this population are needed.**

**The opinion of peers often influences the health behaviors and preferences of populations with strong social networks, including AGYW. In Africa, most AGYW who initiate PrEP do so because of informal peer referral; in the HPTN 082 open-label PrEP study, 62% of 400 AGYW who initiated PrEP in South Africa and Zimbabwe did so due to peer encouragement [10]. Peer-delivered interventions have successfully increased engagement in HIV prevention services among other populations with strong social networks, including men who have sex with men [11] and female sex workers [12, 13], yet remain underexplored among AGYW. HIV prevention interventions that have engaged AGYWs in Africa to date have primary utilized “PrEP champions” [14], who engage in outreach activities to large peer networks, inclusive of individuals with whom they may or may not have personal connections. Peer delivery of HIV prevention interventions within small, established AGYW peer networks may enable more targeted outreach to individuals known to engage in behaviors associated with HIV risk acquisition who are most likely to benefit.**

**In collaboration with Kenyan stakeholders—including those from government, professional, and HIV implementing organizations, as well as AGYW—we developed a peer-delivered HIV prevention intervention for small AGYW peer networks that included formal training on PrEP referral strategies and HIV self-testing (HIVST) delivery [15]. This intervention was informed by formative qualitative research—which demonstrated high perceived intervention acceptability among Kenyan AGYW and stakeholders [15, 16], and pilot testing—which demonstrated feasibility [17]. To test the intervention’s effectiveness on PrEP initiation compared to standard informal peer PrEP referral and generate evidence on its implementation that could inform scale-up, we conducted a hybrid randomized controlled trial of AGYW peer networks.”**

**(Discussion, Pages 20-21, Lines 373-384)**

**“In this intervention, the use of HIVST to promote linkage to prevention services was novel, as most of HIVST interventions to date have primary focused on identifying individuals living with HIV and facilitating linkage to treatment services [38-41]. However, most individuals who complete HIVST at home will test negative and may feel motivated to change their behavior and/or engage in HIV prevention interventions to maintain their HIV negative status [42, 43]. Consistent with other peer-delivered HIVST interventions in populations with strong social networks [11-13], this intervention significantly increased HIV testing uptake among AGYW. The additional index peer training on PrEP referral strategies, however, was not enough to translate HIV testing increases into PrEP initiation increases compared to informal peer referral. For AGYW in the enhanced group, barriers to clinic-based PrEP remained; while for AGYW in the standard group, many of the barriers they had to overcome for clinic-based testing were similar to those for clinic-based PrEP, facilitating same-day PrEP initiation.”**

- Were there any positive HIV tests?

**In both the Abstract and Results we report that according to index peers, three referred peers tested HIV-positive.**

**(Abstract, Page 4-5, Lines 75-78):**

**“Recent HIV testing, however, was significantly higher among referred peers in the enhanced (91%, 125/137) versus standard (54%, 56/104) group (RD 39%, 95% CI [24%, 54%], p<0.001); three referred peers, all in the enhanced group, tested HIV-positive.”**

**(Results, Page 16, Lines 318-319):**

**“According to index peers, three referred peers tested HIV-positive: all in the enhanced group.”**

- Were features of index peers or their referrals associated with better outcomes?

**To understand features of index peers associated with our primary PrEP initiation outcome, we added an exploratory post hoc analysis, which we now describe in the Methods and report findings of in the Results. We did not explore features of referred peers associated with this outcome because index peers only reported limited demographic information on the peers they referred—a limitation of this study that we mention in the Discussion.**

**(Methods, Page 13, Lines 270-273):**

**“As a post hoc exploratory analysis, we conducted bivariable regression models that controlled for study group and adjusted for clustering at the index peer level to understand features of index peers associated with PrEP initiation among referred peers (population a). Features associated with the outcome at the alpha=0.05 level were considered statistically significant.”**

**(Results, Page 16, Lines 320-323):**

**“In our exploratory analysis, PrEP use among index peers at the point of peer referral was significantly associated with increased PrEP initiation among referred peers (RD 22%, 95% CI [3%, 42%], p=0.03), S5 Table; no other index peer features were statistically associated with this outcome.”**

**(Supplementary files, S5 Table):**

REVIEWER #1: STATISTICAL REVIEWER

This paper reports a cluster-randomized trial comparing approaches to utilizing peers to encourage people to engage with pre-exposure preventative treatment for HIV in Kenya. Overall, I thought the trial was well reported. I have some relatively minor comments on the statistical aspects below.

1. (Abstract): I would recommend that (for at least the primary outcome), that a p-value is given in addition to the confidence interval, given the trial was prospectively powered to test that outcome's hypothesis. Personally I'd also provide p-values for secondary endpoints (especially as one of them has a significance claim provided) but the authors may feel that this is less appropriate.

**We have added p-values for all the effect estimates, including those for primary and secondary outcomes, reported in our Abstract.**

**(Abstract, Page 4, Lines 71-78):**

**“At follow up, there were no statistically significant differences between study groups in PrEP initiation (enhanced: 30%, 41/137; standard: 41%, 41/104; RD -6%, 95% CI [-26%, 11%], p=0.51) nor PrEP continuation (enhanced: 13%, 17/137; standard: 11%, 11/104; RD 1%, 95% CI [-10%, 13%], p=0.82) among referred peers. Recent HIV testing, however, was significantly higher among referred peers in the enhanced (91%, 125/137) versus standard (54%, 56/104) group (RD 39%, 95% CI [24%, 54%], p<0.001); three referred peers, all in the enhanced group, tested HIV-positive.”**

2. (Page 7): "The trial protocol and statistical analysis plan can be found on the trial registration website"—I am not sure if these are separate documents but I could not see the SAP provided on CT.gov (there is a protocol and informed consent form listed at the bottom of the page). Is there an SAP that could be provided as a supplementary file?

**We updated and finalized the CT.gov record for this study (NCT04982250) on August 8, 2025, potentially after this review was completed. Finalizing this record took longer than anticipated because we had several back and fourths with CT.gov reviewers to identify how to best report the outcomes for our various study populations of interest—e.g., referred peers reported by index peer, referred peers self-reported, and index peers self-reported. The study protocol and final SAP are now available for download via CT.gov (see screenshot below) and as Supplementary Files with this resubmission.**

**(CT.gov, Screenshot on 9/17/25):**

**(Methods, Page 7, Lines 124-125):**

**“Details of the trial protocol and statistical analysis plan (SAP) are described elsewhere (S1 Protocol and S2 SAP).”**

3. (Page 11): I did not find it clear what the k, n, n* notation meant (until later in the results). Perhaps it could be briefly clarified that this the size of the population being referred to?

**We have revised this paragraph and introduced new notation—separate from that used to represent sample size in our Tables/Figures—to help clarify the population among which we are measuring study outcome.**

**(Methods, Pages 11-12, Lines 220-232):**

**“We report outcomes at follow-up for three study populations: a) referred peers reported by index peers (in index peer follow-up surveys); b) referred peers self-reported (in referred peer follow-up surveys), and c) index peer self-reported (in index peer follow-up surveys). Our prespecified primary outcome was PrEP initiation among referred peers reported by index peers (population a), defined as visiting a clinic or other location and being dispensed PrEP. Prespecified secondary outcomes included recent HIV testing (any form following peer referral) and PrEP continuation (refilling one month following initiation) among referred peers reported by index peers (population a), PrEP adherence (past month) among referred peers (population b), and PrEP continuation (refilling following enrollment) among index peers (population c). To asses PrEP adherence in the past month we used a validated three-item 100-point scale [18], with higher scores indicating better adherence and zero indicating no PrEP use. Additional prespecified outcomes included HIV testing, PrEP initiation, and PrEP continuation among referred peers (population b) and the number of peers referred by each index (population c).”**

4. (Page 11): there are several endpoints mentioned on the clinicaltrials.gov page which I didn't see reported here (e.g. social support, PrEP knowledge). I would recommend that the paper mentions all outcomes in the clinicaltrials.gov page, even if they are not reported here.

**As discussed above, our CT.gov record was last updated on August 8, 2025, potentially after this review was completed. The current CT.gov record aligns with the final SAP (which was finalized prior to any analysis of cRCT data), the primary and secondary outcomes in the published Peer PrEP cRCT protocol paper (**[**https://pubmed.ncbi.nlm.nih.gov/37925450/**](https://pubmed.ncbi.nlm.nih.gov/37925450/)**), and the primary and secondary outcomes listed in the Methods of this manuscript.**

**(CT.gov, Screenshot on 9/17/25):**

**(Methods, Pages 11-12, Lines 220-234):**

**“We report outcomes at follow-up for three study populations: a) referred peers reported by index peers (in index peer follow-up surveys); b) referred peers self-reported (in referred peer follow-up surveys), and c) index peer self-reported (in index peer follow-up surveys). Our prespecified primary outcome was PrEP initiation among referred peers reported by index peers (population a), defined as visiting a clinic or other location and being dispensed PrEP. Prespecified secondary outcomes included recent HIV testing (any form following peer referral) and PrEP continuation (refilling one month following initiation) among referred peers reported by index peers (population a), PrEP adherence (past month) among referred peers (population b), and PrEP continuation (refilling following enrollment) among index peers (population c). To assess PrEP adherence in the past month we used a validated three-item 100-point scale [25], with higher scores indicating better adherence and zero indicating no PrEP use. Additional prespecified outcomes included HIV testing, PrEP initiation, and PrEP continuation among referred peers (population b) and the number of peers referred by each index peer (population c). A post-hoc outcome was partner HIV testing following referral among referred peers (population b), which we added to help understand our null primary finding.”**

5. (Page 12): ‘identify links' should be 'identity link function'.

**We have updated “identify links” to “identity link functions.”.**

**(Methods, Page 13, Lines 257-259):**

**“Risk differences (RD) with 95% confidence intervals (CI) were estimated for all binary outcomes using regression models with Gaussian distributions, identity link functions, study group fixed effects, and index peer random effects (to adjust for clustering).”**

6. (Page 13): I would have welcomed more mention of the reasons for the big difference between n and n*—is it definitely confirmed that referred peers as reported by the index peers are genuine? Where the referred peers followed-up with, did information reported by the index peer generally agree with the information reported by the referred peer?

**In the Results, we have clarified some of the challenges with reaching referred peers for follow-up that resulted in the big differences between n and n*. Additionally, in the Discussion we discuss how relying on index peers to report outcomes for referred peers was a study limitation.**

**(Results, Page 15, Lines 299-306):**

**“Over the trial duration, we obtained contact information for 60% (145/241) of the referred peers, 116 (48%) of whom we reached for follow-up and 83 (34%) of whom we enrolled from November 21, 2023 to August 27, 2024. We encountered many challenges reaching referred peers for follow-up associated with few calling us at the point of referral (prior to an IRB modification that enabled us to contact them directly) and few answering their phone when we attempted to reach them. Common reasons for exclusion among those reached were age ineligibility (52%, 17/33) and disinterest in study participation (36%, 12/33). All enrolled referred peers were unique individuals; 55 in the enhanced group and 28 in the standard.”**

**(Discussion, Page 22, Lines 408-416):**

**“Another weakness included relying on index peers to report characteristics and outcomes for referred peers, limiting our ability to describe the population referred and subjecting our outcomes to potential missingness, recall bias, and falsification. Despite the limitations of this approach, having index participants report outcomes for recruited participants is common practice in assessment of peer-delivered interventions [44, 45]. While our study findings remained consistent among the referred peers reached for follow-up, we encountered challenges reaching this population, which resulted in high and differential loss-to-follow-up by study group (60% in the enhanced and 73% in the standard) that may have biased our secondary outcomes and effect estimates.”**

**In Table 2, we present the effect size estimates for study outcome among referred peers self-reported, which were consistent with those among referred peers reported by index peers (reported in Figure 2).**

**(Results, Page 17, Table 2):**

7. (Table 2): As per my comment on the abstract, I feel p-values are appropriate to add to results, especially as the statistical section refers to significance level.

**We have added p-values to Table 2, in which we report the effect of enhanced versus standard PrEP peer referral on secondary study outcomes. Additionally, we have added p-values to Figure 2, in which we present the effect of enhanced vs. standard peer PrEP referral on the primary and key secondary outcomes, as well as to S4 Table, in which we report the findings from our sensitivity analyses.**

**(Results, Page 17, Table 2): [See above]**

**(Results, Figure 2):**

**(Supplementary files, S4 Table):**

8. (Results): I would recommend reporting the estimated ICC, as this could be useful for future studies.

**We have estimated the ICC for the primary study outcome—PrEP initiation among referred peers reported by index peers—and now report this in the Results. Additionally, in the Discussion we now mention as a limitation the high observed ICC, which limited our power to detect differences in PrEP initiation between the study groups.**

**(Results, Page 16, Lines 313-315):**

**“PrEP initiation among referred peers was 30% (41/137) in the enhanced group and 41% (41/104) in the standard (RD -6%, 95% CI [-26%, 11%], p=0.51; estimated ICC 0.66), while PrEP continuation was ….”**

**(Discussion, Page 22, Lines 418-420):**

**“Finally, the ICC observed in this study (0.66) was much higher than that anticipated in the design (0.05), which lowered our power to observe significant differences in PrEP initiation between study groups.”**

9. (Page 19): The last paragraph implies that the rate of PrEP initiation was higher than expected. Unless I misunderstood, it was considerably lower than what was anticipated in the power calculation, so I didn't follow this statement.

**We have significantly revised the Discussion of this manuscript and now discuss this point in the context of our study conclusions, where we indicate that the high PrEP initiation observed in the standard group was consistent with our pre-trial assumptions.**

**(Discussion, Pages 22-23, Lines 421-429):**

**“As we enter a new era of constrained funding for HIV programming, low-cost implementation strategies that can target intervention delivery to those who could benefit most are needed [46]. Consistent with our pre-trial assumption, standard informal PrEP referral within small peer networks resulted in high PrEP initiation (41%)—much higher than that observed at family planning clinics in Kenya (4-16% [5, 7]), suggesting that peer delivery of MOH PrEP referral cards alone—a light touch intervention—might be effective for engaging AGYW in PrEP. While peer PrEP referral enhanced with training and HIVST delivery did not increase PrEP initiation compared to the standard, it did increase HIV testing—the first step of the HIV prevention and treatment cascade.”**

REVIEWER #2:

This cluster-randomized controlled trial assesses the effects of enhanced peer referral compared to standard peer referral on PrEP initiation among adolescent girls and young women in Kenya. This study aims to address an important public health challenge associated with low PrEP uptake and continuation among a group disproportionately impacted by HIV. I have some comments as follows.

*Abstract*

1. (Line 71): Specify timeframe for the PrEP continuation (i.e., month 1 refill).

**We have specified the timeframe for PrEP continuation in the Abstract and Author Summary.**

**(Abstract, Page 4, Lines 65-67):**

**“Index peers were followed up three months later and reported outcomes for referred peers: PrEP initiation (primary), PrEP continuation (i.e., month one refills), and HIV testing (any form following referral).”**

**(Author Summary, Page 2, Lines 39-42):**

**“Three months following referral, there were no differences in PrEP initiation or continuation (i.e., month one refills) among referred peers that received standard versus enhanced referral, but recent HIV testing (any form following referral) was significantly higher among those who received enhanced referral.”**

*Methods*

1. (Line 118 and throughout the text): Specify type of hybrid design (type 1?).

**We have specified in the Methods that this was a hybrid type I trial.**

**(Methods, Page 7, Lines 122-124):**

**“The "Peer PrEP cRCT" was a hybrid type I effectiveness-implementation parallel cluster-level randomized superiority trial of AGYW peer networks (ClinicalTrials.gov: NCT04982250, registered: July 29, 2021) [18, 19].”**

2. (Lines 122-123): "We conducted this trial in three counties in Central Kenya (Kiambu, Nairobi, and Murang'a) where the population-level HIV prevalence is ~2% [22]." Contrast this data with the national estimate to provide further background information to the readers.

**We now clarify in the Methods how this population-level HIV prevalence compares with the national average.**

**(Methods, Page 7, Lines 125-127):**

**“We conducted the trial in three counties in Central Kenya (Kiambu, Nairobi, and Murang’a) where the population-level HIV prevalence is ~2-4%, slightly lower than the national average of ~4% [20-22].”**

3. Specify how many facilities were included to review the records of public clinics.

**We have clarified in the Methods the number of PrEP clinics and programs we reviewed the records of to identify AGYW who met the trial criteria.**

**(Methods, Page 8, Lines 135-137):**

**“To recruit index peers, we reviewed the records of 73 public clinics and four research programs delivering PrEP in the region and called AGYW who met the trial criteria.”**

4. (Lines 169-170): "Research staff experienced in PrEP implementation and AGYW engagement led these one-day trainings of 4-8 index peers…". Can you specify if this one-day training was the only training received? Any additional mentoring or refresher training conducted during implementation?

**We have clarified in the Methods that the training index peers in the intervention group received was a one-time training and that no additional mentoring or refresher trainings were conducted over the implementation period.**

**(Methods, Page 9, Lines 174-175):**

**“Enhanced peer referral. Index peers assigned to this study group were scheduled to complete a one-time group training on PrEP referral strategies.”**

**(Methods, Page 10, Lines 192-193):**

**“Beyond the WhatsApp group support, index peers received no additional mentoring or refresher trainings.”**

5. How long was the training delivered to the peers assigned to the control group?

**In the Results, we have clarified the duration of training delivered to index peers in both the intervention and control groups—as measured using data from our time-and-motion observations.**

**(Results, Page 19, Lines 354-356):**

**“Time-and-motion observations revealed that it took ~4 hours to train index peers on PrEP referral in the enhanced group and ~8 minutes to encourage index peers to refer peers to PrEP in the standard.”**

6. (Lines 213): For HIV testing, I am assuming this refers to HIV testing at the clinic and not HIVST. Please specify in the text.

**We have clarified that our HIV testing outcome is inclusive of all HIV testing forms, including clinic-based testing and at-home HIV self-testing, in both the Abstract and Methods.**

**(Abstract, Page 4, Lines 65-68):**

**“Index peers were followed up three months later and reported outcomes for referred peers: PrEP initiation (primary), PrEP continuation (i.e., month one refills), and HIV testing (any form following referral).”**

**(Methods, Page 11, Lines 224-228):**

**“Prespecified secondary outcomes included recent HIV testing (any form following peer referral) and PrEP continuation (refilling one month following initiation) among referred peers reported by index peers (population a), PrEP adherence (past month) among referred peers (population b), and PrEP continuation (refilling following enrollment) among index peers (population c).”**

7. (Line 237): Provide justification or citation for the intra-cluster correlation value.

**We have clarified that an ICC of 0.05—which was used for the sample size calculation for this trial—is typical for cluster-randomized controlled trials. We have also added a citation to justify this statement.**

**(Methods, Page 12, Lines 250-254):**

**“With 80 clusters (40 per study group) of one index peer and three referred peers (75% of those recommended), we had 80% power in this trial to detect a 17% difference in PrEP initiation between the standard (63%) and enhanced (80%) groups, assuming an intra-cluster correlation coefficient (ICC) of 0.05—typical for cluster-randomized controlled trials [30]—and an alpha level of 0.05.”**

**Additionally, we now report the ICC observed in this study in the Results and discuss**

**this high value as a study weakness in the Discussion.**

**(Results, Page 16, Lines 313-315):**

**“PrEP initiation among referred peers was 30% (41/137) in the enhanced group and 41% (41/104) in the standard (RD -6%, 95% CI [-26%, 11%], p=0.51; estimated ICC 0.66), while PrEP continuation was ….”**

**(Discussion, Page 22, Lines 418-420):**

**“Finally, the ICC observed in this study (0.66) was much higher than that anticipated in the design (0.05), which lowered our power to observe significant differences in PrEP initiation between study groups.”**

8. For referred peers, it would be helpful if the authors specify how they confirm the identity of the referred peers to prevent being referred twice (and counted twice) by different index peers.

**In the Methods, we have clarified that index peers were instructed to not refer peers to PrEP already referred by another index peer. Additionally in this section, we discuss how the Kenya study team carefully monitored the contact information of referred peers received to help ensure there were no duplicates (suggesting referral of the same peer by more than one index). In the Results, we have clarified that all referred peers enrolled in the study were unique participants, and in the Discussion, we now list our reliance on index peers to recruit eligible and unique referred peers as study limitation.**

**(Methods, Page 8, Lines 137-144):**

**“Enrolled index peers were then instructed to recruit female peers aged 16-24 years who were PrEP-naïve or had not used PrEP in the past three months, and had not been recruited by another index peer... The Kenya study team carefully monitored the contact information of referred peers received to prevent double enrollment of the same referred peer by more than one index.”**

**(Results, Page 15, Lines 299-306):**

**“Over the trial duration, we obtained contact information for 60% (145/241) of the referred peers, 116 (48%) of whom we reached for follow-up and 83 (34%) of whom we enrolled from November 21, 2023 to August 27, 2024. We encountered many challenges reaching referred peers for follow-up associated with few calling us at the point of referral (prior to an IRB modification that enabled us to contact them directly) and few answering their phone when we attempted to reach them. Common reasons for exclusion among those reached were age ineligibility (52%, 17/33) and disinterest in study participation (36%, 12/33). All enrolled referred peers were unique individuals; 55 in the enhanced group and 28 in the standard.”**

**(Discussion, Page 22, Lines 406-407):**

**“Weaknesses included relying on index peers to recruit unique and eligible referred peers, risking potential contamination between study groups—which we monitored for and did not observe.”**

9. Include brief information about the sample size calculation.

**In the Methods, we have added more information about the sample size calculation, including findings from a pilot study of the enhanced peer PrEP referral model that informed this calculation.**

**(Methods, Page 12, Lines 248-254):**

**“The sample size for this trial was based on the primary PrEP initiation outcome and informed by findings from a pilot study of the enhanced peer referral model conducted in the same study setting (in which PrEP initiation among referred peers was 78%, 43/55) [29]. With 80 clusters (40 per study group) of one index peer and three referred peers (75% of those recommended), we had 80% power in this trial to detect a 17% difference in PrEP initiation between the standard (63%) and enhanced (80%) groups, assuming an intra-cluster correlation coefficient (ICC) of 0.05—typical for cluster-randomized controlled trials [30]—and an alpha level of 0.05.”**

*Discussion*

1. (Lines 331): Specify period for PrEP continuation (e.g., at month 1).

**In the Discussion, we have clarified that the intervention did not significantly increase PrEP continuation one month following initiation. In the Methods, we have also added clarification around the timing of this outcome.**

**(Discussion, Page 20, Lines 364-367):**

**“Among Kenyan AGYW at risk of HIV acquisition, enhanced peer PrEP referral supported with training and HIVST delivery did not significantly increase PrEP initiation and one-month continuation compared to standard, word-of-mouth peer referral, but did significantly increase recent HIV testing.”**

**(Methods, Page 11, Lines 224-228):**

**“Prespecified secondary outcomes included recent HIV testing (any form following peer referral) and PrEP continuation (refilling one month following initiation) among referred peers reported by index peers (population a), PrEP adherence (past month) among referred peers (population b), and PrEP continuation (refilling following enrollment) among index peers (population c).”**

2. The discussion could be strengthened by contextualizing the findings with the broader scientific literature.

**We have significantly revised the Discussion to better contextualize our findings within the context of the broader scientific literature on HIVST, partner testing, peer-delivered interventions, and PrEP initiation among AGYW in Kenya.**

**(Discussion, Pages 20-21, Lines 373-393):**

**“In this intervention, the use of HIVST to promote linkage to prevention services was novel, as most of HIVST interventions to date have primary focused on identifying individuals living with HIV and facilitating linkage to treatment services [38-41]. However, most individuals who complete HIVST at home will test negative and may feel motivated to change their behavior and/or engage in HIV prevention interventions to maintain their HIV negative status [42, 43]. Consistent with other peer-delivered HIVST interventions in populations with strong social networks [11-13], this intervention significantly increased HIV testing uptake among AGYW. The additional index peer training on PrEP referral strategies, however, was not enough to translate HIV testing increases into PrEP initiation increases compared to informal peer referral. For AGYW in the enhanced group, barriers to clinic-based PrEP remained; while for AGYW in the standard group, many of the barriers they had to overcome for clinic-based testing were similar to those for clinic-based PrEP, facilitating same-day PrEP initiation.**

**Differences in partner HIV testing among referred peers in the two study groups might have contributed to our null PrEP initiation findings. In the enhanced group, nearly half of referred peers reported using their second HIVST kit for partner testing (42%), which may have driven the higher but not statistically significant uptake of partner testing following peer referral in the enhanced group (46%) compared to the standard (35%). These findings would be consistent with other studies in Kenya and Malawi that demonstrated delivery of multiple HIVST kits to pregnant women significantly increased male partner HIV testing [44, 45]. In this study, most referred peers who engaged in partner HIV testing learned their partner was not living with HIV, which may have demotivated their PrEP interest.”**

**(Discussion, Page 22, Lines 408-412):**

**“Another weakness included relying on index peers to report characteristics and outcomes for referred peers, limiting our ability to describe the population referred and subjecting our outcomes to potential missingness, recall bias, and falsification. Despite the limitations of this approach, having index participants report outcomes for recruited participants is common practice in assessment of peer-delivered interventions [44, 45]..”**

**(Discussion, Page 22, Lines 423-426):**

**“Consistent with our pre-trial assumption, standard informal PrEP referral within small peer networks resulted in high PrEP initiation (41%)—much higher than that observed at family planning clinics in Kenya (4-16% [5, 7]), suggesting that peer delivery of MOH PrEP referral cards alone—a light touch intervention—might be effective for engaging AGYW in PrEP.”**

3. Some statements appear too strong based on the data presented. For instance, "For example, among referred peers reached for follow-up, a greater percentage in the standard group reported an STI diagnosis (25%) and engagement in transactional sex (25%) in the past six months compared to those in the enhanced (9% and 13%, respectively)." These percentages are based on small absolute numbers; STI diagnosis is 5 (9%) and 7 (25%). I suggest deleting this statement as misleading when taking into consideration the small absolute number.

**We have deleted this statement and ensured statements made throughout the Discussion align with the strength of the data presented in the Results. The paragraph that discusses how the intervention might have contributed to different types of peers referred has been updated to the following:**

**(Discussion, Page 21, Lines 394-402):**

**“The null findings in this study might also be attributable to the types of peers referred in the enhanced group compared to the standard. While index peers in the standard group referred fewer, but not statistically significant fewer, peers to PrEP than those in the enhanced, the risk of HIV acquisition among those referred might have been greater in the standard group compared to the enhanced (which was challenging to assess in this study due to limited collected data on referred peer characteristics). It is possible that with the training and HIVST kits, index peers in the enhanced group felt extra motivation or pressure to refer four total peers to PrEP, including some who might not have benefited; potentially driving the proportion of referred peers that initiated PrEP in this group lower than in the standard.”**

4. The author should expand on the limitations and also their potential effects on the estimates. Limitations of the study include a short follow-up period of only 1 month for PrEP refill (month 1), data was available only on 60% of the referred peers, and reliance on self-reported data, and most data were reported by index peers.

**We have expanded on the study limitations—including all those listed by the reviewer in this comment—and now describe the potential effects these had on our effect size estimates.**

**(Discussion, Page 22, Lines 406-420):**

**“Weaknesses included relying on index peers to recruit unique and eligible referred peers, risking potential contamination between study groups—which we monitored for and did not observe. Another weakness included relying on index peers to report characteristics and outcomes for referred peers, limiting our ability to describe the population referred and subjecting our outcomes to potential missingness, recall bias, and falsification. Despite the limitations of this approach, having index participants report outcomes for recruited participants is common practice in assessment of peer-delivered interventions [44, 45]. While our study findings remained consistent among the referred peers reached for follow-up, we encountered challenges reaching this population, which resulted in high and differential loss-to-follow-up by study group (60% in the enhanced and 73% in the standard) that may have biased our secondary outcomes and effect estimates. The relatively short follow-up period (three months) was another study weakness, as this covered only one PrEP refill opportunity and prevented assessment of the intervention’s impact on referred peers’ participation in HIV prevention services over time. Finally, the ICC observed in this study (0.66) was much higher than that anticipated in the design (0.05), which lowered our power to observe significant differences in PrEP initiation between the study groups.”**

5. (Lines 372): Should add that the follow-up period includes only 1 PrEP refill (month 1). For example, "…and the relatively short follow-up period for all 372 study participants (three months covering month 1 PrEP refill only).

**We have clarified in the Discussion that the relatively short follow-up period only covers one PrEP refill opportunity. The timing of this refill has been clarified in the Methods.**

**(Discussion, Page 22, Lines 416-418):**

**“The relatively short follow-up period (three months) was another study weakness, as this covered only one PrEP refill opportunity and prevented assessment of the intervention’s impact on referred peers’ participation in HIV prevention services over time.”**

**(Methods, Page 11, Lines 224-228):**

**“Prespecified secondary outcomes included recent HIV testing (any form following peer referral) and PrEP continuation (refilling one month following initiation) among referred peers reported by index peers (population a), PrEP adherence (past month) among referred peers (population b), and PrEP continuation (refilling following enrollment) among index peers (population c).”**

6. (Line 341): Missing a number after "was".

**We have deleted this sentence in response to other comments.**

JOURNAL REQUIREMENTS:

1. Please provide a completed 'Competing Interests' statement, including any COIs declared by your co-authors. If you have no competing interests to declare, please state "The authors have declared that no competing interests exist". Otherwise please declare all competing interests beginning with the statement "I have read the journal's policy and the authors of this manuscript have the following competing interests:"

**In our Declarations we have provided a “Competing Interests” statement where we declare the authors have no competing interests.**

**(Declarations, Page 24, Lines 443-444):**

**“The authors have declared that no competing interests exist. JMB is an employee of Gilead Sciences, outside the present work.”**

2. We have noticed that you have uploaded Supporting Information files, but you have not included a list of legends. Please add a full list of legends for your Supporting Information files after the references list.

**Following the Declarations, we now include a list of legends for our Supporting Information (Supplementary) files.**

3. Please note that the contact for accessing your data cannot be an author on the manuscript. Please therefore replace the current contact information with a third party contact, such as an institutional data access committee.

**We obtained IRB approval to share this data in a public repository. We have uploaded the deidentified data, data dictionary, and analytic code for the analyses in this paper to GitHub. We’ve updated the Declarations for data sharing to reflect this.**

**(Declarations, Page 24, Lines 446-447):**

**“Deidentified data, a supporting data dictionary, and statistical code for the analyses in this paper are publicly available at** [**https://github.com/Peer-PrEP-trial-Hutch/Peer-PrEP-main-manuscript**](https://github.com/Peer-PrEP-trial-Hutch/Peer-PrEP-main-manuscript)**.”**

4. Some material included in your submission may be copyrighted. According to PLOS’s copyright policy, authors who use figures or other material (e.g., graphics, clipart, maps) from another author or copyright holder must demonstrate or obtain permission to publish this material under the Creative Commons Attribution 4.0 International (CC BY 4.0) License used by PLOS journals. Please closely review the details of PLOS’s copyright requirements here: PLOS Licenses and Copyright. If you need to request permissions from a copyright holder, you may use PLOS's Copyright Content Permission form.

*Potential Copyright Issues:*

i. Figures 1,2,3, and 8 in “Peer PrEP referral Protocol_Version 1.11 (clean)_IRB stamped.pdf”: Please confirm whether you drew the images/clip-art within the figure panels by hand. If you did not draw the images, please provide (a) a link to the source of the images or icons and their license / terms of use; or (b) written permission from the copyright holder to publish the images or icons under our CC BY 4.0 license. Alternatively, you may replace the images with open-source alternatives.

**We have updated these figures so that they no longer include images/clip-art. The Study Protocol with updated images that align with PLOS’s copyright requirements has been included as a Supplemental File S1 Protocol.**

**(Study Protocol, Figure 1):**

**(Study Protocol, Figure 2):**

**(Study Protocol, Figure 3):**

ii. Figure 4 in “Peer PrEP referral Protocol_Version 1.11 (clean)_IRB stamped.pdf”: please (a) provide a direct link to the base layer of the map (i.e., the country or region border shape) and ensure this is also included in the figure legend; and (b) provide a link to the terms of use / license information for the base layer image or shapefile.

**In our updated Study Protocol (S1 Protocol) we have deleted this image of the map that was not aligned with PLOS’s copyright requirements.**

iii. Figure 6 in “Peer PrEP referral Protocol_Version 1.11 (clean)_IRB stamped.pdf”: Please confirm (a) that you are the photographer; or (b) provide written permission from the photographer to publish the photo(s) under our CC BY 4.0 license.

**We have removed this figure from the protocol included with this resubmission (S1 Protocol) to comply with PLOS Medicine’s copywrite guidelines.**

GENERAL EDITORAL REQUESTS:

- We ask every co-author listed on the manuscript to fill in a contributing author statement, making sure to declare all competing interests. If any of the co-authors have not filled in the statement, we will remind them to do so when the paper is revised.

**We will remind co-authors to complete a contributing author statement in which they declare all competing interests.**

- Please upload any figures associated with your paper as individual TIF or EPS files with 300dpi resolution at resubmission.

**In this resubmission, we have used PLOS’s NAAS tool and ensured all uploaded Figures meet these journal requirements.**

- Please ensure that the paper adheres to the PLOS Data Availability Policy (see [https://urldefense.com/v3/__http://journals.plos.org/plosmedicine/s/data-availability__;!!GuAItXPztq0!krEX2Kzff5jHCVa7cl_tyAY-WubHw7PvS3HNLv9odEsXmKbjaJAqRCHamTsXE1X7OC8Br4D8AVu3pdiQBBs$](https://urldefense.com/v3/__http:/journals.plos.org/plosmedicine/s/data-availability__;!!GuAItXPztq0!krEX2Kzff5jHCVa7cl_tyAY-WubHw7PvS3HNLv9odEsXmKbjaJAqRCHamTsXE1X7OC8Br4D8AVu3pdiQBBs$) ), which requires that all data underlying the study's findings be provided in a repository or as Supporting Information.

**As mentioned above, we have now uploaded all data underlying the study’s findings to GitHub, which we now clarify in our Data Sharing statement under our Declarations section.**

**(Declarations, Page 24, Lines 446-447):**

**“Deidentified data, a supporting data dictionary, and statistical code for the analyses in this paper are publicly available at** [**https://github.com/Peer-PrEP-trial-Hutch/Peer-PrEP-main-manuscript**](https://github.com/Peer-PrEP-trial-Hutch/Peer-PrEP-main-manuscript)**.”**

- We expect all researchers with submissions to PLOS in which author-generated code underpins the findings in the manuscript to make all author-generated code available without restrictions upon publication of the work.

**We have additionally uploaded the author-generated code that underpins the manuscript findings to GitHub and clarified this in our Declarations section.**

**(Declarations, Page 24, Lines 446-447):**

**“Deidentified data, a supporting data dictionary, and statistical code for the analyses in this paper are publicly available at** [**https://github.com/Peer-PrEP-trial-Hutch/Peer-PrEP-main-manuscript**](https://github.com/Peer-PrEP-trial-Hutch/Peer-PrEP-main-manuscript)**.”**

*FORMATTING – GENERAL*

- Abstract: Please structure your abstract using the PLOS Medicine headings (Background, Methods and Findings, Conclusions).

**Our manuscript Abstract is structured using the PLOS Medicine headings.**

- At this stage, we ask that you include a short, non-technical Author Summary of your research to make findings accessible to a wide audience that includes both scientists and non-scientists.

**We have included with this resubmission an Author Summary of our research.**

**(Author Summary, Pages 2-3, Lines 25-51):**

**“Why was this study done?**

- **Adolescent girls and young women (AGYW) throughout Africa have high risk of HIV acquisition and low HIV pre-exposure prophylaxis (PrEP) use.**
- **AGYWs have close peer networks and prior studies have found that most AGYW who initiate PrEP do so following peer referral.**
- **To increase PrEP initiation among AGYW likely to benefit, we wanted to test the effect of enhancing peer referral with formal referral training and HIV self-testing (HIVST) delivery in Kenya.**

**What did the researchers do and find?**

- **We conducted a two-arm randomized controlled trial of peer networks in which AGYW PrEP users (i.e., index peers) were encouraged to refer four peers to PrEP (i.e., referred peers) and randomized to either standard or enhanced peer referral inclusive of HIVST delivery.**
- **Three months following referral, there were no differences in PrEP initiation or continuation (i.e., month one refills) among referred peers that received standard versus enhanced referral, but recent HIV testing (any form following referral) was significantly higher among those who received enhanced referral.**
- **While enhanced peer referral was perceived as acceptable, feasible, and appropriate and delivered with high fidelity in this study, it cost roughly eight-times the cost of standard referral for each peer referred to PrEP.**

**What do the findings mean?**

- **Delivering HIVST as part of enhanced peer PrEP referral did not increase PrEP initiation among AGYW who could benefit in Kenya.**
- **Peer-delivered HIVST, however, did increase HIV testing uptake, presenting opportunities to layer other implementation strategies (e.g., remote nurses, chatbots, peer-delivered PrEP) to potentially increase AGYW’s engagement in HIV prevention services.”**
- Please express the main results with 95% CIs as well as p-values.

**We have updated all primary and secondary effect size estimates to include p-values in addition to 95% CIs and now report 95% CIs per the suggested PLOS Medicine guidelines.**

**(Abstract, Page 4, Lines 71-75):**

**“At follow up, there were no statistically significant differences between study groups in PrEP initiation (enhanced: 30%, 41/137; standard: 41%, 41/104; RD -6%, 95% CI [-26%, 11%], p=0.51) nor PrEP continuation (enhanced: 13%, 17/137; standard: 11%, 11/104; RD 1%, 95% CI [-10%, 13%], p=0.82) among referred peers.”**

**(Results, Page 16, Lines 311-323):**

**“At follow-up, roughly three months from index peers’ enrollment (standard group) or training (enhanced group), there were no significant differences in PrEP initiation or continuation among referred peers, Figure 2. PrEP initiation among referred peers was 30% (41/137) in the enhanced group and 41% (41/104) in the standard (RD -6%, 95% CI [-26%, 11%], p=0.51; estimated ICC 0.66), while PrEP continuation was 13% (17/137) in the enhanced group and 11% (11/104) in the standard (RD 1%, 95% CI [-10%, 13%], p=0.82). Recent HIV testing among referred peers at follow up, however, was significantly higher in the enhanced group (95%, 125/137) compared to the standard (54%, 56/104; RD 39%, 95% CI [24%, 54%], p<0.001). According to index peers, three referred peers tested HIV-positive; all in the enhanced group. These findings remained largely consistent in our sensitivity analyses, S4 Table. In our exploratory analysis, PrEP use among index peers at the point of peer referral was significantly associated with increased PrEP initiation among referred peers (RD 22%, 95% CI [3%, 42%], p=0.03), S5 Table; no other index peer features were statistically associated with this outcome.”**

**(Results, Pages 16-17, Lines 326-337):**

**“Index peers in the enhanced group reported referring more peers to PrEP (median: 4 referred peers, IQR 3 to 4) than those in the standard (median: 3, IQR 3 to 4), but the difference between the two groups was not statistically significant (RD 0.5, 95% CI [-0.2, 1.1], p=0.09), Table 2. When we evaluated PrEP initiation, PrEP continuation, and HIV testing among referred peers reached for follow up, our findings were consistent; there were no significant differences between the two groups in PrEP initiation (RD -24%, 95% CI [-50%, 1%], p=0.07) and continuation (RD -9%, 95% CI [-25%, 8%], p=0.33), but HIV testing was significantly higher in the enhanced group compared to the standard (RD 36%, 95% CI [21%, 51%], p<0.001). There were also no significant differences between study groups in partner HIV testing among referred peers (RD 11%, 95% CI [-17%, 38%], p=0.46), PrEP adherence among referred peers (RD -7.5, 95% CI [-28.1, 13.2], p=0.47), and PrEP continuation among index peers (RD -19%, 95% CI [-40%, 2%], p=0.08). Only one referred peer in the standard group reported a partner testing HIV positive following referral.”**

- Please include page numbers and line numbers in the manuscript file. Use continuous line numbers (do not restart the numbering on each page).

**Continuous page and line numbers are included in the manuscript file.**

- Please cite the reference numbers in square brackets. Citations should precede punctuation.

**Reference numbers are cited in square brackets and precede punctuation.**

*FIGURES AND TABLES*

- Please provide titles and legends for all figures and tables (including those in Supporting Information files).

**We have added a section at the end of the paper that includes the titles and legends for all figures/tables in the Supporting Information files.**

- Please define all abbreviations used in each figure/table (including those in Supporting Information files).

**We have carefully double checked all our figures and tables to ensure all abbreviations used are clearly defined somewhere within the figure/table or footnotes.**

- Please consider avoiding the use of red and green in order to make your figure more accessible to those with color blindness.

**We confirm that none of our included figures use red or green.**

*SUPPLEMENTARY MATERIAL*

- Please note that supplementary material will be posted as supplied by the authors. Therefore, please amend it according to the relevant comments outlined here.

**We have reviewed the PLOS guidelines for supplementary materials and updated those included in the manuscript so that they are in alignment.**

*REFERENCES*

- PLOS uses the numbered citation (citation-sequence) method and first six authors, et al.

**We have used the numbered citation method and first six authors for all references.**

- Please ensure that journal name abbreviations match those found in the National Center for Biotechnology Information (NCBI) databases, and are appropriately formatted and capitalised.

**We have double checked that all journal name abbreviations match the NCBI databases and are appropriately formatted/capitalized.**

- Where website addresses are cited, please include the complete URL and specify the date of access (e.g. [accessed: 12/06/2023]).

**We have included the access date for all referenced websites.**

*RCTs*

- PLOS Medicine requires that all trials be prospectively registered in one of registries recognized by WHO. Please ensure that study registration details are included in the Methods section.

**Our trial was prospectively registered on ClinicalTrials.gov, the details of which we have included in the Methods.**

**(Methods, Page 7, Lines 122-124):**

**“The "Peer PrEP cRCT" was a hybrid type I effectiveness-implementation parallel cluster-level randomized superiority trial of AGYW peer networks (ClinicalTrials.gov: NCT04982250, registered: July 29, 2021) [18, 19].”**

- Please structure the Methods section using the following sub-headings: Study design and participants, Randomization and masking, Procedures, Outcomes, Statistical analysis.

**The Methods section is structured using the suggested sub-headings.**

- The following outcomes measures [ADD DETAILS AS NEEDED OR DELETE BULLET POINT] appear to differ between the submitted manuscript and the protocol [and/or trial registry]. Please clarify and explain all discrepancies between the paper and protocol. If the outcomes were not prespecified in the protocol, please define them in the Methods (Outcomes section) as post hoc and explain why they were added. Post-hoc comparisons should be presented as hypothesis generating rather than conclusive.

**Most outcomes reported in our paper were prespecified; we have clarified in the Methods which of these were primary, secondary, and other outcomes. The only post-hoc outcome included in the manuscript was partner testing among referred peers self-reported, which we added to help better understand our null PrEP initiation findings. We also have clarified which of our sensitivity analyses were prespecified and post hoc.**

**(Methods, Pages11-12, Lines 220-242):**

**“We report outcomes at follow-up for three study populations: a) referred peers reported by index peers (in index peer follow-up surveys); b) referred peers self-reported (in referred peer follow-up surveys); and c) index peer self-reported (in index peer follow-up surveys). Our prespecified primary outcome was PrEP initiation among referred peers reported by index peers (population a), defined as visiting a clinic or other location and being dispensed PrEP. Prespecified secondary outcomes included recent HIV testing (any form following peer referral) and PrEP continuation (refilling one month following initiation) among referred peers reported by index peers (population a), PrEP adherence (past month) among referred peers (population b), and PrEP continuation (refilling following enrollment) among index peers (population c). To assess PrEP adherence in the past month we used a validated three-item 100-point scale [26], with higher scores indicating better adherence and zero indicating no PrEP use. Additional prespecified outcomes included HIV testing, PrEP initiation, and PrEP continuation among referred peers (population b) and the number of peers referred by each index peer (population c). A post-hoc outcome was partner HIV testing following referral among referred peers (population b), which we added to help understand our null primary finding.**

**In this study, we also measured several process and implementation outcomes. Process outcomes included what steps participants engaged in and support they received following referral. Implementation outcomes included index and referred peers’ perceptions of the intervention’s acceptability, feasibility, and appropriateness, which we measured using established frameworks [27-29] or scales [29] with five-point Likert scales that assessed level of agreement with various statements. We also measured fidelity by assessing if participants received the materials appropriate for their study group assignment. Additionally, we calculated the cost (in USD) per peer that was referred to and initiated PrEP.”**

**(Methods, Page 13, Lines 262-273):**

**“Prespecified sensitivity analyses included estimating effect sizes: 1) among all potential referred peers (i.e., four per index, with missing data considered as failures); 2) among only those who initiated PrEP (PrEP continuation only); 3) with the assumption that “don’t know” responses equal success; 4) using multiple imputation to predict “don’t know” responses (Markov Chain Monte Carlo models under the assumption of missingness at random [31]); and 5) using generalized estimating equations. All sensitivity analyses were assessed among referred peers reported by index peers (population a). As a post hoc sensitivity analysis, we estimated effect sizes among referred peers (population b) after removing those who reported using PrEP at the point of referral.**

**As a post hoc exploratory analysis, we conducted bivariable regression models that controlled for study group and adjusted for clustering at the index peer level to understand features of index peers associated with PrEP initiation among referred peers (population a). Features associated with the outcome at the alpha=0.05 level were considered statistically significant.”**

- Please ensure that all prespecified outcomes (primary, secondary, and exploratory) are listed in the Methods/Outcomes section and indicate whether there are outcomes that are not presented in the current report.

**All prespecified outcomes are listed in the outcomes sub-section in the Methods and reported in the Results.**

- Please specify the dates (Month Day, Year) during which study enrollment and follow up occurred.

**In the Results, we have ensured the dates (Month Day, Year) in which study enrollment and follow up occurred are clearly specified.**

**(Results, Page 14, Lines 284-285):**

**“From May 3, 2023 to February 16, 2024, we screened 316 index peers and enrolled and randomized 82: 40 to the enhanced group and 42 to the standard, Figure 1.”**

**(Results, Page 15, Line 293):**

**“All index peers were reached for follow-up from September 11, 2023 to June 5, 2024.”**

**(Results, Page 15, Lines 299-301):**

**“Over the trial duration, we obtained contact information for 60% (145/241) of the referred peers, 116 (48%) of whom we reached for follow-up and 83 (34%) of whom we enrolled from November 21, 2023 to August 27, 2024.”**

- Please include absolute numbers wherever you report percentages; eg, n/N (%)

**We have double checked that we report absolute numbers wherever we report percentages in the Results.**

- Please present the safety data for the study including numbers of specific events and whether or not adverse events are thought to be related to treatment. AEs should be reported in the abstract, per CONSORT and CONSORT-Harms.

**In the Results, we mention that no serious adverse events were reported over the study period.**

**(Results, Page 20, Lines 361-362):**

**“Two social harms (both verbal abuse among index peers, one in each group) and no serious adverse events were reported over the trial duration.”**

- Please complete the CONSORT checklist and ensure that all components of CONSORT are present in the manuscript, including how randomization was performed, allocation concealment, blinding of intervention, definition of lost to follow-up, power statement. When completing the checklist, please use section and paragraph numbers, rather than page numbers.

**We have updated the submitted CONSORT checklist to align with the latest version of this manuscript and specified the section of the paper in which CONSORT components are described using section and paragraph numbers.**

- Please report your abstract according to CONSORT for abstracts, following the PLOS Medicine abstract structure (Background, Methods and Findings, Conclusions).

**Our Abstract is reported per PLOS Medicine’s abstract structure guidelines.**

- If your trial had to undergo important modifications in response to extenuating circumstances, please complete the CONSERVE-CONSORT checklist and provide in your Supporting Information. When completing the checklist, please use section and paragraph numbers, rather than page numbers.

**There were not extenuating circumstances that impacted implementation of our trial and thus the CONSERVE-CONSORT checklist is not relevant.**

- In keeping with our commitment to Open Science, please include the study protocol document and analysis plan (including any amendments) as Supporting Information to be published with the manuscript if accepted.

**Our Study Protocol and SAP are included as Supporting Information with this resubmission and also available via CT.gov.**

**(Methods, Page 7, Lines 122-125):**

**“The "Peer PrEP cRCT" was a hybrid type I effectiveness-implementation parallel cluster-level randomized superiority trial of AGYW peer networks (ClinicalTrials.gov: NCT04982250, registered: July 29, 2021) [18, 19]. Details of the trial protocol and statistical analysis plan (SAP) are described elsewhere (S1 Protocol and S2 SAP).”**

- Please note that PLOS Medicine requires prospective, public registration of a data sharing plan (as part of mandatory clinical trials registration) for all clinical trials that began enrollment on or after January 1, 2019, in accordance with ICMJE requirements.

**Our data sharing plan is included under the IDP Sharing Statement on ClinicalTrials.gov for this study. In October 2025, we received IRB approval to share deidentified study data in a public repository and we revised our ClinicalTrials.gov IDP Sharing Statement to reflect our plans to upload the data and code to GitHub. It make take several weeks, however, for the updated ClinicalTrials.gov record to be published.**
