# Supplementary material for: Effect of enhanced peer PrEP referral with HIV self-testing delivery among young Kenyan women: A randomized controlled trial of peer networks
Source: PLoS Med. 2026 Mar 30;23(3):e1005023. doi: 10.1371/journal.pmed.1005023 (PMC13046272; doi:10.1371/journal.pmed.1005023)
Supplement: Attachment — Submitted filename: Peer PrEP cRCT_RR_2026.01.02.docx [file pmed.1005023.s011.docx]

**RESPONSE TO REVIEWERS**

2^nd^ January 2026

REVIEWER 1:

Thank you to the authors for the response to my previous comments—their revision has addressed them well.

**We are glad the reviewer is satisfied with our revisions made in response to their prior comments.**

I did find it surprising to see the very large estimated ICC (0.66). After thinking about it, it makes sense if the number of referrals per peer was relatively low and the peer had a big influence (so that the rate of initiation was highly correlated within the cluster). I'll leave it to the authors if they wish to add more about this to the discussion point about the high ICC as it may be relevant to future work.

**We were also surprised by the high ICC. In the Discussion, we have expanded on the implication this finding may have on future studies examining interventions within small AGYW peer networks.**

**(Discussion, Pages 23, Lines 435-440):**

**“Finally, the observed ICC (0.66) was substantially than that anticipated in the study design (0.05), which reduced our statistical power to detect significant differences in PrEP initiation between study groups. This finding has important implications for the design of future studies examining interventions within small AGYW peer networks, as higher ICCs require more clusters or larger sample sizes to maintain adequate power.”**

REVIEWER 2:

The authors have responded appropriately to the comments. I don’t have any concerns with proceeding with the publication.

**We thank Reviewer 2 for their time spent reviewing our manuscript, which has improved because of their comments.**

REVIEWER 3:

This is a revision of a two-arm RCT examining PrEP uptake and HIV testing among adolescent and young girls in Kenya. Strengths include focusing on a group with an urgent need for HIV prevention, examining peer-based strategies, and a high follow-up rate among index participants. However, there were several notable weaknesses, including differential loss to follow up in the referred peer group, short follow-up period, and limited innovation. The intervention was expensive as well, raising some questions about what inferences can be made from this data in the current environment. While the revision was very responsive to comments, I have doubts about whether this is (yet) up to PLoS Medicine standards.

**We thank Reviewer 3 for their prior comments, which improved the manuscript, and have responded to their additional comments in detail below.**

*MAJOR COMMENTS*

1. Innovation in the context of PrEP limited. There have been peer-based PrEP interventions before. There have also been many studies looking at peer-based HIVST interventions. In light of this research, what is new about this research? The innovation could be more clearly articulated.

**This research is innovative because it pairs peer-delivered HIVST kits with formalized peer PrEP referral strategies, obtained from an in-person group training session, to motivate PrEP initiation among Kenyan AGYW. While there have been other peer-delivered HIVST interventions, they primarily have focused on HIV testing as an endpoint or identification of individuals living with HIV. However, most people who test for HIV will test negative. Thus, this intervention aimed to utilize trusted AGYW peers with knowledge about PrEP and linkage to PrEP services to help motivate close friends/peers to initiate PrEP following HIVST. While other interventions have explored peer-based PrEP interventions for AGYW in Africa, few have integrated peer-delivery of HIVST kits and most have focused on the use of select PrEP champions (with large, distal peer networks) versus several trained peers (with smaller, closer peer networks). We have elaborated on the innovative parts of this study in the Introduction and also discuss these in the Discussion.**

**(Introduction, Page 6-7, Lines 97-116):**

**“The opinion of peers often influences the health behaviors and preferences of populations with strong social networks, including AGYW. In Africa, most AGYW who initiate PrEP do so because of informal peer referral; in the HPTN 082 open-label PrEP study, 62% of 400 AGYW who initiated PrEP in South Africa and Zimbabwe did so due to peer encouragement [10]. Peer-delivered interventions have successfully increased engagement in HIV prevention services among other populations with strong social networks, including men who have sex with men [11] and female sex workers [12, 13], yet remain underexplored among AGYW. HIV prevention interventions that have engaged AGYWs in Africa to date have primarily utilized “PrEP champions” [14], who engage in outreach activities to large peer networks, inclusive of individuals with whom they may or may not have personal connections. Peer delivery of HIV prevention interventions within small, established AGYW peer networks may enable more targeted outreach to individuals known to engage in behaviors associated with HIV risk acquisition who are most likely to benefit.**

**Strategies that could support peer-delivered HIV prevention interventions within small AGYW peer networks include HIV self-testing (HIVST) and index peer education. HIVST kits have been demonstrated to increase HIV testing uptake and frequency in diverse populations and settings but have been underutilized to support PrEP initiation [15-17]—which could likely benefit many individuals who learn they are HIV-negative following self-testing. To help facilitate linkage to PrEP following HIVST, trusted peers who have used PrEP and been trained on PrEP use, safety, and referral could provide counseling at the point of HIVST delivery that addresses known AGYW PrEP initiation barriers, such as PrEP stigma, knowledge, and access.”**

**(Discussion, Page 21, Lines 388-394):**

**“In this intervention, the use of HIVST to promote linkage to prevention services was novel, as most of HIVST interventions to date have primary focused on identifying individuals living with HIV and facilitating linkage to treatment services [41-44]. However, most individuals who complete HIVST at home will test negative and may feel motivated to change their behavior and/or engage in HIV prevention interventions to maintain their HIV negative status [45, 46]. Consistent with other peer-delivered HIVST interventions in populations with strong social networks [14-16], this intervention was associated with increased HIV testing uptake among AGYW. …”**

2. Rationale for control arm unclear. The standard peer referral group would still need to have basic information about HIVST and the kits themselves. I am confused about the decision to not give informational brochures or kits to participants in this arm of the trial. A sentence explaining this more completely would be helpful.

**The main objective of the study was to identify the effects of the enhanced peer PrEP referral intervention on PrEP initiation compared to standard peer PrEP referral. The main enhancements to standard peer PrEP referral introduced in the intervention arm of this study were peer delivery of HIVST kits and index peer training on PrEP and PrEP referral strategies. To isolate the effects of these enhancements, index peers in the control arm were thus not given HIVST kits, training, or educational PrEP/HIVST brochures that were supplemental to the training. We aimed for the control arm of the study to reflect the peer PrEP referral strategies that would be available without additional inputs at most public healthcare facilitates in Kenya. We have made edits to clarify this in the Methods.**

**(Methods, Pages 10-11, Lines 201-206):**

**“Standard peer referral. Index peers assigned to this study group did not receive formal training on PrEP referral strategies, informational brochures on PrEP and HIVST, or HIVST kits. At enrollment, they were simply encouraged to discuss PrEP and refer peers using the usual approach they would for other sexual health interventions, such as contraception, and were provided the same MOH-style PrEP referral cards (n=4) as the intervention group, reflecting standard peer referral strategies available for AGW at most public healthcare facilities in Kenya.”**

*MINOR COMMENTS*

1. (Line 37): "Inclusive of HIVST delivery" sounds a bit odd here. Consider instead: enhanced peer referral for HIVST and PrEP.

**We have edited our description of the enhanced peer referral arm in the Author Summary to eliminate use of the language the reviewer found odd. Since in the last bullet of the prior sub-section, we clarify what enhanced peer referral entailed, we dropped further description of enhanced peer referral in this sub-section to avoid repetition.**

**(Author Summary, Page 2, Lines 35-37):**

**“We conducted a two-arm randomized controlled trial of peer networks in which AGYW PrEP users (i.e., index peers) were encouraged to refer four peers to PrEP (i.e., referred peers) and randomized to either standard or enhanced peer referral.”**

2. (Lines 49-51 and 83-83): Not sure that I understand how finding that peer's motivated HIVST uptake has implications for nurses of chatbots being integrated into PrEP. Consider simplifying this around the data presented in both the highlights and abstract sections of the paper.

**We have added clarifying points to the Author Summary to describe how the addition of other implementation strategies with peer-delivered HIVST kits might address known AGYW barriers to PrEP uptake and improve PrEP initiation. We have simplified the Abstract to be less specific about these potential strategies. In the Discussion, we describe in more detail how these strategies could enhance peer-delivered HIVST interventions to improve PrEP initiation among AGYW.**

**(Author Summary, Page 3, Lines 48-51):**

**“Peer-delivered HIVST, however, was with increased HIV testing uptake, presenting opportunities to layer other strategies—such as remote nurse support, chatbots, peer-delivered PrEP—at the point of testing to increase PrEP awareness and access among AGYW and potentially improve PrEP uptake.”**

**(Abstract, Page 5, Lines 79-82):**

**“Enhanced peer PrEP referral with training and HIVST delivery did not increase PrEP initiation among Kenyan AGYW but was associated with increased HIV testing, suggesting opportunities to combine peer-delivered HIVST with additional implementation strategies to improve AGYW’s PrEP engagement.”**

**(Discussion, Pages 23-24, Lines 446-454):**

**“While peer PrEP referral enhanced with training and HIVST delivery did not increase PrEP initiation compared to standard referral, it was associated with increased HIV testing—the first step of the HIV prevention and treatment cascade. Thus, intervention adaptations that might better support linkage to HIV services should be considered, including the addition of implementation strategies that might enhance the quality of counseling services received at the point of referral—such as facilitating access to telehealth visits [35, 48] or appropriately trained artificial intelligence chatbots [49]—or bring services directly to where AGYW are testing—such as peer-delivered or courier-delivered PrEP [34, 35, 50], likely following a telehealth visit.”**

3. (Line 53): There has been a pushback from African scholars against using SSA as a term. Would consider using "Africa" here, if possible and appropriate.

**We have eliminated use of “sub-Saharan Africa” throughout the manuscript, replacing all mentions of this team with “Africa”.**

**(Author Summary, Page 2, Lines 27-28):**

**“Many adolescent girls and young women (AGYW) throughout Africa have high risk of HIV acquisition and low HIV pre-exposure prophylaxis (PrEP) use.”**

**(Abstract, Page 4, Lines 53-54):**

**“Adolescent girls and young women (AGYW) in Africa experience high HIV acquisition risk and low engagement in prevention services.”**

4. (Line 58): RCT of peer networks is confusing here. Consider instead: " RCT of a peer-based intervention (NCTxx)…

**We have revised how we introduce the study design in the “Methods and Findings” sub-section of the Abstract to enhance clarify.**

**(Abstract, Page 4, Lines 57-59):**

**“Methods and Findings: We conducted a randomized controlled trial (ClinicalTrials.gov: NCT04982250) of AGYW peer networks in Central Kenya. Index peers aged 16-24 years who had used oral PrEP in the past 12 months were randomized 1:1 to: ..”**

5. (Line 89): Can remove less than or equal to and define AGYW as simply "16 to 24 years"

**We have clarified our description of the AGYW eligible for enrollment in this study per the reviewer’s suggestion.**

**(Introduction, Page 6, Lines 87-89):**

**“Adolescent girls and young women (AGYW, 16 to 24 years) in many African settings have high risk of HIV acquisition [1, 2] and low engagement in highly effective oral HIV pre-exposure prophylaxis (PrEP) services [3-5].”**

6. (Line 135): It might be easier here to refer to "alters" and "indexes"—using conventional network terms (and realizing that these will need to be defined for a PMed audience). Using the terms "index" and "index peers" is somewhat confusing. Also, the peers are sometimes called "index peers" and at other times called "referred peers." It is important to harmonize the nomenclature.

**We appreciate this suggestion but have opted to stick with our current terms, which are the same as those utilized in our study protocol, trial registration website (ClinicalTrials.gov), and Statistical Analysis Plan. However, we have carefully reviewed the manuscript to ensure that we consistently refer to “index peers” and “referred peer” (i.e., those referred to PrEP services by index peers) as such. The distinction between index and referred peers is described in detail in the Methods.**

**(Methods, Page 8, Lines 134-140):**

**“The AGYW peer networks (i.e., clusters) in this trial consisted of one index peer and up to four referred peers. Eligible index and referred peers were females aged ≥16-24 years, with those <18 years meeting the criteria for emancipated minors (married, mothers, pregnant, or household heads [23, 24]). Eligible index peers additionally had used PrEP in the past 12 months, could identify four peers who could benefit from PrEP, were literate, and were not enrolled in another HIV-related study or program. Eligible referred peers additionally had been referred to PrEP by an index peer.”**

7. (Line 150): Revise "competition" to "completion."

**We have updated our description of what participants’ were compensated for survey completion so that the correct word is now in use.**

**(Methods, Pages 8-9, Lines 154-156):**

**“All enrolled participants completed informed consent—either written or verbal (select referred peers only)—administered by Kenyan research assistants (RAs) and received 300 KES (~$1.90 USD) for completion of each survey.”**

8. (Line 425): There is also a literature showing that.

**The citation we added for the statement that delivering MOH PrEP referral cards alone—a light touch intervention—may be an effective strategy for engaging AGYW in PrEP came from a study among AGYW in Kenya that demonstrated a peer referral program that handed out HIV testing and counseling referral cards increased the proportion of first-time testers.**

**(References, Page 31, Lines 651-653):**

50. **Ong’wen P, Samba BO, Moghadassi M, Okoko N, Bukusi EA, Cohen CR, Wolf HT. Chain peer referral approach for HIV testing among adolescents in Kisumu County, Kenya. *AIDS Behav*. 2020;24(2):484-490.**
9. (Table titles): Recommend including the location, year, and sample size in each table title.

**We appreciate this suggestion but prefer the description of Table titles as they are. Each table clearly designates the sample size underlying the results presented and details on the study location and year can be found elsewhere in the manuscript. We are happy to make these suggested revisions to the Table titles if the Editors think this is appropriate.**

10. (Figure nomenclature): What is called Figure 1 now is actually a table.

**Figure 1 is a separate diagram distinct from Table 1. Since Tables, but not Figures, were embedded in the submitted manuscript—per PLOS Medicine guidelines—it could appear that our Figure 1 description is that our Table 1, which is presented directly below.**

**(Figure 1):**

11. (Protocol): The protocol attached in the supplement would benefit from having a section that lists changes. For example, it seems like there were relatively recent changes on NCT. An inventory of changes and their rationale would be useful here.

**We added a “Key Changes” page near the beginning of the Protocol that lists changes made over implementation of the Peer PrEP cRCT. Some of the more recent changes were attributable to delays in coordination between the trial statistician and project coordinators; the SAP, which includes definitions of all study outcomes, was finalized prior to the analysis and unblinding of any trial findings.**

**(S1 Protocol, Page 2, Lines 50-76):**

EDITOR REQUESTS:

*GENERAL*

- Please review your text for claims of novelty or primacy (e.g. 'for the first time') and remove this language. In addition, please check that any use of statistical terms (such as trend or significant) are supported by the data, and if not please remove them.

**We have double checked the manuscript and confirm that no claims of novelty or primacy are in use, and that all statistical terms used are supported by the data.**

- (Statistical reporting): Please revise throughout the manuscript, including tables and figures.
  - Please report statistical information as follows to improve clarity for the reader ""22% (95% CI [13,28]; p</=)"".

**We confirm that all statistical information is presented per PLOS Medicine guidelines throughout the manuscript, including in Tables, Figures, and Supporting Information Files. To align with these guidelines, we made some minor updates to Table 2, S4 Table, and S5 Table.**

**(Table 2, Pages 17-18):**

**(S4 Table):**

**(S5 Table):**

- Please separate upper and lower bounds with commas instead of hyphens as the latter can be confused with reporting of negative values.

**We confirm that all upper and lower bounds are separated with commas instead of hyphens throughout the manuscript.**

- Please repeat statistical definitions (HR, CI etc.) for each set of parentheses."

**We confirm that statistical definitions—RDs in our study—are included in each set of parentheses. For example, in Lines 335-337: “While index peers in the enhanced group reported referring more peers to PrEP (median: 4 referred peers, IQR 3 to 4) than those in the standard (median: 3, IQR 3 to 4), the intervention was not associated with more PrEP referrals (RD 0.5, 95% CI [-0.2, 1.1], p=0.09), Table 2.”**

- In the last sentence of the Abstract, Methods and Findings section, please describe the main limitation(s) of the study's methodology.

**We have added the main study limitations to the last sentence of the sub-section “Methods and Findings” in the Abstract.**

**(Abstract, Page 5, Lines 77-78):**

**“Relying on index peers to report outcomes for referred peers was a study limitation.”**

- In the Abstract, please include the important dependent variables that are adjusted for in the analyses.

**In the “Methods and Findings” sub-section of the Abstract, we have added details on the models used to estimate our effect sizes, including a description of all dependent variables adjusted for in the models.**

**(Abstract, Page 4, Lines 65-66):**

**“Risk differences (RDs) were estimated using generalized linear mixed-effects regression models, with study group fixed effects and index peer random effects.”**

*FUNDING STATEMENT*

- The funding statement should include: specific grant numbers, initials of authors who received each award, URLs to sponsors’ websites. Also, please state whether any sponsors or funders (other than the named authors) played any role in study design, data collection and analysis, the decision to publish, or preparation of the manuscript. If they had no role in the research, include this sentence: “The funders had no role in study design, data collection and analysis, decision to publish, or preparation of the manuscript.”

**We have updated the study funding statement to ensure it includes specific grant numbers, initials of authors who received each award, and URLs to the sponsors’ websites. A statement confirming that the funders had no role in the research was already included.**

**(Declarations, Page 25, Lines 458-461):**

**“Financial disclosures**

**This work was supported by the National Institute of Mental Health (R00 MH121166 to KFO; https://www.nimh.nih.gov/). The funders had no role in study design, data collection and analysis, decision to publish, or preparation of the manuscript.”**

- It appears that one or more study authors is affiliated with one or more of the agencies that funded the study. Thus, the statement “The funders had no role in study design, data collection and analysis, decision to publish, or preparation of the manuscript” does not apply. Please revise the Financial Disclosure accordingly, as in "[Author name] is [author's role] at [funding agency]. The funders had no other role in study design…..”

**We no longer list Dr. Ortblad’s Fred Hutch start-up package as a source of fundings for this project, which was sponsored by NIMH (see updated “Financial disclosures” statement above). Now none of the study authors are affiliated with the agency that funded the study.**

*COMPETING INTERESTS STATEMENT*

- All authors must declare their relevant competing interests per the PLOS policy, which can be seen here: [https://urldefense.com/v3/__https://journals.plos.org/plosmedicine/s/competing-interests__;!!GuAItXPztq0!n8wkm5a5fwk2FeG3KmkqPwZLyrinstUexYsIwfoX8VqTAhNZK9V2UKG6jyF8hmIjRZJQv8YxeZdUuzlnPkM$](https://urldefense.com/v3/__https:/journals.plos.org/plosmedicine/s/competing-interests__;!!GuAItXPztq0!n8wkm5a5fwk2FeG3KmkqPwZLyrinstUexYsIwfoX8VqTAhNZK9V2UKG6jyF8hmIjRZJQv8YxeZdUuzlnPkM$)  For authors with ties to industry, please indicate whether any of the interests has a financial stake in the results of the current study.

**We have clarified in our “Competing interests” statement that author JMB, an employee of Gilead Sciences, has no financial stake in the results of the current study.**

**(Declarations, Page 25, Lines 462-466):**

**“Competing interests**

**The authors of this manuscript have the following competing interests: JMB is an employee of Gilead Sciences, outside the present work, and has no financial stake in the results of this study; KFO is an Academic Editor on PLOS Medicine’s editorial board. None of the other authors declare any conflicts of interest.”**

- Please add this statement to the manuscript’s Competing Interests: “[Initials] is an Academic Editor on PLOS Medicine’s editorial board.”

**Additionally, we have clarified that author KFO is an Academic Editor for PLOS Medicine (see revised statement above).**

*DATA AVAILABILITY*

- PLOS defines the “minimal data set” to consist of the data set used to reach the conclusions drawn in the manuscript with related metadata and methods, and any additional data required to replicate the reported study findings in their entirety. Authors do not need to submit their entire data set, or the raw data collected during an investigation. Please submit the following data:
  - The values behind the means, standard deviations and other measures reported; the values used to build graphs; the points extracted from images for analysis.

**We confirm that the values behind the presented means, standard deviations, and other measures reported in all Tables and Figures are included in the dataset available for download via GitHub.**

- The Data Availability Statement (DAS) requires revision. For each data source used in your study:
  a) If the data are freely or publicly available, note this and state the location of the data: within the paper, in Supporting Information files, or in a public repository (include the DOI or accession number).

**We have revised our “Data sharing” statement to clarify that the data used to generate the findings in this manuscript are freely and publicly available.**

**(Declarations, Page 25, Lines 467-470):**

**“Data sharing**

**All data presented in this paper are freely and publicly available. The deidentified study data, a supporting data dictionary, and the statistical code use for the analyses in this paper can all be found at:** [**https://github.com/Peer-PrEP-trial-Hutch/Peer-PrEP-main-manuscript**](https://github.com/Peer-PrEP-trial-Hutch/Peer-PrEP-main-manuscript)**.”**

b) If the data are owned by a third party but freely available upon request, please note this and state the owner of the data set and contact information for data requests (web or email address). Note that a study author cannot be the contact person for the data.

**Not applicable for the data presented in this study.**

c) If the data are not freely available, please describe briefly the ethical, legal, or contractual restriction that prevents you from sharing it. Please also include an appropriate contact (web or email address) for inquiries (again, this cannot be a study author).

**Not applicable for the data presented in this study.**

*FIGURES*

- Please provide titles and legends for all figures and tables (including those in Supporting Information files). Please define all acronyms used in each figure or table in its corresponding legend.

**The titles for all Figures and Tables—including those in the Supporting Information files—are included throughout the manuscript. All acronyms used were clearly defined in the Tables embedded throughout the manuscript and those in the Supporting Information; we have updated the Figures with clear descriptions of all acronyms used.**

**(Figure 1):**

**(Figure 2):**

- Please ensure that where relevant, Figures include 95% CIs.

**We have ensured that where relevant, all figures include 95% CIs.**

*CLINICAL TRIALS*

- Please complete the CONSORT 2025 checklist and ensure that all components of CONSORT 2025 are present in the manuscript, including how randomization was performed, allocation concealment, blinding of intervention, definition of lost to follow-up, power statement. When completing the checklist, please use section and paragraph numbers, rather than page numbers. The checklist should be included as supporting information, and should be cited in the article.

**We have completed the CONSORT 2025 checklist (S6 CONSORT Checklist) and confirm that all CONSORT components are presented in our revised manuscript. We have updated the checklist so that section and paragraph numbers, rather than page numbers, are used to designate where the various CONSORT components are reported. We now reference the CONSORT checklist in the manuscript.**

**(Results, Page 20, Lines 375-376):**

**“This manuscript reports all components of the CONSORT 2025 checklist, for details see S6 CONSORT Checklist.”**

**(S6 CONSORT checklist, select section):**

- PLOS Medicine requires that all trials be prospectively registered in one of registries recognized by WHO. Please provide information on study registration in the Methods section.

**Information on study registration is described at the beginning of the Methods section.**

**(Methods, Page 7, Lines 127-130):**

**“*Study design and participants***

**The "Peer PrEP cRCT" was a hybrid type I effectiveness-implementation parallel cluster-level randomized superiority trial of AGYW peer networks (ClinicalTrials.gov: NCT04982250, registered: July 29, 2021) [18, 19].”**

- In accordance with ICMJE requirements, PLOS Medicine requires prospective, public registration of a data sharing plan (as part of mandatory clinical trials registration) for all clinical trials that began enrollment on or after January 1, 2019.

**The data sharing plan for this study was prospectively reported on ClinicalTrials.gov:** [**https://clinicaltrials.gov/study/NCT04982250#study-plan**](https://clinicaltrials.gov/study/NCT04982250#study-plan)

**(ClinicalTrials.gov, NCT04982250):**

- Please present the safety data for the study including numbers of specific events and whether or not adverse events are thought to be related to treatment.

**We have clarified that the two social harms reported during the study duration were both related to study participation. In both reports, index peers were called names (verbal abuse) from a peer during the referral process.**

**(Results, Page 20, Lines 372-374):**

**“Two social harms (both verbal abuse among index peers, one in each group) and no serious adverse events were reported over the trial duration; both social harms were related to study participation.”**

- (Causal language): In trials, there is usually a distinction in the language in terms of causal vs associational for primary and secondary trial outcomes. It would be beneficial to use associational language in the discussion and other sections for secondary outcomes.

**Throughout the manuscript, we now use associational vs. causal language for our description of secondary trial outcomes.**

**(Author Summary, Page 2, Lines 38-41):**

**“Three months following referral, enhanced peer referral did not increase PrEP initiation among referred peers compared to standard peer referral, and was not associated with PrEP continuation (i.e., month one refills). However, the intervention was associated with increased recent HIV testing (any form following referral).”**

**(Author Summary, Page 3, Lines 48-51):**

**“Peer-delivered HIVST, however, was associated with increased HIV testing uptake, presenting opportunities to layer other strategies—such as remote nurse support, chatbots, peer-delivered PrEP—at the point of testing to increase PrEP awareness and access among AGYW and potentially improve PrEP uptake.”**

**(Abstract, Page 4, Lines 70-74):**

**“At follow up, there were no significant differences in PrEP initiation between the enhanced group (30%, 41/137) and the standard (41%, 41/104; RD -6%, 95% CI [-26%, 11%], p=0.51). Enhanced peer referral was not associated with PrEP continuation (RD 1%, 95% CI [-10%, 13%], p=0.82) but was associated with increased recent HIV testing (RD 39%, 95% CI [24%, 54%], p<0.001).”**

**(Abstract, Page 5, Lines 79-82):**

**“Enhanced peer PrEP referral with training and HIVST delivery did not increase PrEP initiation among Kenyan AGYW but was associated with increased HIV testing, suggesting opportunities to combine peer-delivered HIVST with additional implementation strategies to improve AGYW’s PrEP engagement.”**

**(Figure 2 Title, Page 17, Lines 332-334):**

**“The effect/association of enhanced versus standard peer referral on HIV testing, PrEP initiation, and PrEP continuation among referred three months later, as reported by index peers.”**

**(Results, Page 16, Lines 319-328):**

**“At follow-up, roughly three months from index peers’ enrollment (standard group) or training (enhanced group), there were no statistically significant differences in PrEP initiation among referred peers; with index peers reporting that 30% (41/137) of referred peers in the enhanced group and 41% (41/104) in the standard initiated PrEP (RD -6%, 95% CI [-26%, 11%], p=0.51; estimated ICC 0.66), Figure 2. Additionally, enhanced peer referral was not associated with PrEP continuation among referred peers at follow-up (enhanced: 13%, 17/137; standard: 11%, 11/104; RD 1%, 95% CI [-10%, 13%], p=0.82) but was associated with increased recent HIV testing (enhanced: 95%, 125/137; standard: 54%, 56/104; RD 39%, 95% CI [24%, 54%], p<0.001). According to index peers, three referred peers tested HIV-positive: all in the enhanced group. These findings remained largely consistent in our sensitivity analyses, S4 Table.”**

**(Table 2 Title, Page 17, Line 348):**

**“The association of enhanced versus standard peer referral on secondary and exploratory outcomes among referred and index peers three months later”**

**(Results, Page 17, Lines 335-347):**

**“While index peers in the enhanced group reported referring more peers to PrEP (median: 4 referred peers, IQR 3 to 4) than those in the standard (median: 3, IQR 3 to 4), the intervention was not associated with more PrEP referrals (RD 0.5, 95% CI [-0.2, 1.1], p=0.09), Table 2. When we evaluated PrEP initiation, PrEP continuation, and HIV testing among referred peers reached for follow up, our findings were consistent: there were no significant differences in PrEP initiation between the study groups (RD -24%, 95% CI [-50%, 1%], p=0.07), and enhanced peer referral was not associated with PrEP continuation (RD -9%, 95% CI [-25%, 8%], p=0.33) but was associated with increased recent HIV testing (RD 36%, 95% CI [21%, 51%], p<0.001). Additionally, enhanced peer referral was not associated with partner HIV testing among referred peers (RD 11%, 95% CI [-17%, 38%], p=0.46), PrEP adherence among referred peers (RD -7.5, 95% CI [-28.1, 13.2], p=0.47), and PrEP continuation among index peers (RD -19%, 95% CI [-40%, 2%], p=0.08). Only one referred peer in the standard group reported a partner testing HIV positive following referral.”**

**(Discussion, Page 21, Lines 378-381):**

**“Among Kenyan AGYW at risk of HIV acquisition, enhanced peer PrEP referral supported with index peer training and HIVST delivery did not significantly increase PrEP initiation compared to standard, word-of-mouth peer referral. Additionally, the intervention was not associated with one-month PrEP continuation but was associated with increased recent HIV testing.”**

**(Discussion, Pages 21-22, Lines 400-408):**

**“Differences in partner HIV testing among referred peers in the two study groups might have contributed to our null PrEP initiation findings. In the enhanced group, nearly half of referred peers reported using their second HIVST kit for partner testing (42%), which may have driven the higher uptake of partner testing following peer referral in the enhanced group (46%) compared to the standard (35%); however, the intervention was not associated with increased partner testing. These findings would be consistent with other studies in Kenya and Malawi that demonstrated delivery of multiple HIVST kits to pregnant women significantly increased male partner HIV testing [47, 48]. In this study, most referred peers who engaged in partner HIV testing learned their partner was not living with HIV, which may have demotivated their PrEP interest.”**

**(Discussion, Page 22, Lines 409-419):**

**“The null findings in this study might also be attributable to differences in the characteristics of peers referred in the enhanced group compared to the standard. Although index peers in the enhanced group referred more peers to PrEP than those in the standard (though the intervention was not associated with the number of peers referred), the referred peers in the standard group might have been at higher risk of HIV acquisition than those in the enhanced. It is possible that the training and HIVST kits provided to index peers in the enhanced group created additional motivation or pressure to meet the referral target of four peers, potentially leading index peers in this group to refer individuals who were less likely to benefit from PrEP. This could have driven down the proportion of referred peers who initiated PrEP in the enhanced group versus the standard. Limited data on referred peer characteristics, however, prevented us from adequately assessing this possibility.”**

**(Discussion, Page 23, Lines 446-449):**

**“While peer PrEP referral enhanced with training and HIVST delivery did not increase PrEP initiation compared to standard referral, it was associated with increased HIV testing—the first step of the HIV prevention and treatment cascade.”**

- Please report your Abstract according to CONSORT for abstracts, following the PLOS Medicine abstract structure (Background, Methods and Findings, Conclusions).

**Our reported Abstract (Page 4, Lines 52-82) aligns with CONSORT guidelines (S6 CONSORT checklist) and aligns with the PLOS Medicine abstract structure.**

- Please include the clinical trial registry number in the Abstract.

**The clinical trial registry number is included in the Abstract. We have clarified that this number is for the registry ClinicalTrials.gov.**

**(Abstract, Page 4, Lines 57-58):**

**“Methods and Findings: We conducted a randomized controlled trial (ClinicalTrials.gov: NCT04982250) of AGYW peer networks in Central Kenya.”**
