# Supplementary material for: Effect of enhanced peer PrEP referral with HIV self-testing delivery among young Kenyan women: A randomized controlled trial of peer networks
Source: PLoS Med. 2026 Mar 30;23(3):e1005023. doi: 10.1371/journal.pmed.1005023 (PMC13046272; doi:10.1371/journal.pmed.1005023)
Supplement: Attachment — Submitted filename: Peer PrEP cRCT_RR_2026.03.03.docx [file pmed.1005023.s012.docx]

**RESPONSE TO REVIEWERS**

3^rd^ March 2026

**EDITORIAL COMMENTS**

(Overall): Please include in your ethics statement ethical approval numbers. Please also upload as an additional file the original protocol that was approved by the IRB in addition to the one with amendments.

**In the Methods, we have updated our ethics statement to add approval numbers from the Fred Hutch Cancer Center’s Institutional Review Board (IRB) and Kenya Medical Research Institute’s Scientific Ethics Review Unit (SERU). Additionally, in this section, we have uploaded as an additional file the original protocol approved by the IRB and SERU and made appropriate reference to this in the manuscript. Since both the pilot and trial of this NIMH K99/R00 award were included in the same protocol, we have included the protocol version (v1.6) that included updates post-pilot but pre-trial implementation. Of note, we have edited this protocol to remote all trademarks and clipart.**

**(Methods, Page 8-9, Lines 157-159):**

**“Our trial protocol was approved by the Scientific Ethics Review Unit at the Kenya Medical Research Institute (#4349) and the Institutional Review Board (IRB) at the Fred Hutchinson Cancer Center (#10773).”**

**(Methods, Page 8, Lines 135-136):**

**“Details of the trial protocol and statistical analysis plan (SAP) are described elsewhere: S2 Protocol Final, S3 Protocol Original, and S4 SAP.”**

(Abstract): Please report the number of participants lost to follow up in each group.

**In the Abstract, we now report the number of index peers lost to follow-up. Since we did not formally enroll referred peers until follow-up, we do not have formal lost to follow-up metric for this population.**

**(Abstract, Page 2, Lines 40-42):**

**“From May 2023 to June 2024, 316 index peers were screened and 82 enrolled/randomized (median age 22 years, IQR 20-23): 40 to the enhanced group and 42 to the standard. No index peers were lost to follow-up.”**

(Abstract): Please make it clear in the Abstract that the self-reported data from the referred peers is only based on n*=55/n*=28. We would suggest adding something along the lines of: At follow-up, self-reported outcomes for referred peers were available for 55 peers in the enhanced group and 28 peers in the standard group.

**In the Abstract, we purposely did not report the number of referred peers who self-reported outcomes because we only present effect estimates in the Abstract for pre-specified outcomes among referred peers reported by index peers: PrEP initiation (primary), PrEP continuation, and HIV testing. The outcomes self-reported among referred peers were post-hoc secondary outcomes, and thus we chose to not highlight them in the Abstract.**

(Abstract): Please state clearly in the Abstract all outcomes that are post-hoc secondary outcomes.

**We do not present in the Abstract any outcomes that are post-hoc secondary outcomes (per our response above).**

(Manuscript): Please move the mention of the CONSORT checklist from the Results to the beginning of the Methods section.

**We have moved mention of the CONSRT checklist from the Results to the beginning of the Methods.**

**(Methods, Page 7, Lines 130-131):**

**“This manuscript reports all components of the CONSORT 2025 checklist, S1 CONSORT Checklist.”**
